# Association of Dietary Patterns With Testicular Function in Young Danish Men

**DOI:** 10.1001/jamanetworkopen.2019.21610

**Published:** 2020-02-21

**Authors:** Feiby L. Nassan, Tina K. Jensen, Lærke Priskorn, Thorhallur I. Halldorsson, Jorge E. Chavarro, Niels Jørgensen

**Affiliations:** 1 Department of Environmental Health, Harvard T. H. Chan School of Public Health, Boston, Massachusetts; 2 Department of Nutrition, Harvard T. H. Chan School of Public Health, Boston, Massachusetts; 3 Department of Environmental Medicine, Institute of Public Health, University of Southern Denmark, Odense, Denmark; 4 Odense Patient Data Explorative Network (OPEN), Odense, Denmark; 5 Department of Growth and Reproduction, International Center for Research and Research Training in Endocrine Disruption of Male Reproduction and Child Health, Rigshospitalet, University of Copenhagen, Copenhagen, Denmark; 6 Centre for Foetal Programming, Department of Epidemiology Research, Statens Serum Institut, Copenhagen, Denmark; 7 Faculty of Food Science and Nutrition, School of Health Sciences, University of Iceland, Reykjavík; 8 Department of Epidemiology, Harvard T. H. Chan School of Public Health, Boston, Massachusetts; 9 Channing Division of Network Medicine, Harvard Medical School and Brigham and Women’s Hospital, Boston, Massachusetts

## Abstract

**Importance:**

Diet may play a role in testicular function, but data on how adherence to different diet patterns influences human testicular function are scarce.

**Objective:**

To determine whether adherence to specific dietary patterns is associated with testicular function in young men.

**Design, Setting, and Participants:**

This cross-sectional study included 2935 young Danish men unselected regarding fertility status who were enrolled from April 1, 2008, through May 31, 2017. Data were analyzed from July 1, 2017, to January 30, 2019.

**Exposures:**

Dietary patterns identified with principal component analysis based on responses to a validated food frequency questionnaire.

**Main Outcomes and Measures:**

Standard semen quality assessment; serum concentrations of testosterone, free testosterone, estradiol, inhibin B, follicle-stimulating hormone, luteinizing hormone, and sex hormone–binding globulin; and testicular volume measured with ultrasonography.

**Results:**

Among the 2935 participants included in the analysis, median age was 19 (interquartile range, 19-20) years and 2290 (78.0%) had normal body mass index. The 4 dietary patterns identified included Western, prudent, open-sandwich (a traditional Danish eating pattern), and vegetarianlike. The greatest adherence to the prudent pattern was associated with the highest total sperm count (median, 167 [95% CI, 146-183] million), followed by adherence to vegetarianlike (median, 151 [95% CI, 134-168] million) and open-sandwich (median, 146 [95% CI, 131-163] million) patterns. Adherence to the Western pattern was associated with the lowest total sperm count (median, 122 [95% CI, 109-138] million), which was significantly lower than sperm count in the other 3 diet patterns. After adjusting for confounders, the median total sperm count for men in the highest quintile of adherence to the Western pattern was 26 million lower (95% CI, –42 to –9 million) than for men in the lowest quintile of adherence to this pattern. Conversely, the median total sperm count of men in the highest quintile of adherence to the prudent pattern was 43 million (95% CI, 23-63 million) higher than that of men in the lowest quintile. Men with the highest adherence to the Western pattern had a lower median ratio of inhibin B to follicle-stimulating hormone (–12 [95% CI, –20 to –3]) and higher median ratio of free testosterone to luteinizing hormone (10 [95% CI, 2-19]) compared with men with lowest adherence to this pattern.

**Conclusions and Relevance:**

In this cross-sectional study, adherence to generally healthy diet patterns was associated with better semen quality, with potentially more favorable fertility potential among adult men.

## Key Points

**Question:**

What is the association of dietary patterns with testicular function in men?

**Findings:**

In a cross-sectional study of 2935 young Danish men unaware of their fertility status, higher adherence to the Western diet pattern was associated with lower sperm quality than that of men with the lowest adherence. Conversely, higher adherence to the prudent diet pattern was associated with higher sperm quality.

**Meaning:**

These findings suggest that adherence to healthy diet patterns, a potentially modifiable lifestyle factor, is associated with better semen quality and potentially more favorable fertility potential among young men.

## Teaser

This cross-sectional study assesses whether adherence to specific dietary patterns is associated with testicular function and semen quality in young Danish men.

## Introduction

Semen quality has decreased substantially in the last few decades. According to a recent meta-analysis that included more than 185 studies,^1^ total sperm count has declined by 50% to 60% from 1973 to 2011 in Western countries, in line with a continued decline since the 1940s.^2^ In addition, some investigators have also reported a concomitant secular decline in serum testosterone levels.^3^ Although debate is ongoing about the underlying causes for these declines, there is a growing concern and evidence that environmental exposures such as endocrine-disrupting chemicals and air pollution or behavioral factors such as smoking and alcohol consumption could explain this decline.^4^

Nutritional factors and eating habits are potential behavioral factors contributing to the secular decline in semen quality that have received comparatively little attention. Diet quality has changed dramatically in the last 50 years in Western countries, with a tendency toward higher intakes of total calories, meat, cheese, added fats and sugars, and refined grains.^5^ Many studies have examined the association of isolated nutrients—such as zinc, folate, and antioxidants (positive) and saturated and trans-fats (negative)—with semen quality and other markers of testicular function.^6,7^ More recently, however, interest has shifted to focus on the role of overall diet patterns.^7,8^ Although this literature is still expanding, these studies suggest that adherence to generally healthy diet patterns is associated with better semen quality parameters in North America, Europe, the Middle East, and East Asia.^7,8,9,10,11,12,13,14,15,16^ However, the extent to which these findings may generalize beyond the studied countries is unclear when taking into account local variations in dietary behavior, which are key when designing clinical and public health recommendations.

Therefore, we examined the association between diet patterns reflecting contemporary eating behavior among young men in Denmark and markers of testicular function, including semen quality, testicular volume, and serum reproductive hormone levels. We hypothesized that dietary patterns aligned with dietary recommendations for the prevention of chronic diseases would be associated with better semen quality and other markers of testicular health.

## Methods

### Participants

In Denmark, all men are required to undergo a physical examination at 18 years of age to determine fitness for military service. Starting in 1996, research staff at the University Department of Growth and Reproduction at Rigshospitalet, Copenhagen, Denmark, have approached men presenting for the physical examination in Copenhagen and invited them to participate in an ongoing study aimed at understanding the determinants of male fertility and reproductive potential.^17^ After providing written informed consent, men answered questionnaires about their demographic characteristics, lifestyle, and medical history; provided semen and blood samples; and underwent a physical examination. During the physical examination, weight and height were measured, body mass index (BMI) was calculated as weight in kilograms divided by height in meters squared, and testis size was assessed by ultrasonography.^18^ The local ethical committee approved the study, which follows the Strengthening the Reporting of Observational Studies in Epidemiology (STROBE) reporting guideline.

Participants received Danish 500 kroner (approximately US $85) on completion of the study procedures. Diet assessment with a food frequency questionnaire was introduced to the study in April 2008. Men were eligible for the present analysis if they had answered the food frequency questionnaire (April 1, 2008, to May 31, 2017) and provided a nonazoospermic semen sample. Data were analyzed from July 1, 2017, to January 30, 2019. A total of 2935 men unaware of their fertility status and not using anabolic steroids were included in the analysis, of whom 2798 had complete data on semen quality parameters and testicular volume and 2734 had data on serum reproductive hormone concentrations (eFigure 1 in the Supplement).

### Diet Assessment

The food frequency questionnaire used was a modified version of a previously validated food frequency questionnaire used in the Danish National Birth Cohort^19^ and the Danish Diet, Cancer and Health Studies.^20^ Men reported how often, on average, they consumed specified amounts of 136 food items in the 3 months before enrollment. Portion sizes for individual food items were estimated with the help of photographs, and nutrient intakes were quantified on the basis of the Danish food composition tables.^21^ We grouped individual food items into 40 food groups similar to those used in other studies of Western men^22^ and based on the similarity of nutrient profiles or Danish culinary use (eTable 1 in the Supplement).

### Semen Analysis and Testicular Volume

All men provided a semen sample by masturbation in a room close to the andrology laboratory. The men had been asked to abstain from ejaculation for at least 48 hours before sampling but were still included if abstinence time was shorter. Abstinence time was calculated based on self-reported time of previous ejaculation and time of study sample collection. Semen samples were analyzed in accordance with the current World Health Organization (WHO) guidelines^23^ as previously described.^24^ Briefly, we assessed semen volume, sperm concentration, sperm motility, and sperm morphology.^23,24,25,26,27^ Since 1996, our laboratory has conducted a quality assurance/quality control in comparison with 2 other laboratories.^24,26^ Testicular volume was measured using ultrasonography during the physical examination, and we calculated the mean volume for the right and left testes.

### Reproductive Hormones

Venous blood samples were drawn from the participants in the morning of the day of participation, and serum was isolated and stored at −20 °C. Testosterone concentrations were assessed using a time-resolved fluoroimmunoassay (DELFIA; Wallac) until 2013 and from 2014 onward using an enzyme-linked immunosorbent assay (Access 2; Beckman Coultier Ltd). Free testosterone level was calculated based on the measured serum concentrations of the total testosterone and sex hormone–binding globulin and assumed fixed albumin concentration.^28^ Inhibin B concentrations throughout were determined by a specific 2-sided enzyme-immunometric assay (Inhibin B Gen II; Beckman Coulter Ltd). Concentrations of follicle-stimulating hormone (FSH), luteinizing hormone (LH), sex hormone–binding globulin, and estradiol were measured using a time-resolved immunofluorometric assay (DELFIA; Wallac). From 2014 onward, estradiol concentrations were assessed with a radioimmune assay (Pantex). All hormone levels were analyzed in the same laboratory. The hormone levels were analyzed yearly in batches, including reanalysis of a number of controls from the previous year to ensure comparability over time. We also calculated the ratios of inhibin B to FSH, total testosterone to LH, free testosterone to LH, estradiol to total testosterone, and (estradiol to total testosterone) × 100.

### Statistical Analysis

**Figure 1.**
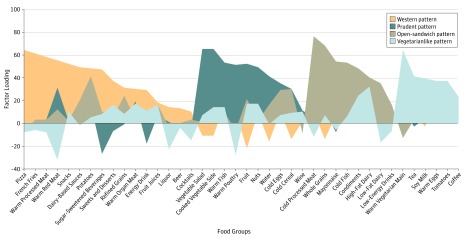
Food Group Factor Loadings for Dietary Patterns Identified From Food-Frequency Questionnaire Data We used principal components analysis as an extraction method in which the factor loading of a food group represents the contribution of that food group to the factor (diet pattern) identified. Positive scores indicate higher consumption of the food group; negative scores, avoidance of the food group.

We used principal component analysis to derive diet patterns based on the 40 predefined food groups (eTable 1 in the Supplement). We used orthogonal transformations (Varimax) to achieve a simpler structure with uncorrelated factors to enhance interpretability. In determining the number of factors to retain, we considered eigenvalues, the scree plot, and the interpretability of the factors based on knowledge of Danish culinary traditions and eating behaviors. The substantive meanings of the rotated factors were considered in conjunction with the above empirical criteria, and the derived factors were labeled based on our interpretation of the data. For every person, we estimated factor scores for each of the 4 retained factors by summing the frequency of consumption multiplied by factor loadings across all food items. Thus, each participant was assigned a score for each of the 4 dietary patterns. Factor loadings for all dietary patterns are presented in Figure 1 with positive loading representing high consumption and negative loading representing avoidance of the corresponding food group in relation to each factor.

We used χ^2^ and Kruskal-Wallis tests to test for differences in demographic and lifestyle characteristics across quintiles of adherence within each pattern. Owing to the skewed outcome variables, we used quantile (median) regression with 95% CIs to estimate the median differences in semen quality, reproductive hormone concentrations, and testicular volume in different quantiles of adherence to each dietary pattern relative to the lowest quintile of adherence.

To address clinical relevance, semen parameters were dichotomized as below or above the WHO 2010 lower reference limits^23^ as well as below the suggested high semen quality reference limits suggested by Damsgaard et al^29^ for high fertility potential using generalized linear models with log link to estimate relative risk (RR). In all regression analyses, we conducted tests for trend across quintiles using the median value in each quintile entered as continuous variable.

Covariates were selected based on prior knowledge using directed acyclic graphs. A small number of missing values were replaced with the median value. Otherwise, missing values were grouped as missing indicators. The final models adjusted for age, BMI, height, smoking, use of marijuana and other recreational drugs, moderate-to-vigorous physical activities (hours per week), history of reproductive diseases, reproductive surgical procedures, sexually transmitted diseases, season and calendar year of the sample, mother’s educational level, and total energy intake. For the semen variables, we further adjusted for abstinence time. In addition, for sperm motility models, we further adjusted for time elapsed between specimen collection and analysis. For the serum reproductive hormone models, we further adjusted for time of the day of the sample collection.

We conducted sensitivity analyses by further adjusting for use of muscle-enhancing products (primarily whey protein and creatine supplements) and without adjusting for BMI (owing to possible mediation). Furthermore, we conducted sensitivity analyses after restricting the data to men who did not report use of muscle-enhancing products and men who never smoked and never used marijuana or other recreational drugs. Results were statistically significant when 2-sided α < .05 for the evaluation of trends across adherence to specific diet patterns and when the 95% CI excluded the null value for pairwise comparisons between categories of adherence. We conducted all statistical analyses using SAS, version 9.4 (SAS Institute Inc).

## Results

The 2935 men included in the analysis had a median age of 19 (interquartile range, 19-20) years, and 2290 (78.0%) had a BMI within the normal range (Table 1 and eTable 2 in the Supplement). We identified 4 dietary patterns: the Western pattern (generally unhealthy), characterized by greater intake of pizza, french fries, processed and red meats, snacks, refined grains, sugar-sweetened beverages, and sweets; the prudent pattern (generally healthy), characterized by greater intake of fish, chicken, vegetables, fruit, and water; the open-sandwich pattern, characterized by greater intake of cold processed meats, whole grains (primarily whole-grain breads), mayonnaise, cold fish, condiments, and dairy; and the vegetarianlike pattern, characterized by intake of vegetables, soy milk, and eggs and avoidance of red meats and chicken (Figure 1 and eTable 3 in the Supplement). Men with higher adherence to the Western pattern were less physically active and vice versa for the prudent and the open-sandwich patterns (Table 1 and eTable 2 in the Supplement). Although no major differences in total carbohydrate intake were noted with increasing adherence to each of the 4 patterns, we found important differences in intakes of fiber, total sugars, and added sugars with increasing adherence to the Western (less fiber and more added sugars), prudent (less added sugars), open-sandwich (more fiber), and vegetarianlike (more added sugars) patterns. Similarly, although no major differences in intakes of total fat were observed, greater adherence to the prudent pattern was associated with higher intake of long-chain ω-3 fatty acids, mostly reflecting differences in fish intake as part of this pattern. Also, major differences in intakes of carotenoids and vitamins C and B reflected differences in fruit and vegetable intake as adherence to the different patterns increased (Table 1 and eTable 2 in the Supplement).

**Table 1.** Demographic Characteristics According to Adherence to Data-Derived Dietary Patterns Among 2935 Danish Young Men (2008-2017)

Characteristic	Dietary Patterns							
	Western		Prudent		Open-Sandwich		Vegetarianlike	
	Quintile 1 (n=587)	Quintile 5 (n=587)	Quintile 1 (n=587)	Quintile 5 (n=587)	Quintile 1 (n=587)	Quintile 5 (n=587)	Quintile 1 (n=587)	Quintile 5 (n=587)
Age, median (IQR), y	19 (19-20)	19 (19-20)	19 (19-20)	19 (19-20)	19 (19-20)	19 (19-20)	19 (19-19)	19 (19-20)
BMI, median (IQR)	22 (21-24)	22 (20-24)	22 (20-24)	22 (21-24)	22 (20-24)	22 (21-24)	23 (21-25)	22 (20-24)
Height, median (IQR), m	1.82 (1.78-1.87)	1.81 (1.77-1.86)	1.81 (1.76-1.86)	1.82 (1.78-1.87)	1.81 (1.76-1.86)	1.83 (1.78-1.88)	1.82 (1.77-1.86)	1.82 (1.77-1.87)
Daily smoking, No. (%)								
Cigarettes	83 (14.1)	233 (39.7)	215 (36.6)	113 (19.3)	180 (30.7)	135 (23.0)	159 (27.1)	153 (26.1)
Marijuana	11 (1.9)	44 (7.5)	40 (6.8)	13 (2.2)	25 (4.3)	18 (3.1)	20 (3.4)	29 (4.9)
Other recreational drug use, No. (%)	45 (7.7)	108 (18.4)	80 (13.6)	59 (10.1)	77 (13.1)	62 (10.6)	72 (12.3)	74 (12.6)
Educational level of the mother >10 y, No. (%)	336 (57.2)	326 (55.5)	293 (49.9)	366 (62.4)	301 (51.3)	334 (56.9)	325 (55.4)	373 (63.5)
Moderate and vigorous physical activities, median (IQR), h/wk	9 (5-14)	8 (4-14)	6 (3-11)	10 (7-16)	6 (3-12)	10 (6-15)	9 (4-15)	8 (4-14)
Fever in last 3 mo, No. (%)	43 (7.3)	44 (7.5)	39 (6.6)	45 (7.7)	61 (10.4)	47 (8.0)	46 (7.8)	40 (6.8)
Self-reported history, No. (%)								
Reproductive disease^a^	107 (18.2)	145 (24.7)	113 (19.3)	122 (20.8)	104 (17.7)	134 (22.8)	127 (21.6)	118 (20.1)
Reproductive surgical procedure^b^	70 (11.9)	72 (12.3)	58 (9.9)	79 (13.5)	62 (10.6)	66 (11.2)	64 (10.9)	53 (9.0)
STD^c^	43 (7.3)	98 (16.7)	87 (14.8)	63 (10.7)	84 (14.3)	61 (10.4)	72 (12.3)	70 (11.9)
Use of muscle-enhancing products in the last 3 mo, No. (%)	193 (32.9)	107 (18.2)	90 (15.3)	236 (40.2)	138 (23.5)	191 (32.5)	211 (35.9)	109 (18.6)
Abstinence time, median (IQR), h	62 (58-85)	62 (56-84)	61 (56-84)	63 (58-85)	62 (58-84)	61 (57-84)	62 (58-84)	62 (56-85)
Sample collected during warm season, No. (%)^d^	204 (34.8)	194 (33.0)	179 (30.5)	204 (34.8)	201 (34.2)	171 (29.1)	187 (31.9)	191 (32.5)
Time of day of sample collection, median (IQR), h	10 (9-11)	10 (10-11)	10 (10-11)	10 (9-11)	10 (9-11)	10 (9-11)	10 (9-11)	10 (10-11)
Time to motility analysis, median (IQR), min	30 (20-45)	30 (25-50)	30 (22-50)	30 (20-45)	30 (23-40)	30 (20-50)	30 (25-45)	30 (25-45)
Total energy intake, median (IQR), kcal/d	1821 (1398-2373)	2303 (1847-3015)	1881 (1416-2482)	2278 (1800-2906)	1637 (1260-2201)	2596 (2021-3257)	2050 (1610-2621)	2118 (1629-2655)
Dietary protein, median (IQR), % of energy^e^	18 (16-21)	17 (15-19)	16 (15-18)	19 (16-21)	18 (16-21)	17 (15-19)	18 (16-21)	17 (15-19)
Total dietary fat, median (IQR), % of energy^e^	31 (28-35)	34 (29-39)	33 (29-38)	33 (29-37)	34 (31-38)	34 (29-38)	35 (30-38)	33 (29-37)
EPA and DHA level, median (IQR), mg/d	392 (243-683)	365 (210-629)	316 (198-562)	506 (302-856)	400 (241-678)	376 (218-720)	357 (211-613)	405 (243-722)
Dietary carbohydrate intake, median (IQR), % of energy^e^	50 (45-54)	48 (42-55)	49 (45-54)	47 (42-52)	46 (41-51)	49 (44-53)	45 (41-51)	49 (44-54)
Total intake, median (IQR), g/d^e^								
Fiber	24 (18-30)	15 (12-19)	18 (13-22)	19 (15-24)	16 (12-20)	20 (15-25)	18 (13-22)	19 (14-24)
Sugar	75 (58-92)	90 (67-123)	92 (70-119)	76 (58-96)	82 (61-102)	78 (60-99)	74 (56-96)	87 (67-107)
Added sugar intake, median (IQR), g/d^e^	23 (13-38)	48 (30-72)	47 (30-68)	27 (16-43)	38 (22-55)	30 (18-50)	28 (16-46)	40 (25-59)
Carotenoid intake, median (IQR), μg/d^e^	2568 (1318-5248)	1788 (960-3776)	1353 (786-2856)	3348 (1746-6093)	2352 (1215-4603)	1985 (1013-3860)	1661 (890-3028)	2879 (1387-5671)
Vitamin B_6_ intake, median (IQR), mg/d^e^	1 (1-2)	1 (1-1)	1 (1-1)	1 (1-2)	1 (1-2)	1 (1-2)	1 (1-2)	1 (1-1)
Folic acid intake, median (IQR), μg/d^e^	271 (230-321)	234 (199-280)	228 (196-269)	271 (229-324)	240 (199-292)	250 (216-293)	232 (200-274)	264 (225-314)
Vitamin B_12_ intake, median (IQR), μg/d^e^	7 (5-9)	6 (5-8)	6 (5-8)	7 (5-9)	6 (5-8)	7 (5-9)	7 (5-8)	6 (5-8)
Vitamin C intake, median (IQR), mg/d^e^	65 (47-87)	63 (49-80)	58 (45-74)	71 (54-93)	67 (51-91)	64 (49-80)	62 (48-81)	68 (50-88)

Abbreviations: BMI, body mass index (calculated as weight in kilograms divided by height in meters squared); DHA, docosahexaenoic acid; EPA, eicosapentaenoic acid; IQR, interquartile range; STD, sexually transmitted disease.

^a^ Includes self-reported history of varicocele, cryptorchidism, testicular mumps, inguinal hernia, testicular injury (hit, kicked, or otherwise injured so it caused swelling of the scrotum), hydrocele, testicular torsion, hypospadias, epididymo-orchitis, cystitis, or prostatitis.

^b^ Includes self-reported history of surgery for inguinal hernia, varicocele, hydrocele, testicular torsion, hypospadias, testicular cancer, phimosis, testicular biopsy, vasectomy, refertilization, and other.

^c^ Includes self-reported history of gonorrhea, chlamydia, and other STDs.

^d^ Indicates April through September.

^e^ Adjusted for energy intake using the residual method making nutrient intake independent from energy intake.

The median total sperm count was 140 (95% CI, 133-146) million and median total testosterone concentration was 524 (95% CI, 518-530) ng/dL (to convert to nanomoles per liter, multiply by 0.0347) (eTable 4 in the Supplement). Four hundred sixty-six men (16.7%) had sperm concentrations below the WHO lower reference limits, whereas 1539 (55.0%) had sperm concentrations above the higher reference limit of 40 million/mL set by Damsgaard et al.^29^ Among men with the highest adherence to each of the dietary patterns, the highest median total sperm count was observed in the prudent pattern (167 [95% CI, 146-183] million), followed by the vegetarianlike pattern (151 [95% CI, 134-168] million) and the open-sandwich pattern (146 [95% CI, 131-163] million). Men with the highest adherence to the Western pattern had the lowest median total sperm count (122 [95% CI, 109-138] million) (eFigure 2 in the Supplement). Analyses comparing differences across patterns showed that, relative to men with the greatest adherence to the Western pattern, men with the greatest adherence to the vegetarianlike (median, 32 [95% CI, 8-56] million) and the prudent (median, 68 [95% CI, 43-93] million) patterns had significantly higher total sperm counts. A similar pattern was observed for all semen parameters and testicular volume. Men in the highest vs lowest quintile of the Western pattern had a lower median ratio of inhibin B to FSH (62 [95% CI, 57-67] vs 71 [95% CI, 61-77]), a higher median ratio of free testosterone to LH (143 [95% CI, 136-148] vs 127 [95% CI, 122-133]), and a lower median ratio of free testosterone to estradiol (17 [95% CI, 17-18] vs 19 [95% CI, 18-19]) (eTable 5 in the Supplement).

**Figure 2.**
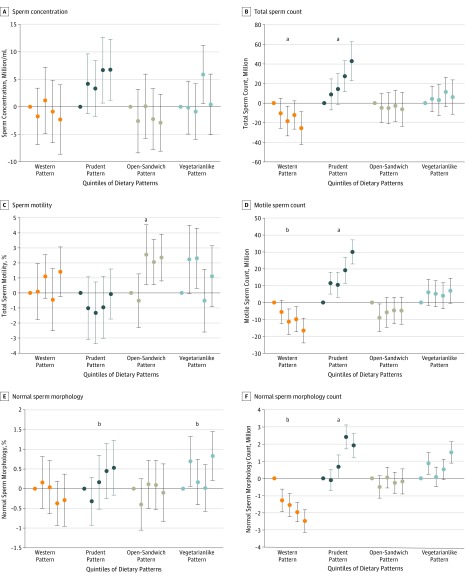
Adjusted Median Differences in Semen Quality Parameters According to Quintiles of Adherence to Data-Derived Dietary Patterns Models were adjusted for age, body mass index, height, smoking, use of marijuana and other recreational drugs, moderate-to-vigorous physical activities (hours per week), history of reproductive diseases, reproductive surgical procedures, sexually transmitted diseases, season and calendar year of the sample, mother’s educational level, total energy intake, and abstinence time. Sperm motility models were further adjusted for time elapsed between specimen collection and analysis. Error bars indicate 95% CIs. Tests for trend were conducted across quintiles using the median value in each quintile of the diet patterns as a continuous variable in the regression models, and the *P* value was based on the Wald test. ^a^*P* < .01. ^b^*P* < .05.

In the multivariable analysis, the median total sperm count for men in the highest quintile of adherence to the Western pattern was 26 (95% CI, –42 to –9) million lower than that of men in the lowest quintile of adherence to this pattern. Conversely, the median total sperm count of men in the highest quintile of adherence to the prudent pattern was 43 (95% CI, 23-63) million higher than that of men in the lowest quintile (Figure 2 and eTable 6 in the Supplement). Similar patterns were observed with all sperm parameters and testicular volume. In addition, the median percentage of motile spermatozoa for men in the highest quintile of adherence to the open-sandwich pattern was 2.3% (95% CI, 0.8%-3.9%) higher than that of men in the lowest quintile, and the median percentage of morphologically normal sperm for men in the highest quintile of adherence to the vegetarianlike pattern was 0.8% (95% CI, 0.2%-1.4%) higher than that of men in the lowest quintile (eTable 6 in the Supplement).

Higher adherence to the Western pattern was associated with a lower inhibin B level (median, −10 [95% CI, −18 to −3] pg/mL) and a lower ratio of inhibin B to FSH (median, −12 [95% CI, −20 to −3]), whereas the latter was nonstatistically significantly lower with adherence to the other patterns. Higher adherence to the Western pattern was associated with a higher ratio of free testosterone to LH (median, 10 [95% CI, 2-19]), whereas the open-sandwich pattern was associated with a lower ratio of free testosterone to LH (median, −8 [95% CI, −16 to −1]). Higher adherence to the Western pattern was associated with lower ratio of estradiol to free testosterone (median, −1.2 [95% CI, −2.0 to −0.3]) (Table 2).

**Table 2.** Serum Reproductive Hormone Concentrations and Testicular Volume According to Adherence to Data-Derived Dietary Patterns^a^

Dietary Pattern	Reproductive Parameter, Adjusted Median Difference (95% CI)												
	Testicular Volume, mL^b^	Total T Level, ng/dL	Free T Level, pmol/L	E_2_ Level, pg/mL	Inhibin B Level, pg/mL	LH Level, mIU/mL	FSH Level, mIU/mL	SHBG Level, nmol/L	Ratio of Inhibin B to FSH	Ratio of Total T:LH	Ratio of Free T:LH	Ratio of E2:Total T	(Ratio of E2:Free T) × 100
Western													
Quintile 1	1 [Reference]	1 [Reference]	1 [Reference]	1 [Reference]	1 [Reference]	1 [Reference]	1 [Reference]	1 [Reference]	1 [Reference]	1 [Reference]	1 [Reference]	1 [Reference]	1 [Reference]
Quintile 2	−0.7 (−1.1 to −0.2)	8.6 (−11.5 to 25.9)	12 (−5 to 28)	1 (−3 to 4)	−4 (−11 to 3)	0.1 (0.0 to 0.3)	0.1 (−0.1 to 0.4)	0.5 (−0.9 to 1.9)	−5 (−13 to 2)	−0.1 (−0.4 to 0.2)	3 (−5 to 10)	−0.1 (−0.2 to 0.1)	−0.6 (−1.4 to 0.3)
Quintile 3	−0.4 (−0.9 to 0.1)	14.4 (−11.5 to 37.5)	25 (6 to 43)	2 (−2 to 5)	5 (−2 to 13)	0.01 (−0.2 to 0.2)	0.1 (−0.1 to 0.3)	−0.4 (−2.0 to 1.1)	−1 (−9 to 7)	0.1 (−0.3 to 0.4)	6 (−2 to 14)	−0.02 (−0.2 to 0.2)	−0.6 (−1.5 to 0.3)
Quintile 4	−0.3 (−0.9 to 0.2)	25.9 (5.8 to 46.1)	31 (12 to 50)	1 (−2 to 5)	−6 (−13 to 2)	0.2 (0.0 to 0.4)	0.3 (0.0 to 0.5)	−0.01 (−1.6 to 1.6)	−11 (−19 to −3)	0 (−0.3 to 0.4)	5 (−4 to 14)	0.01 (−0.2 to 0.2)	−0.9 (−1.7 to 0.03)
Quintile 5	−0.8 (−1.3 to −0.2)	25.9 (2.9 to 49.0)	49 (29 to 69)	4 (1 to 7)	−10 (−18 to −3)	0.2 (0.1 to 0.4)	0.2 (0.0 to 0.5)	−0.8 (−2.5 to 0.8)	−12 (−20 to −3)	0.1 (−0.3 to 0.5)	10 (2 to 19)	0.01 (−0.2 to 0.2)	−1.2 (−2.0 to −0.3)
*P* value for trend	.13	.007	<.001	.08	.006	.16	.10	.21	.003	.55	.006	.73	.009
Prudent													
Quintile 1	1 [Reference]	1 [Reference]	1 [Reference]	1 [Reference]	1 [Reference]	1 [Reference]	1 [Reference]	1 [Reference]	1 [Reference]	1 [Reference]	1 [Reference]	1 [Reference]	1 [Reference]
Quintile 2	0.3 (−0.1 to 0.7)	−11.5 (−28.8 to 8.6)	4 (−16 to 23)	−2 (−6 to 1)	2 (−5 to 8)	−0.03 (−0.3 to 0.2)	0.1 (−0.1 to 0.3)	0.4 (−1.0 to 1.9)	−5 (−12 to 2)	−0.01 (−0.3 to 0.3)	1 (−7 to 9)	−0.03 (−0.2 to 0.2)	−0.4 (−1.1 to 0.4)
Quintile 3	0.5 (0.1 to 1.0)	0.6 (−20.2 to 23.1)	−3 (−22 to 17)	−4 (−6 to −1)	10 (3 to 18)	−0.2 (−0.4 to 0.0)	−0.2 (−0.4 to 0.0)	0.7 (−0.7 to 2.2)	6 (−1 to 13)	0.3 (−0.03 to 0.7)	6 (−3 to 14)	−0.1 (−0.3 to 0.02)	−0.5 (−1.2 to 0.3)
Quintile 4	0.1 (−0.4 to 0.5)	−8.6 (−28.8 to 14.4)	−0.1 (−19 to 19)	−5 (−8 to −2)	0.1 (−8 to 8)	−0.1 (−0.3 to 0.1)	0.1 (−0.1 to 0.3)	0.3 (−1.0 to 1.7)	−6 (−12 to 1)	0.1 (−0.2 to 0.5)	0.3 (−8 to 8)	−0.1 (−0.3 to 0.04)	−0.3 (−1.1 to 0.6)
Quintile 5	0.2 (−0.3 to 0.7)	−5.8 (−28.8 to 14.4)	−10 (−29 to 9)	−5 (−8 to −1)	2 (−5 to 9)	−0.1 (−0.3 to 0.1)	0.1 (−0.1 to 0.4)	1.9 (0.3 to 3.4)	−5 (−11 to 0)	0.2 (−0.2 to 0.6)	4 (−3 to 12)	−0.1 (−0.3 to 0.03)	−0.2 (−1.1 to 0.6)
*P* value for trend	.56	.76	.20	.001	.81	.57	.44	.04	.18	.62	.44	.19	.71
Open sandwich													
Quintile 1	1 [Reference]	1 [Reference]	1 [Reference]	1 [Reference]	1 [Reference]	1 [Reference]	1 [Reference]	1 [Reference]	1 [Reference]	1 [Reference]	1 [Reference]	1 [Reference]	1 [Reference]
Quintile 2	−0.6 (−1.0 to −0.2)	5.8 (−17.3 to 25.9)	12 (−7 to 30)	2 (−2 to 5)	−1 (−9 to 7)	0.1 (−0.1 to 0.3)	0.1 (−0.1 to 0.3)	0.3 (−1.1 to 1.6)	−1 (−7 to 5)	0.1 (−0.3 to 0.4)	1 (−8 to 9)	0 (−0.2 to 0.2)	0.2 (−0.5 to 1.0)
Quintile 3	−0.4 (−0.8 to 0.0)	−5.8 (−25.9 to 14.4)	8 (−10 to 26)	0.4 (−3 to 4)	−1 (−10 to 7)	0.1 (−0.1 to 0.3)	0.04 (−0.2 to 0.2)	0.4 (−1.1 to 1.8)	−1 (−7 to 6)	−0.2 (−0.6 to 0.2)	−7 (−14 to 1)	0.2 (0.0 to 0.4)	0.2 (−0.6 to 1.0)
Quintile 4	−0.3 (−0.8 to 0.1)	14.4 (−11.5 to 37.5)	−2 (−23 to 18)	4 (1 to 8)	0.03 (−8 to 8)	0.3 (0.1 to 0.5)	0.1 (−0.1 to 0.3)	1.0 (−0.6 to 2.6)	1 (−7 to 8)	−0.2 (−0.6 to 0.1)	−11 (−19 to −3)	0.1 (−0.1 to 0.3)	0.7 (−0.2 to 1.6)
Quintile 5	−0.5 (−1.0 to −0.1)	14.4 (−8.6 to 37.5)	4 (−16 to 24)	4 (0 to 8)	−8 (−16 to 0)	0.2 (0.0 to 0.4)	0.1 (−0.1 to 0.4)	1.3 (−0.3 to 2.9)	−7 (−14 to 0)	−0.3 (−0.7 to 0.1)	−8 (−16 to −1)	−0.1 (−0.2 to 0.1)	0.5 (−0.2 to 1.3)
*P* value for trend	.16	.27	.40	.04	.03	.07	.33	.06	.06	.03	.03	.28	.23
Vegetarianlike													
Quintile 1	1 [Reference]	1 [Reference]	1 [Reference]	1 [Reference]	1 [Reference]	1 [Reference]	1 [Reference]	1 [Reference]	1 [Reference]	1 [Reference]	1 [Reference]	1 [Reference]	1 [Reference]
Quintile 2	0 (−0.4 to 0.4)	17.3 (−2.9 to 40.3)	7 (−9 to 24)	3 (−1 to 6)	−4 (−12 to 3)	−0.001 (−0.2 to 0.2)	0.1 (−0.1 to 0.3)	0.5 (−0.8 to 1.8)	−9 (−16 to −3)	0.4 (0 to 0.7)	8 (0 to 16)	−0.1 (−0.3 to 0.1)	−0.2 (−1.1 to 0.7)
Quintile 3	−0.3 (−0.7 to 0.1)	−0.3 (−20.2 to 17.3)	4 (−13 to 20)	2 (−2 to 5)	−3 (−11 to 5)	−0.02 (−0.2 to 0.2)	0.3 (0.0 to 0.5)	0.3 (−1.0 to 1.7)	−10 (−17 to −3)	−0.1 (−0.5 to 0.2)	−1 (−7 to 6)	0 (−0.2 to 0.2)	0.2 (−0.6 to 1.1)
Quintile 4	0.0 (−0.5 to 0.4)	20.2 (−0.1 to 43.2)	5 (−11 to 22)	2 (−2 to 5)	−4 (−12 to 4)	0.1 (−0.1 to 0.3)	0.1 (−0.1 to 0.3)	1.4 (0.03 to 2.8)	−6 (−14 to 1)	0.3 (−0.1 to 0.6)	3 (−4 to 10)	−0.2 (−0.4 to 0.003)	−0.4 (−1.2 to 0.4)
Quintile 5	0.2 (−0.3 to 0.6)	20.2 (−0.1 to 40.3)	10 (−7 to 28)	−1 (−4 to 3)	−9 (−17 to −2)	−0.1 (−0.3 to 0.1)	0.1 (−0.2 to 0.3)	1.5 (0.1 to 2.9)	−9 (−15 to −2)	0.3 (0 to 0.7)	7 (−2 to 15)	−0.2 (−0.3 to 0.002)	−0.8 (−1.5 to 0.02)
*P* value for trend	.31	.08	.23	.24	.009	.48	.87	.03	.10	.11	.34	.07	.08

Abbreviations: E_2_, estradiol; FSH, follicle-stimulating hormone; LH, luteinizing hormone; SHBG, sex hormone–binding globulin; T, testosterone.

SI conversion factors: To convert E_2_ to picomoles per liter, multiply by 3.671; FSH and LH to international units per liter, multiply by 1.0; total T to nanomoles per liter, multiply by 0.0347.

^a^ Models were adjusted for age, body mass index, height, smoking, use of marijuana and other recreational drugs, moderate-to-vigorous physical activities (hours per week), history of reproductive diseases, reproductive surgical procedures, sexually transmitted diseases, season and calendar year of the sample, mother’s educational level, total energy intake, and time of the day of the sample collection (except for testicular volume). Tests for trend were conducted across quintiles using the median value in each quintile as a continuous variable in the regression models; *P* value was based on the Wald test.

^b^ Measured using ultrasonography and reported as mean (95% CI) of both testicles.

**Figure 3.**
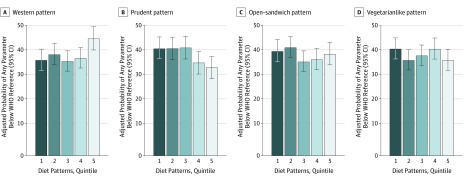
Adjusted Probabilities of Having Any Semen Parameter Below the World Health Organization (WHO) Lower Reference Limits According to Quintiles of Adherence to Data-Derived Dietary Patterns The WHO lower reference limits for semen parameters are less than 1.5 mL for semen volume, less than 15 million/mL for sperm concentration, less than 39 million for total sperm count, less than 40% for motile spermatozoa, less than 32% for progressively motile spermatozoa, and less than 4% for normal sperm morphology. Models were adjusted for age, body mass index, height, smoking, use of marijuana and other recreational drugs, moderate-to-vigorous physical activities (hours per week), history of reproductive diseases, reproductive surgical procedures, sexually transmitted diseases, season and calendar year of the sample, mother’s educational level, total energy intake, and abstinence time. Sperm motility models were further adjusted for time elapsed between specimen collection and analysis. For the Western pattern, *P* = .006 for trend; for the prudent pattern, *P* = .004 for trend; for the open-sandwich pattern, *P* = .54 for trend; and for the vegetarianlike pattern, *P* = .39 for trend.

We then evaluated the association between diet patterns and having any semen parameter below the WHO lower reference limits^23^ and the higher semen quality limits of Damsgaard et al.^29^ In this analysis, high adherence to the Western diet pattern was associated with a higher estimated probability of having at least 1 semen parameter below the WHO reference limits. Men in the highest quintile of adherence to the Western pattern were more likely to have a semen parameter below the WHO limits (RR, 1.2 [95% CI, 1.1-1.4]) than men in the lowest quintile of adherence (eTable 7 in the Supplement). This association was mainly driven by differences in the probability of having samples with total sperm count, concentration, and volume below the reference limits. Conversely, men with greater adherence to the prudent pattern had a lower probability of having sperm parameters below the WHO reference limits (RR, 0.8 [95% CI, 0.7-1.0]) (Figure 3 and eTable 7 in the Supplement). We observed consistent results with the limits by Damsgaard et al^29^ (eTable 8 in the Supplement). Similar associations were observed in sensitivity analyses in which we further adjusted for use of muscle-enhancing supplements, fit models without adjustment for BMI, and restricted analyses to men who did not report use of muscle-enhancing products and who never smoked or used marijuana or other recreational drugs (eTables 9-16 in the Supplement).

## Discussion

We studied a group of young men who were unaware of their fertility status and found that men with generally healthier diets had better testicular function. Specifically, greater adherence to a Western pattern was associated with lower semen quality as well as lower serum inhibin B concentrations and ratios of inhibin B to FSH, suggesting reduced spermatogenesis.^30,31^ In contrast, adherence to a prudent pattern was associated with higher semen quality. Higher adherence to an open-sandwich pattern (a traditional Danish pattern with whole grain bread) was associated with a higher count of motile spermatozoa, whereas adherence to a vegetarianlike pattern was associated with a higher percentage of morphologically normal spermatozoa. To our knowledge, this is the largest study to date examining the association between diet patterns and markers of testicular function.

Our results are consistent with our hypothesis that adherence to healthy diet patterns is associated with better testicular health, as reflected in semen quality parameters and reproductive hormone concentrations. Healthier dietary patterns, including local variations such as the Mediterranean diet in studies conducted in Spain^16^ and Greece,^32^ have been consistently associated with better semen quality. In recent reviews,^7,9,10^ dietary patterns favoring intakes of seafood, poultry, whole grains, legumes, skim milk, fruits, and vegetables have been consistently associated with better semen parameters in studies in North America, Europe, the Middle East, and Asia.^7,8,11,12,13,14,15,16,32,33^ A study in the Netherlands found that men who adhered to a healthy diet pattern had higher sperm concentration, total sperm count, and motile spermatozoa.^34^ In another study in Poland, the prudent dietary pattern was associated with higher sperm concentrations.^15^ Associations between unhealthy dietary patterns and lower semen quality have been less consistent, however. Our findings suggest that local variations to generally healthy diets beyond the Mediterranean diet pattern may also offer benefits that could be missed in studies focused on more traditional diet scores. For example, the only diet pattern associated with sperm motility in our study was the open-sandwich pattern, which reflects a Nordic culinary tradition characterized by consumption of whole-grain bread, cured or smoked fish, and high-fat dairy products. However, we cannot exclude the role of chance of this isolated association with sperm motility. The differences in semen quality observed may reflect the differences in nutritional profiles observed across levels of adherence to the 4 patterns identified. Previous work focused on intake of specific nutrients suggests that the associations observed herein could be explained by differences in intake of long-chain ω-3 fatty acids,^35,36,37^ carotenoids, vitamins C and B,^38,39^ and possibly differences in carbohydrate quality, including intake of added sugars.^40,41,42^

The interpretation of the hormone findings is less straightforward. Men with the highest adherence to the Western pattern had higher testosterone levels, assessed as total and free testosterone, compared with men with less adherence. At the same time, they also had the highest estradiol concentration but unchanged LH levels. Thus, it appears that adherence to the Western pattern may lead to an increased aromatization of testosterone to estradiol. We speculate that this change has resulted in increased negative feedback at the hypothalamic level. If this is correct, it might also explain why FSH is not sufficiently increased as a compensation for the lower inhibin B levels. Thus, we can speculate that adherence to the Western pattern at least partly leads to some degree of reduced hypothalamic activity that in turn leads to a reduction in spermatogenesis. In addition to this speculation, the lower ratio of inhibin B to FSH itself points toward a direct (primary) adverse effect on the testicles. If the associations reflect causation, adherence to a Western diet may lead to combined primary and secondary endocrine reduction of spermatogenesis. However, we cannot clarify that association any further. Similarly, we cannot clarify the biological significance of the slightly lower ratio of testosterone to LH for men with adherence to the open-sandwich pattern.

Fewer studies have examined the association of dietary patterns with reproductive hormones and testicular size. Unlike our results, Jurewicz and colleagues^15^ reported that adherence to the prudent pattern was associated with higher testosterone concentrations, whereas a Western diet was not associated with testosterone levels among 336 men in Poland. However, their study included only men who attended a fertility clinic with semen parameters higher than the 1999 reference limits according to the WHO or with oligozoospermia, which could have introduced selection bias.^15^ In addition, there was no observed association of the Western and Mediterranean patterns with testicular volume or reproductive hormones in 215 men in Spain.^16^ The lower inhibin B level and ratio of inhibin B to FSH associated with higher adherence to the Western pattern is consistent with the lower semen parameters, thus indicating a direct reduction of the testicular capacity for spermatogenesis. Thus, together with the speculations about the significance of altered testosterone and estradiol concentrations, the reduction of semen quality in men with highest adherence to the Western pattern may reflect a combined reduction of hypothalamic-pituitary function as well as a direct negative effect on the testicles.^31,43^

### Strengths and Limitations

Strengths of the study include the large sample size, affording high statistical power and precision. Most importantly, because these men were not aware of their fertility status, the generalizability of the results to the general population of young men regarding testicular function is increased and selection bias is unlikely. In addition, participants not included in this analysis had comparable demographics to men in this analysis^17,24^ (eTable 17 in the Supplement) and comparable concentrations of the reproductive hormones.^44^ Finally, the use of dietary pattern allows for easier translation of these results into clinical or public health recommendations.^22^

Our study has some limitations, including the potential misclassification of self-reported diet and reproductive parameters based on a single measurement. However, previous studies^45,46^ have shown that a single semen sample may suffice for studies aimed at identifying mean differences in semen quality between men. Because this was a cross-sectional study, it limits our ability to determine causality; however, dietary habits tend to be quite stable over time.^47,48^ We acknowledge that multiple comparison may be an issue, because we compared 4 main exposures in association with multiple related outcomes. However, even using a Bonferroni-adjusted *P* = .003, our main conclusions remain unchanged. In addition, we cannot exclude residual confounding, although we did adjust for many potential confounders.

## Conclusions

Our findings support the evidence that adhering to generally healthy diet patterns is associated with better semen quality and more favorable markers of testicular function. Because diet is modifiable, these results suggest the possibility of using dietary intervention as a potential approach to improving testicular function in men of reproductive age.

## Notes

Supplement. **eFigure 1.** Flowchart for the Sample Size of the Danish Men Eligible for the Analysis **eFigure 2. **Unadjusted Medians in Semen Quality Parameters and Testicular Volume According to Adherence to Data-Derived Dietary Patterns **eTable 1. **Food Groupings Used in the Dietary Pattern Analyses **eTable 2. **Total Demographic Characteristics Among 2935 Danish Young Men (2008-2017) and According to Adherence to Data-Derived Dietary Patterns **eTable 3. **Food Group Loadings for 2 Dietary Patterns Identified From Food-Frequency Questionnaire **eTable 4. **Summary of the Distribution of Reproductive Parameters **eTable 5. **Unadjusted Medians in Serum Concentrations of Reproductive Hormones on the Scale for the Individual Hormones as Stated in the Table According to Adherence to Data-Derived Dietary Patterns **eTable 6. **Adjusted Median Differences in Semen Quality Parameters According to Adherence to Data-Derived Dietary Patterns **eTable 7. **Adjusted Probabilities of Semen Quality Parameters Below the WHO Lower Reference Limits According to Adherence to Data-Derived Dietary Patterns **eTable 8. **Adjusted Probabilities of Semen Quality Parameters Below the Damsgaard High Semen Quality Suggested Reference Limits According to Adherence to Data-Derived Dietary Patterns **eTable 9. **Adjusted Median Differences in Semen Quality Parameters and Testicular Volume According to Adherence to Data-Derived Dietary Patterns, Additionally Adjusted for Use of Muscle-Enhancing Supplements **eTable 10.** Adjusted Median Differences in Serum Reproductive Hormone Concentrations on the Scale for the Individual Hormones as Stated in the Table According to Adherence to Data-Derived Dietary Patterns, Additionally Adjusted for Use of Muscle-Enhancing Supplements **eTable 11. **Adjusted Median Differences in Semen Quality Parameters and Testicular Volume According to Adherence to Data-Derived Dietary Patterns, Without Adjustment for Body Mass Index **eTable 12. **Adjusted Median Differences in Serum Reproductive Hormone Concentrations on the Scale for the Individual Hormones as Stated in the Table According to Adherence to Data-Derived Dietary Patterns, Without Adjustment for Body Mass Index **eTable 13. **Adjusted Median Differences in Semen Quality Parameters and Testicular Volume According to Adherence to Data-Derived Dietary Patterns Among 2169 Men Who Did Not Report Muscle-Enhancing Supplements **eTable 14. **Adjusted Median Differences in Serum Reproductive Hormone Concentrations on the Scale for the Individual Hormones as Stated in the Table According to Adherence to Data-Derived Dietary Patterns Among 2169 Men Who Did Not Report Muscle-Enhancing Supplements **eTable 15. **Adjusted Median Differences in Semen Quality Parameters and Testicular Volume According to Adherence to Data-Derived Dietary Patterns Among 1175 Men Who Were Never Smokers, Never Marijuana Users, and Nonusers of Other Recreational Drugs **eTable 16. **Adjusted Median Differences in Serum Reproductive Hormone Concentrations on the Scale for the Individual Hormones as Stated in the Table According to Adherence to Data-Derived Dietary Patterns, Among 1175 Men Who Were Never Smokers, Never Marijuana Users, and Nonusers Of Other Recreational Drugs **eTable 17. **Demographic Characteristics of Men Who Were and Were Not Included in the Analysis
