# Supplementary material for: Association of Dietary Patterns With Testicular Function in Young Danish Men
Source: JAMA Netw Open. 2020 Feb 21;3(2):e1921610. doi: 10.1001/jamanetworkopen.2019.21610 (PMC7043196; doi:10.1001/jamanetworkopen.2019.21610)
Supplement: Supplement. — eFigure 1. Flowchart for the Sample Size of the Danish Men Eligible for the Analysis eFigure 2. Unadjusted Medians in Semen Quality Parameters and Testicular Volume According to Adherence to Data-Derived Dietary Patterns eTable 1. Food Groupings Used in the Dietary Pattern Analyses eTable 2. Total Demographic Characteristics Among 2935 Danish Young Men (2008-2017) and According to Adherence to Data-Derived Dietary Patterns eTable 3. Food Group Loadings for 2 Dietary Patterns Identified From Food-Frequency Questionnaire eTable 4. Summary of the Distribution of Reproductive Parameters eTable 5. Unadjusted Medians in Serum Concentrations of Reproductive Hormones on the Scale for the Individual Hormones as Stated in the Table According to Adherence to Data-Derived Dietary Patterns eTable 6. Adjusted Median Differences in Semen Quality Parameters According to Adherence to Data-Derived Dietary Patterns eTable 7. Adjusted Probabilities of Semen Quality Parameters Below the WHO Lower Reference Limits According to Adherence to Data-Derived Dietary Patterns eTable 8. Adjusted Probabilities of Semen Quality Parameters Below the Damsgaard High Semen Quality Suggested Reference Limits According to Adherence to Data-Derived Dietary Patterns eTable 9. Adjusted Median Differences in Semen Quality Parameters and Testicular Volume According to Adherence to Data-Derived Dietary Patterns, Additionally Adjusted for Use of Muscle-Enhancing Supplements eTable 10. Adjusted Median Differences in Serum Reproductive Hormone Concentrations on the Scale for the Individual Hormones as Stated in the Table According to Adherence to Data-Derived Dietary Patterns, Additionally Adjusted for Use of Muscle-Enhancing Supplements eTable 11. Adjusted Median Differences in Semen Quality Parameters and Testicular Volume According to Adherence to Data-Derived Dietary Patterns, Without Adjustment for Body Mass Index eTable 12. Adjusted Median Differences in Serum Reproductive Hormone Concentrations on the Sca [file jamanetwopen-3-e1921610-s001.pdf]

Supplementary Online Content

Nassan FL, Jensen TK, Priskorn L, Halldorsson TI, Chavarro JE, Jørgensen N. Association of dietary patterns with testicular function in young Danish men. *JAMA Netw Open*. 2020;3(2):e1921610. doi:10.1001/jamanetworkopen.2019.21610

eFigure 1. Flowchart for the Sample Size of the Danish Men Eligible for the Analysis

eFigure 2. Unadjusted Medians in Semen Quality Parameters and Testicular Volume According to Adherence to Data-Derived Dietary Patterns

eTable 1. Food Groupings Used in the Dietary Pattern Analyses

eTable 2. Total Demographic Characteristics Among 2935 Danish Young Men (2008-2017) and According to Adherence to Data-Derived Dietary Patterns

eTable 3. Food Group Loadings for 2 Dietary Patterns Identified From Food-Frequency Questionnaire

eTable 4. Summary of the Distribution of Reproductive Parameters

eTable 5. Unadjusted Medians in Serum Concentrations of Reproductive Hormones on the Scale for the Individual Hormones as Stated in the Table According to Adherence to Data-Derived Dietary Patterns

eTable 6. Adjusted Median Differences in Semen Quality Parameters According to Adherence to Data-Derived Dietary Patterns

eTable 7. Adjusted Probabilities of Semen Quality Parameters Below the WHO Lower Reference Limits According to Adherence to Data-Derived Dietary Patterns

eTable 8. Adjusted Probabilities of Semen Quality Parameters Below the Damsgaard High Semen Quality Suggested Reference Limits According to Adherence to Data-Derived Dietary Patterns

eTable 9. Adjusted Median Differences in Semen Quality Parameters and Testicular Volume According to Adherence to Data-Derived Dietary Patterns, Additionally Adjusted for Use of Muscle-Enhancing Supplements

eTable 10. Adjusted Median Differences in Serum Reproductive Hormone Concentrations on the Scale for the Individual Hormones as Stated in the Table According to Adherence to Data-Derived Dietary Patterns, Additionally Adjusted for Use of Muscle-Enhancing Supplements

eTable 11. Adjusted Median Differences in Semen Quality Parameters and Testicular Volume According to Adherence to Data-Derived Dietary Patterns, Without Adjustment for Body Mass Index

eTable 12. Adjusted Median Differences in Serum Reproductive Hormone Concentrations on the Scale for the Individual Hormones as Stated in the Table According to Adherence to Data-Derived Dietary Patterns, Without Adjustment for Body Mass Index

©2020 Nassan FL et al. *JAMA Network Open*.

eTable 13. Adjusted Median Differences in Semen Quality Parameters and Testicular Volume According to Adherence to Data-Derived Dietary Patterns Among 2169 Men Who Did Not Report Muscle-Enhancing Supplements

eTable 14. Adjusted Median Differences in Serum Reproductive Hormone Concentrations on the Scale for the Individual Hormones as Stated in the Table According to Adherence to Data-Derived Dietary Patterns Among 2169 Men Who Did Not Report Muscle-Enhancing Supplements

eTable 15. Adjusted Median Differences in Semen Quality Parameters and Testicular Volume According to Adherence to Data-Derived Dietary Patterns Among 1175 Men Who Were Never Smokers, Never Marijuana Users, and Nonusers of Other Recreational Drugs

eTable 16. Adjusted Median Differences in Serum Reproductive Hormone Concentrations on the Scale for the Individual Hormones as Stated in the Table According to Adherence to Data-Derived Dietary Patterns, Among 1175 Men Who Were Never Smokers, Never Marijuana Users, and Nonusers Of Other Recreational Drugs

eTable 17. Demographic Characteristics of Men Who Were and Were Not Included in the Analysis

This supplementary material has been provided by the authors to give readers additional information about their work.

©2020 Nassan FL et al. *JAMA Network Open*.

eFigure 1. Flowchart for the Sample Size of the Danish Men Eligible for the Analysis

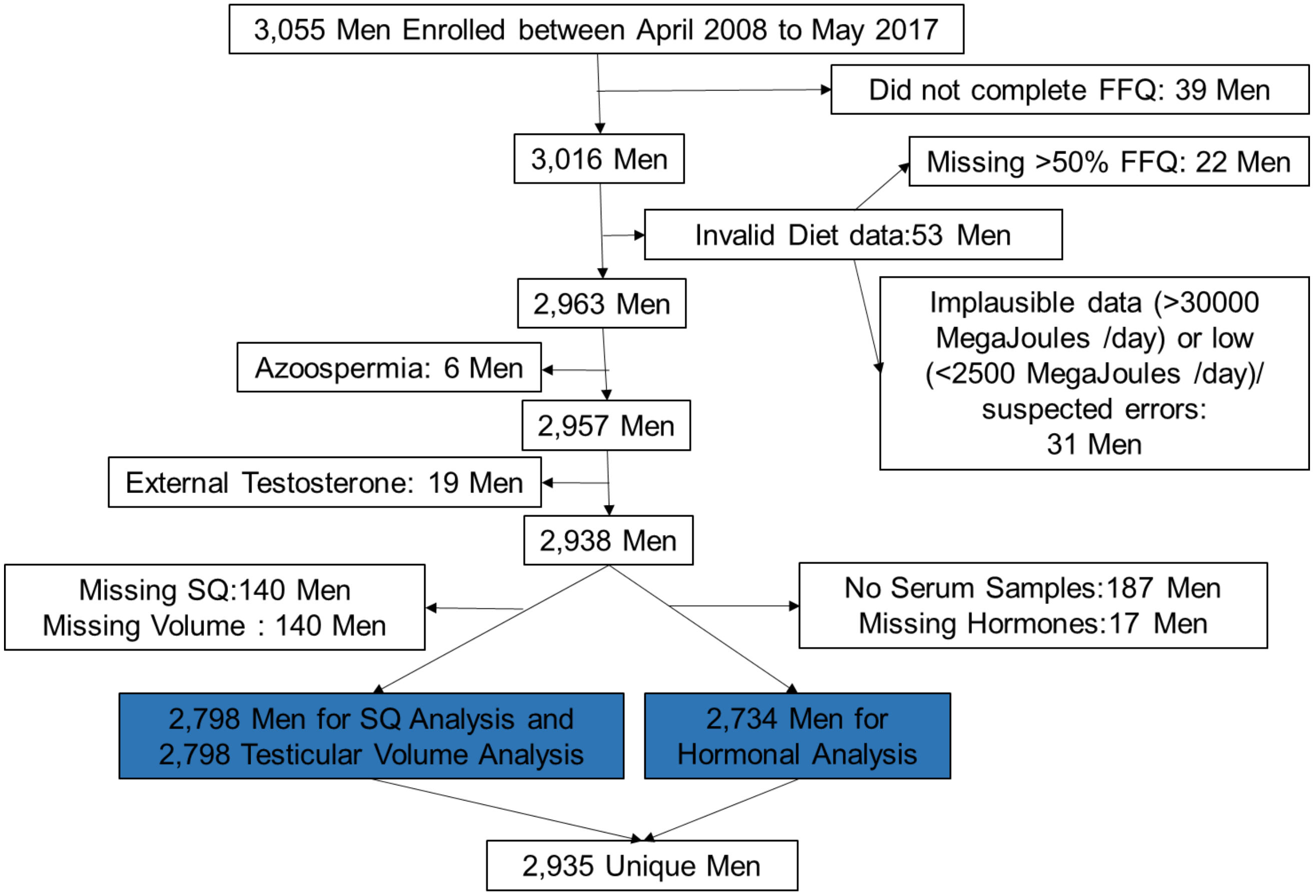

©2020 Nassan FL et al. *JAMA Network Open*.

eFigure 2. Unadjusted Medians in Semen Quality Parameters and Testicular Volume According to Adherence to Data-Derived Dietary Patterns

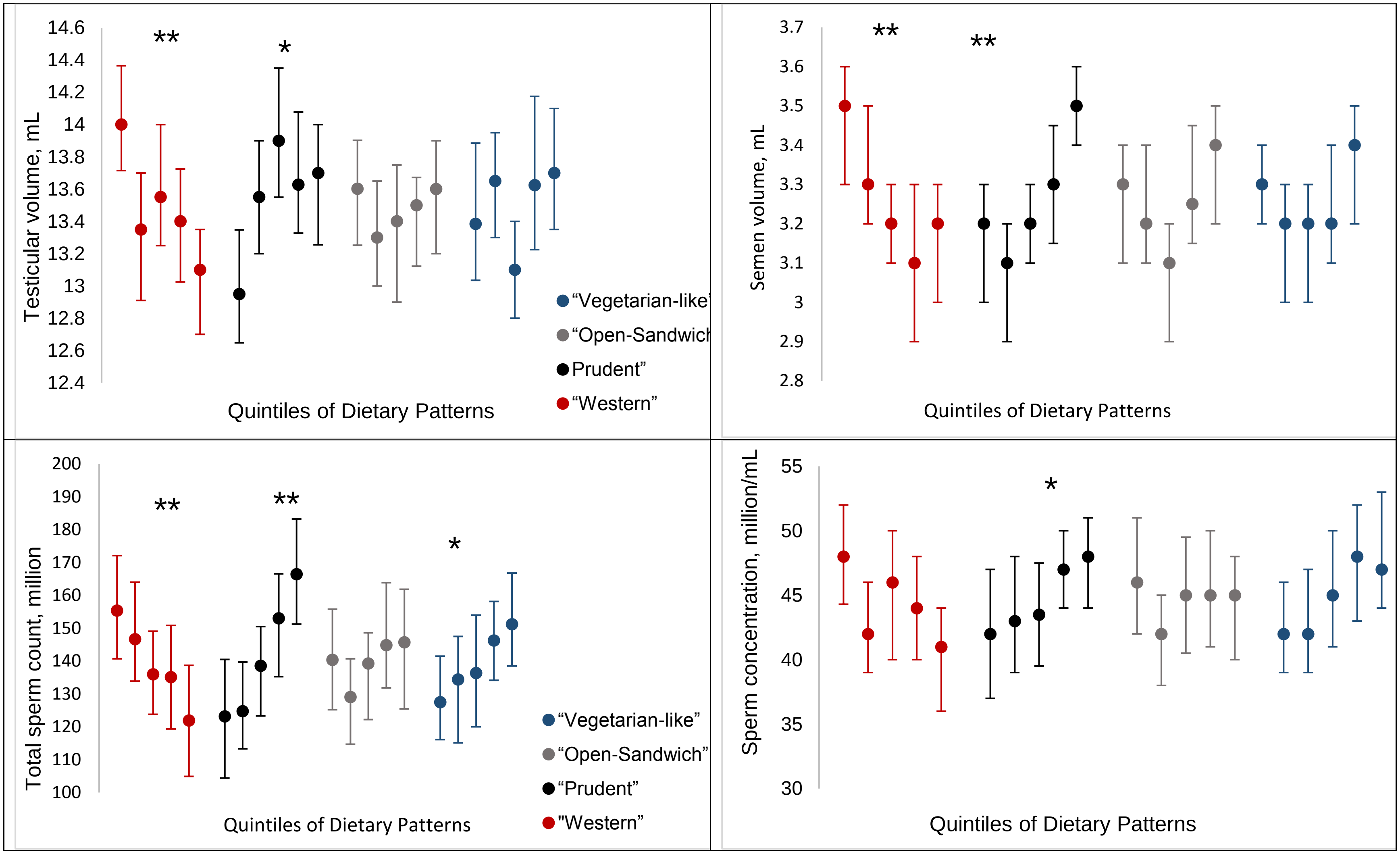

©2020 Nassan FL et al. *JAMA Network Open*.

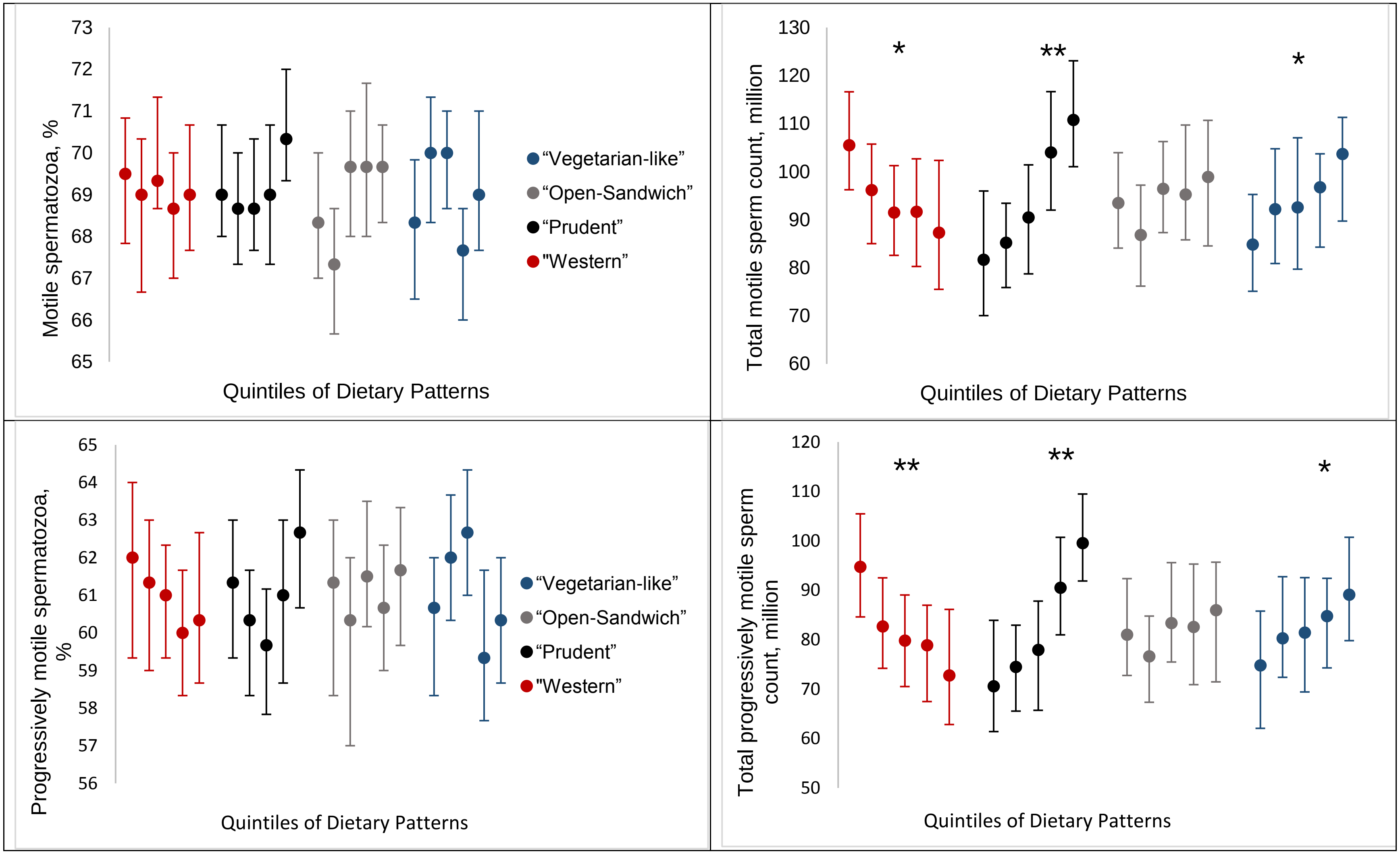

©2020 Nassan FL et al. *JAMA Network Open*.

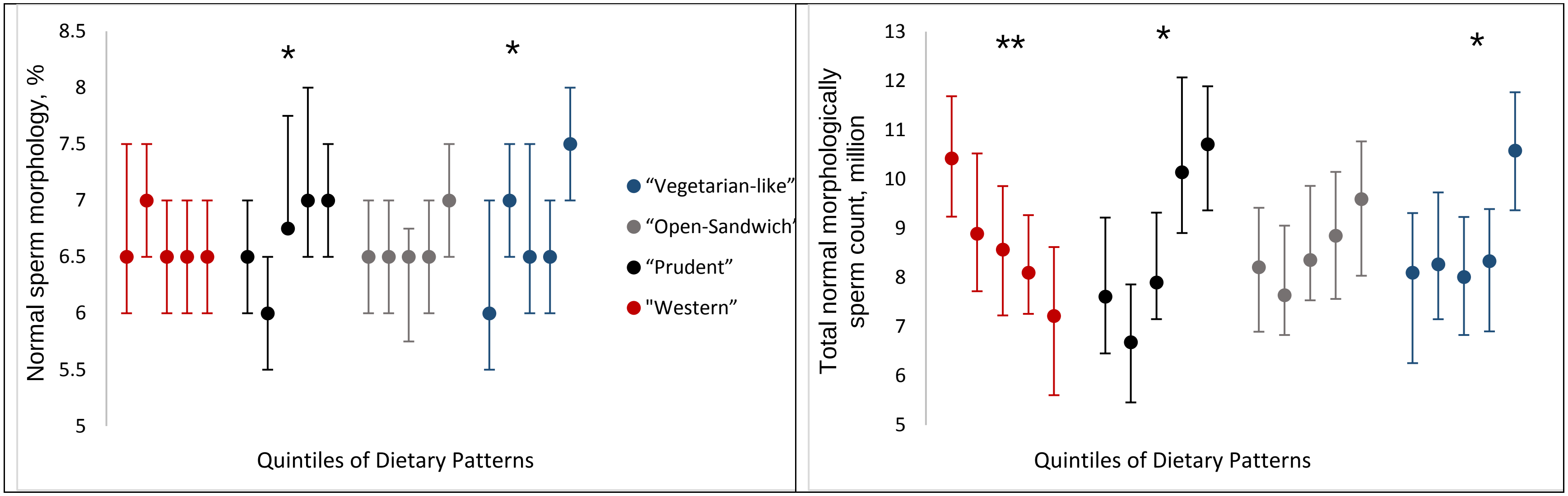

Abbreviations: Q; quintile, 95% CI; 95% confidence intervals.

Distinct colors represent different diet patterns, Red represents the "Western" Pattern, black represents the "Prudent" Pattern, grey represents the "Open-Sandwich" Pattern, and blue represents the "Vegetarian-like" Pattern.

* Tests for trend were conducted across quintiles using the median value in each quintile of the diet patterns as a continuous variable in the regression models and p value was based on Wald test. * indicates <0.05 and ** indicates < 0.01.

©2020 Nassan FL et al. *JAMA Network Open*.

eTable 1. Food Groupings Used in the Dietary Pattern Analyses

Number	**Food groups**	**Food items -Danish**
1	Cold Processed meats	Liver paté or other paté<br>Salami / sausage<br>Saveloy<br>Sausage made of rolled meat<br>Ham or cured saddle of pork<br>Roastbeef<br>Pork filet or saltet pork meat<br>Cold cuts of chicken or turkey<br>Pork roast<br>Fried liver<br>Hamburger steak and meatballs<br>Mayonnaise salat with meat
2	Warm Processed meats	Sausage (e.g. Hotdog/fried sausage)
3	Warm Red meats	Minced meat<br>Beef or veal meat<br>Pork<br>Lamb or motton<br>Hamburger
4	Warm Organ meats	Offal
5	Warm Fish and other seafood	Fatty fish<br>Lean fish<br>Shellfish
6	Cold Fish and other seafood	Smoked herring and mackerel<br>Pickled herring and the like<br>Mackerel in tomato sauce<br>Tuna fish (in water/oil/tomato)<br>Sardines in oil<br>Cod roe<br>Fish cake<br>Fish fillet<br>Shellfish<br>Caviare<br>Smoked salmon and halibut<br>Mayonnaise salat with fish<br>Eel
7	Warm Poultry	Poultry
8	Cold Eggs	Egg (boiled or fried)<br>Bread with egg
9	Warm Eggs	Egg dishes<br>Gratin or pie
10	Low-fat dairy products	Curdled milk products without fruit (fat: 1,5%) or less<br>Curdled milk products with fruit (fat: 1,5%) or less<br>Semi-skimmed milk (fat: 1,5%)<br>Mini-milk (fat: 0,5%)<br>Skimmed milk (fat: 0,1%)<br>Buttermilk
11	High-fat dairy products	Curdled milk products without fruit (fat: 3,5%)<br>Curdled milk products with fruit (fat: 3,5%)<br>Ordinary cheese (slices)<br>Brie, camembert etc.<br>Danish blue cheese, Roquefort etc. (slices)<br>Cottage cheese or smoked fresh cheese (slices)<br>Cheese spread or cream cheese (slices)

©2020 Nassan FL et al. *JAMA Network Open*.

		Icecream<br>Whole milk<br>Chocolate milk
12	Dairy-based sauces	Melted butter or magarine as sauce<br>Bearnaise- or hollandaise-sauce etc.<br>Brown sauce or gravy<br>White sauce (e.g. Béchamel sauce)<br>Cold sauce (e.g. Yoghurt/junket/sour cream)
13	Liquor	Liquor
14	Wine	Red wine<br>White wine
15	Beer	Normal beer<br>Strong beer
16	Tea	Tea<br>Herbal tea
17	Coffee	Coffee
18	Fruit	Fresh fruit<br>Raisins
19	Fruit juices	Fruit juice
20	Tomatoes	Tomato sauce<br>Vegetable juice
21	Vegetable Salads or raw vegetable as a side	Salad and raw vegetables
22	Warm vegeterian main dish	Vegetarian dishes
23	Cooked vegetables except potatos as a side dish	Cooked/prepared vegetables (not potatoes)
24	Soymilk or rice-milk	Soymilk or rice-milk
25	Potatoes	Bread with boiled potatoe<br>Boiled potatoes<br>Baked potatoes<br>Pan fried potatoes<br>Mashed potatoes<br>Cream potatoes or potatoes in white sauce<br>Potato salad
26	French fries	French fries
27	Whole grains	Porridge<br>Rye bread<br>Wholemeal bread<br>Crispbread
28	Cold breakfast cereal	Oatmeal<br>Müsli<br>Cornflakes, Frosties etc.
29	Refined grains	White bread<br>Pasta as garniture<br>Rice, bulgur etc. as garniture
30	Pizza	Pizza
31	Snacks	Chips<br>Pork rinds<br>Popcorn
32	Nuts	Peanuts, pistacies-nutc ect.<br>Other nuts (e.g. Almonds)
33	Suger-sweetened beverages	Coke (caffeinated soft drink with sugar)<br>Other softdrink (with sugar)<br>Fruit syrup with sugar (glass)
34	Low-energy drinks	Diet/light coke

©2020 Nassan FL et al. *JAMA Network Open*.

		Other softdrink (diet/light) than cola<br>Fruit syrup without sugar (diet)
35	Mayonnaise	Bread with mayonnaise and tartare sauce
36	Sweets and desserts	Cake (eg. Pound cake)<br>Danish pastry<br>Cream cakes and layer cakes pastry<br>Cookies, biscuits and crackers<br>Fruit dessert or fruit tart/pie<br>Dark chocolate<br>Milk chocolate<br>A mixed candy<br>Caramels / toffees<br>Drops<br>Liquorice<br>Liquorice allsorts<br>Wine gum / fruit gum<br>Cocoa<br>Pieces of chocolate (50 g)
37	Condiments	Jam or fruit preserves/fruit jelly<br>Honey
38	Water and mineral water	Water<br>Mineral water (e.g., Club soda)
39	Cocktails	Alcopops
40	Energy drink	Energi-drink (eg. Red bull)

©2020 Nassan FL et al. *JAMA Network Open*.

eTable 2. Total Demographic Characteristics Among 2935 Danish Young Men (2008-2017) and According to Adherence to Data-Derived Dietary Patterns

Panel A: Demographic characteristics among 2935 Danish young men (2008-2017).

**Men, N**	**2935**
**Age, years**	19(19,20)
**Body mass index, Kg/m²**	22(21,24)
**Height, m**	1.82(1.77,1.87)
**Cigarettes smoking, N (%)**	
**Daily**	787(26.81)
**Occasionally**	683(23.27)
**Never**	1465(49.91)
**Marijuana smoking, N (%)**	
**Daily**	110(3.75)
**Occasionally**	989(33.7)
**Never**	1836(62.56)
**Other recreational drug use, N (%)**	345(11.75)
**Education of the mother**	
**< 9 years**	92(3.13)
**9-10 years**	725(24.7)
**> 11 years**	1665(56.73)
**Other**	183(6.24)
**Missing/do Not know**	270(9.2)
**Moderate and vigorous physical activities, hr/wk**	8(4,14)
**Fever in last 3 months, N (%)**	239(8.14)
**Self-reported history of reproductive diseases, N (%)**	605(20.61)
**Self-reported history of reproductive surgery, N (%)**	332(11.31)
**Self-reported history of STDs, N (%)**	333(11.35)
**Use of muscle-enhancing products in the last 3 months, N (%)**	752(25.74)
**Abstinence time, hours**	62(57,84)
**Sample collected during warm season, N (%)**	950(32.37)
**Time of day of sample collection, hour**	10(9,11)
**Time to semen analysis, min**	30(20,45)
**Total energy intake, Kcal/day**	1990(1536,2547)
**Dietary protein (% of energy)**	17(16,20)
Total protein, g/day	87(78,98)
**Total dietary fat (% of energy)**	34(30,38)
Total fat, g/day	75(66,83)
**Saturated dietary fat (% of energy)**	13(11,15)
Saturated fatty acids, g/day	29(25,34)
**Monounsaturated dietary fat (% of energy)**	12(10,13)
Mono-unsaturated fatty acids, g/day	26(23,29)
**Polyunsaturated dietary fat (% of energy)**	5(4,6)
Poly-unsaturated fatty acids, g/day	11(10,12)
Eicosapentaenoic acid (EPA) and Docosahexaenoic acid (DHA), mg/day	395(236,655)
N3 Poly unsaturated fatty acids, g/day	2(2,3)
N6 Poly unsaturated fatty acids, g/day	8(7,9)
**Dietary carbohydrate (% of energy)**	48(43,53)
Total carbohydrates, g/day	240(215,264)
Total fiber, g/day	18(14,23)
Total sugar, g/day	81(62,103)
Added sugar, g/day	34(21,53)
Carotenoids, ug /day	2034(1111,4073)

©2020 Nassan FL et al. *JAMA Network Open*.

Retinol, ug/day	722(548,1030)
Vitamin B6, mg /day	1(1,2)
Folic acid, ug /day	247(212,291)
Vitamin B12, ug /day	6(5,8)
Vitamin C, mg /day	64(49,84)
Vitamin D, ug /day	4(3,6)
Vitamin E, α-TE /day	6(5,7)
Vitamin B2, mg /day	2(1,2)
Sodium, mg /day	2934(2582,3257)
Potassium, mg /day	2754(2437,3138)
Iron, mg /day	10(9,11)
Calcium, mg /day	945(737,1227)
Zinc, mg /day	11(10,12)

©2020 Nassan FL et al. *JAMA Network Open*.

eTable 2: Panel B Demographic characteristics according to adherence to data-derived “Western” diet Pattern among 2935 Danish young men (2008-2017).

**Quintiles**	**Q1**	**Q2**	**Q3**	**Q4**	**Q5**	**P-Value**
**Men, N**	587	587	587	587	587	
**Age, years**	19(19,20)	19(19,20)	19(19,20)	19(19,19)	19(19,20)	0.85
**Body mass index, Kg/m²**	22(21,24)	22(21,24)	22(21,24)	22(21,24)	22(20,24)	0.02
**Height, m**	1.82(1.78,1.87)	1.82(1.77,1.87)	1.81(1.77,1.87)	1.82(1.77,1.87)	1.81(1.77,1.86)	0.21
**Cigarettes smoking, N (%)**						<0.0001
**Daily**	83(14.14)	127(21.64)	163(27.77)	181(30.83)	233(39.69)	
**Occasionally**	127(21.64)	148(25.21)	139(23.68)	141(24.02)	128(21.81)	
**Never**	377(64.22)	312(53.15)	285(48.55)	265(45.14)	226(38.5)	
**Marijuana smoking, N (%)**						<0.0001
**Daily**	11(1.87)	10(1.7)	14(2.39)	31(5.28)	44(7.5)	
**Occasionally**	136(23.17)	186(31.69)	217(36.97)	210(35.78)	240(40.89)	
**Never**	440(74.96)	391(66.61)	356(60.65)	346(58.94)	303(51.62)	
**Other recreational drug use, N (%)**	45(7.67)	48(8.18)	61(10.39)	83(14.14)	108(18.4)	<0.0001
**Education of the mother**						0.001
**< 9 years**	14(2.39)	17(2.9)	15(2.56)	18(3.07)	28(4.77)	
**9-10 years**	159(27.09)	147(25.04)	146(24.87)	143(24.36)	130(22.15)	
**> 11 years**	336(57.24)	334(56.9)	337(57.41)	332(56.56)	326(55.54)	
**Other**	44(7.5)	45(7.67)	32(5.45)	38(6.47)	24(4.09)	
**Missing/do Not know**	34(5.79)	44(7.5)	57(9.71)	56(9.54)	79(13.46)	
**Moderate and vigorous physical activities, hr/wk**	9(5,14)	9(5,14)	7(4,13)	7(4,12)	8(4,14)	0.0004
**Fever in last 3 months, N (%)**	43(7.33)	48(8.18)	56(9.54)	48(8.18)	44(7.5)	0.67
**Self-reported history of reproductive diseases, N (%)**	107(18.23)	118(20.1)	111(18.91)	124(21.12)	145(24.7)	0.05
**Self-reported history of reproductive surgery, N (%)**	70(11.93)	68(11.58)	57(9.71)	65(11.07)	72(12.27)	0.68
**Self-reported history of STDs, N (%)**	43(7.33)	59(10.05)	53(9.03)	80(13.63)	98(16.7)	<0.0001
**Use of muscle-enhancing products in the last 3 months, N (%)**	193(33.1)	164(27.99)	148(25.26)	140(24.05)	107(18.32)	<0.0001
**Abstinence time, hours**	62(58,85)	61(57,83)	61(58,83)	61(56,83)	62(56,84)	0.0308
**Sample collected during warm season, N (%)**	204(34.75)	185(31.52)	186(31.69)	181(30.83)	194(33.05)	0.63
**Time of day of sample collection, hour**	10(9,11)	10(9,11)	10(9,11)	10(9,11)	10(10,11)	0.0006
**Time to semen analysis, min**	30(20,45)	30(20,40)	30(20,46)	30(20,45)	30(25,50)	0.0062
**Total energy intake, Kcal/day**	1821(1398,2373)	1800(1380,2357)	1915(1503,2404)	2100(1632,2583)	2303(1847,3015)	<0.0001
**Dietary protein (% of energy)**	18(16,21)	18(16,20)	18(16,20)	17(16,19)	17(15,19)	<0.0001
Total protein, g/day	88(80,101)	89(79,99)	87(78,99)	87(78,96)	85(76,95)	<0.0001
**Total dietary fat (% of energy)**	31(28,35)	34(30,37)	34(30,37)	35(31,39)	34(29,39)	<0.0001
Total fat, g/day	70(63,79)	75(67,83)	75(67,83)	78(69,86)	76(64,86)	<0.0001
**Saturated dietary fat (% of energy)**	12(10,14)	13(12,15)	13(12,15)	14(12,16)	14(11,16)	<0.0001
Saturated fatty acids, g/day	27(23,31)	29(26,33)	30(26,34)	31(27,35)	30(25,34)	<0.0001
**Monounsaturated dietary fat (% of energy)**	11(10,12)	12(10,13)	12(10,13)	12(11,14)	12(10,14)	<0.0001

©2020 Nassan FL et al. *JAMA Network Open*.

Mono-unsaturated fatty acids, g/day	24(22,27)	26(23,29)	26(23,29)	27(24,30)	26(22,30)	<0.0001
**Polyunsaturated dietary fat (% of energy)**	5(4,5)	5(4,6)	5(4,6)	5(5,6)	5(4,6)	0.05
Poly-unsaturated fatty acids, g/day	11(10,12)	11(10,12)	11(10,12)	11(10,13)	11(10,13)	0.02
Eicosapentaenoic acid (EPA) and Docosahexaenoic acid (DHA), mg/day	392(243,683)	415(240,685)	404(243,670)	395(237,620)	365(210,629)	0.17
N3 Poly unsaturated fatty acids, g/day	2(2,2)	2(2,3)	2(2,3)	2(2,3)	2(2,3)	0.22
N6 Poly unsaturated fatty acids, g/day	8(7,9)	8(8,9)	8(7,9)	9(8,10)	8(7,10)	0.006
**Dietary carbohydrate (% of energy)**	50(45,54)	48(43,52)	48(43,52)	46(42,51)	48(42,55)	<0.0001
Total carbohydrates, g/day	253(227,272)	240(219,260)	239(214,260)	233(209,253)	237(209,270)	<0.0001
Total fiber, g/day	24(18,30)	20(15,24)	18(14,22)	17(13,21)	15(12,19)	<0.0001
Total sugar, g/day	75(58,92)	78(62,96)	83(64,104)	81(64,104)	90(67,123)	<0.0001
Added sugar, g/day	23(13,38)	30(19,44)	36(22,54)	39(23,54)	48(30,72)	<0.0001
Carotenoids, ug /day	2568(1318,5248)	2075(1164,3970)	2167(1161,4283)	1836(964,3388)	1788(960,3776)	<0.0001
Retinol, ug/day	715(534,1048)	740(568,1074)	716(542,985)	750(561,1050)	700(530,1011)	0.28
Vitamin B6, mg /day	1(1,2)	1(1,2)	1(1,2)	1(1,1)	1(1,1)	<0.0001
Folic acid, ug /day	271(230,321)	247(217,297)	246(208,283)	236(207,275)	234(199,280)	<0.0001
Vitamin B12, ug /day	7(5,9)	7(5,8)	6(5,8)	6(5,8)	6(5,8)	<0.0001
Vitamin C, mg /day	65(47,87)	63(49,84)	66(52,85)	65(48,83)	63(49,80)	0.25
Vitamin D, ug /day	4(3,6)	5(3,7)	5(3,7)	4(3,6)	4(3,6)	0.03
Vitamin E, α-TE /day	6(5,7)	6(5,7)	6(5,7)	6(5,7)	6(5,7)	0.07
Vitamin B2, mg /day	2(1,2)	2(1,2)	2(1,2)	2(1,2)	2(1,2)	<0.0001
Sodium, mg /day	3141(2761,3465)	2963(2673,3284)	2939(2576,3242)	2872(2565,3184)	2735(2446,3110)	<0.0001
Potassium, mg /day	2880(2551,3246)	2796(2475,3136)	2733(2424,3149)	2700(2406,3058)	2673(2349,3054)	<0.0001
Iron, mg /day	11(10,12)	10(9,11)	10(9,11)	10(9,11)	10(9,11)	<0.0001
Calcium, mg /day	1009(782,1270)	977(757,1249)	925(722,1217)	909(737,1168)	921(700,1212)	0.0003
Zinc, mg /day	12(11,13)	11(10,13)	11(10,12)	11(10,12)	11(10,12)	<0.0001

©2020 Nassan FL et al. *JAMA Network Open*.

eTable 2: Panel C. Demographic characteristics according to adherence to data-derived “Prudent” diet Pattern among 2935 Danish young men (2008-2017).

Quintiles	Q1	Q2	Q3	Q4	Q5	P-Value
**Men, N**	587	587	587	587	587	
**Age, years**	19(19,20)	19(19,20)	19(19,19)	19(19,20)	19(19,20)	0.11
**Body mass index, Kg/m²**	22(20,24)	22(20,24)	22(21,24)	22(21,24)	22(21,24)	0.001
**Height, m**	1.81(1.76,1.86)	1.82(1.78,1.87)	1.82(1.77,1.87)	1.82(1.78,1.87)	1.82(1.78,1.87)	0.001
**Cigarettes smoking, N (%)**						<0.0001
**Daily**	215(36.63)	173(29.47)	158(26.92)	128(21.81)	113(19.25)	
**Occasionally**	116(19.76)	136(23.17)	130(22.15)	155(26.41)	146(24.87)	
**Never**	256(43.61)	278(47.36)	299(50.94)	304(51.79)	328(55.88)	
**Marijuana smoking, N (%)**						0.0001
**Daily**	40(6.81)	23(3.92)	18(3.07)	16(2.73)	13(2.21)	
**Occasionally**	215(36.63)	196(33.39)	198(33.73)	205(34.92)	175(29.81)	
**Never**	332(56.56)	368(62.69)	371(63.2)	366(62.35)	399(67.97)	
**Other recreational drug use, N (%)**	80(13.63)	70(11.93)	72(12.27)	64(10.9)	59(10.05)	0.38
**Education of the mother**						<0.0001
**< 9 years**	32(5.45)	11(1.87)	17(2.9)	18(3.07)	14(2.39)	
**9-10 years**	159(27.09)	159(27.09)	156(26.58)	130(22.15)	121(20.61)	
**> 11 years**	293(49.91)	318(54.17)	342(58.26)	346(58.94)	366(62.35)	
**Other**	22(3.75)	41(6.98)	34(5.79)	44(7.5)	42(7.16)	
**Missing/do Not know**	81(13.8)	58(9.88)	38(6.47)	49(8.35)	44(7.5)	
**Moderate and vigorous physical activities, hr/wk**	6(3,11)	7(4,12)	8(4,13)	9(5,14)	10(7,16)	<0.0001
**Fever in last 3 months, N (%)**	39(6.64)	51(8.69)	50(8.52)	54(9.2)	45(7.67)	0.53
**Self-reported history of reproductive diseases, N (%)**	113(19.25)	127(21.64)	113(19.25)	130(22.15)	122(20.78)	0.63
**Self-reported history of reproductive surgery, N (%)**	58(9.88)	66(11.24)	68(11.58)	61(10.39)	79(13.46)	0.35
**Self-reported history of STDs, N (%)**	87(14.82)	64(10.9)	57(9.71)	62(10.56)	63(10.73)	0.05
**Use of muscle-enhancing products in the last 3 months, N (%)**	90(15.44)	117(20)	122(21)	187(31.91)	236(40.27)	<0.0001
**Abstinence time, hours**	61(56,84)	61(57,83)	61(57,83)	62(58,84)	63(58,85)	0.03
**Sample collected during warm season, N (%)**	179(30.49)	184(31.35)	188(32.03)	195(33.22)	204(34.75)	0.56
**Time of day of sample collection, hour**	10(10,11)	10(9,11)	10(9,11)	10(9,10)	10(9,11)	0.003
**Time to semen analysis, min**	30(22,50)	30(20,45)	30(20,45)	30(20,45)	30(20,45)	0.09
**Total energy intake, Kcal/day**	1881(1416,2482)	1820(1431,2356)	1968(1517,2519)	2011(1592,2517)	2278(1800,2906)	<0.0001
**Dietary protein (% of energy)**	16(15,18)	17(15,19)	17(16,19)	18(16,20)	19(16,21)	<0.0001

©2020 Nassan FL et al. *JAMA Network Open*.

Total protein, g/day	81(74,90)	86(77,95)	87(79,96)	90(80,100)	94(83,106)	<0.0001
**Total dietary fat (% of energy)**	33(29,38)	34(30,38)	34(30,38)	33(30,37)	33(29,37)	0.44
Total fat, g/day	74(65,84)	75(66,84)	76(67,84)	74(66,82)	74(65,83)	0.36
**Saturated dietary fat (% of energy)**	13(11,15)	13(12,15)	13(12,15)	13(11,15)	13(11,15)	0.02
Saturated fatty acids, g/day	30(25,34)	30(26,34)	30(26,34)	29(25,33)	29(24,33)	0.004
**Monounsaturated dietary fat (% of energy)**	11(10,13)	12(10,13)	12(10,13)	12(10,13)	12(10,13)	0.55
Mono-unsaturated fatty acids, g/day	25(22,30)	26(23,29)	26(23,30)	26(23,29)	26(23,29)	0.57
**Polyunsaturated dietary fat (% of energy)**	5(4,6)	5(4,6)	5(5,6)	5(4,6)	5(5,6)	<0.0001
Poly-unsaturated fatty acids, g/day	11(9,12)	11(10,12)	11(10,13)	11(10,13)	11(10,13)	<0.0001
Eicosapentaenoic acid (EPA) and Docosahexaenoic acid (DHA), mg/day	316(198,562)	355(218,569)	386(233,614)	422(259,680)	506(302,856)	<.0001
N3 Poly unsaturated fatty acids, g/day	2(2,2)	2(2,3)	2(2,3)	2(2,3)	2(2,3)	<0.0001
N6 Poly unsaturated fatty acids, g/day	8(7,9)	8(7,9)	9(8,10)	8(8,9)	9(8,10)	0.0005
**Dietary carbohydrate (% of energy)**	49(45,54)	48(44,53)	48(43,53)	48(43,52)	47(42,52)	<0.0001
Total carbohydrates, g/day	247(224,271)	241(219,265)	239(213,264)	239(215,259)	233(206,259)	<0.0001
Total fiber, g/day	18(13,22)	18(13,23)	19(14,23)	19(15,24)	19(15,24)	<0.0001
Total sugar, g/day	92(70,119)	82(63,104)	78(62,99)	79(61,97)	76(58,96)	<0.0001
Added sugar, g/day	47(30,68)	37(23,55)	32(22,49)	31(17,47)	27(16,43)	<0.0001
Carotenoids, ug /day	1353(786,2856)	1610(992,2912)	2062(1158,3752)	2523(1468,4608)	3348(1746,6093)	<0.0001
Retinol, ug/day	738(558,1025)	757(552,1093)	707(533,984)	711(556,992)	709(533,1034)	0.23
Vitamin B6, mg /day	1(1,1)	1(1,1)	1(1,1)	1(1,2)	1(1,2)	<0.0001
Folic acid, ug /day	228(196,269)	235(202,271)	244(212,287)	260(221,304)	271(229,324)	<0.0001
Vitamin B12, ug /day	6(5,8)	6(5,8)	6(5,8)	6(5,8)	7(5,9)	<0.0001
Vitamin C, mg /day	58(45,74)	60(46,79)	66(51,84)	70(55,87)	71(54,93)	<0.0001
Vitamin D, ug /day	4(3,5)	4(3,6)	4(3,6)	5(3,7)	5(4,8)	<0.0001

©2020 Nassan FL et al. *JAMA Network Open*.

Vitamin E, α-TE /day	5(5,6)	6(5,6)	6(5,7)	6(5,7)	6(5,7)	<0.0001
Vitamin B2, mg /day	2(1,2)	2(1,2)	2(1,2)	2(1,2)	2(1,2)	0.0002
Sodium, mg /day	2857(2488,3203)	2947(2573,3255)	2970(2608,3280)	2941(2645,3253)	2928(2586,3269)	0.03
Potassium, mg /day	2632(2315,3036)	2672(2374,3027)	2729(2413,3046)	2812(2526,3197)	2922(2592,3287)	<0.0001
Iron, mg /day	10(9,11)	10(9,11)	10(9,11)	10(10,11)	11(10,12)	<0.0001
Calcium, mg /day	945(743,1229)	938(736,1221)	939(736,1231)	963(732,1220)	952(742,1230)	0.99
Zinc, mg /day	11(10,12)	11(10,12)	11(10,12)	12(10,13)	12(11,13)	<0.0001

©2020 Nassan FL et al. *JAMA Network Open*.

eTable 2: Panel D. Demographic characteristics according to adherence to data-derived "Open-Sandwich" diet Pattern among 2935 Danish young men (2008-2017).

**Quintiles**	**Q1**	**Q2**	**Q3**	**Q4**	**Q5**	**P-Value**
**Men, N**	587	587	587	587	587	
**Age, years**	19(19,20)	19(19,20)	19(19,20)	19(19,20)	19(19,20)	0.15
**Body mass index, Kg/m^2**	22(20,24)	22(21,24)	22(20,24)	22(21,24)	22(21,24)	0.59
**Height, m**	1.81(1.76,1.86)	1.82(1.77,1.87)	1.82(1.78,1.86)	1.83(1.78,1.87)	1.83(1.78,1.88)	<.0001
**Cigarettes smoking, N (%)**						0.02
**Daily**	180(30.66)	164(27.94)	164(27.94)	144(24.53)	135(23)	
**Occasionally**	138(23.51)	150(25.55)	135(23)	126(21.47)	134(22.83)	
**Never**	269(45.83)	273(46.51)	288(49.06)	317(54)	318(54.17)	
**Marijuana smoking, N (%)**						0.11
**Daily**	25(4.26)	25(4.26)	15(2.56)	27(4.6)	18(3.07)	
**Occasionally**	213(36.29)	208(35.43)	207(35.26)	179(30.49)	182(31.01)	
**Never**	349(59.45)	354(60.31)	365(62.18)	381(64.91)	387(65.93)	
**Other recreational drug use, N (%)**	77(13.12)	66(11.24)	70(11.93)	70(11.93)	62(10.56)	0.73
**Education of the mother**						0.004
**< 9 years**	34(5.79)	12(2.04)	18(3.07)	12(2.04)	16(2.73)	
**9-10 years**	149(25.38)	145(24.7)	142(24.19)	145(24.7)	144(24.53)	
**> 11 years**	301(51.28)	336(57.24)	334(56.9)	360(61.33)	334(56.9)	
**Other**	38(6.47)	31(5.28)	40(6.81)	34(5.79)	40(6.81)	
**Missing/do Not know**	65(11.07)	63(10.73)	53(9.03)	36(6.13)	53(9.03)	
**Moderate and vigorous physical activities, hr/wk**	6(3,12)	7(4,13)	7(4,12)	9(5,14)	10(6,15)	<.0001
**Fever in last 3 months, N (%)**	61(10.39)	50(8.52)	40(6.81)	41(6.98)	47(8.01)	0.16
**Self-reported history of reproductive diseases, N (%)**	104(17.72)	116(19.76)	122(20.78)	129(21.98)	134(22.83)	0.22
**Self-reported history of reproductive surgery, N (%)**	62(10.56)	57(9.71)	75(12.78)	72(12.27)	66(11.24)	0.46
**Self-reported history of STDs, N (%)**	84(14.31)	68(11.58)	60(10.22)	60(10.22)	61(10.39)	0.13
**Use of muscle-enhancing products in the last 3 months, N (%)**	138(23.55)	136(23.41)	119(20.45)	168(28.72)	191(32.54)	<.0001
**Abstinence time, hours**	62(58,84)	61(55,82)	62(58,84)	62(58,85)	61(57,84)	0.009
**Sample collected during warm season, N (%)**	201(34.24)	184(31.35)	198(33.73)	196(33.39)	171(29.13)	0.31
**Time of day of sample collection, hour**	10(9,11)	10(10,11)	10(9,11)	10(9,10)	10(9,11)	0.33
**Time to semen analysis, min**	30(23,40)	30(20,45)	30(22,50)	30(20,45)	30(20,50)	0.11
**Total energy intake, Kcal/day**	1637(1260,2201)	1749(1402,2176)	1921(1543,2357)	2116(1751,2553)	2596(2021,3257)	<.0001
**Dietary protein (% of energy)**	18(16,21)	17(16,20)	17(15,20)	17(15,19)	17(15,19)	<.0001
Total protein, g/day	89(79,100)	86(78,97)	86(77,98)	87(78,98)	86(78,98)	0.07
**Total dietary fat (% of energy)**	34(31,38)	34(30,38)	33(29,37)	33(29,37)	34(29,38)	<.0001
Total fat, g/day	76(68,85)	75(67,85)	74(64,82)	73(64,81)	75(65,84)	<.0001
**Saturated dietary fat (% of energy)**	13(12,15)	13(12,15)	13(11,15)	13(11,15)	13(11,15)	0.010
Saturated fatty acids, g/day	30(26,34)	30(26,34)	29(25,33)	29(25,33)	29(25,33)	0.001
**Monounsaturated dietary fat (% of energy)**	12(11,13)	12(10,13)	12(10,13)	11(10,13)	12(10,13)	0.0001

©2020 Nassan FL et al. *JAMA Network Open*.

Mono-unsaturated fatty acids, g/day	27(23,30)	26(23,30)	26(22,29)	25(22,28)	26(23,30)	<.0001
**Polyunsaturated dietary fat (% of energy)**	5(5,6)	5(4,6)	5(4,5)	5(4,6)	5(4,6)	<.0001
Poly-unsaturated fatty acids, g/day	11(10,13)	11(10,13)	11(10,12)	11(10,12)	11(10,13)	0.0002
Eicosapentaenoic acid (EPA) and Docosahexaenoic acid (DHA), mg/day	400(241,678)	415(248,642)	400(243,626)	390(229,658)	376(218,720)	0.63
N3 Poly unsaturated fatty acids, g/day	2(2,2)	2(2,3)	2(2,2)	2(2,3)	2(2,3)	0.27
N6 Poly unsaturated fatty acids, g/day	9(8,10)	8(8,9)	8(7,9)	8(7,9)	9(7,10)	0.002
**Dietary carbohydrate (% of energy)**	46(41,51)	48(43,52)	49(44,54)	49(44,54)	49(44,53)	<.0001
Total carbohydrates, g/day	231(206,255)	239(215,261)	243(219,269)	245(222,268)	241(218,265)	<.0001
Total fiber, g/day	16(12,20)	18(13,22)	19(14,24)	20(16,25)	20(15,25)	<.0001
Total sugar, g/day	82(61,102)	81(64,103)	83(64,108)	81(63,102)	78(60,99)	0.02
Added sugar, g/day	38(22,55)	35(23,54)	34(21,54)	33(20,51)	30(18,50)	0.0002
Carotenoids, ug /day	2352(1215,4603)	2005(1161,4091)	2080(1114,4107)	1937(1084,3667)	1985(1013,3860)	0.004
Retinol, ug/day	696(556,960)	711(547,1001)	709(543,957)	745(558,1096)	777(527,1201)	0.02
Vitamin B6, mg /day	1(1,2)	1(1,2)	1(1,2)	1(1,2)	1(1,2)	0.02
Folic acid, ug /day	240(199,292)	247(212,291)	244(213,287)	250(215,297)	250(216,293)	0.05
Vitamin B12, ug /day	6(5,8)	6(5,8)	6(5,8)	6(5,8)	7(5,9)	0.05
Vitamin C, mg /day	67(51,91)	65(50,84)	63(46,83)	63(48,83)	64(49,80)	0.02
Vitamin D, ug /day	5(3,7)	4(3,6)	4(3,6)	4(3,6)	4(3,6)	0.01
Vitamin E, α-TE /day	6(5,7)	6(5,7)	6(5,7)	6(5,6)	6(5,7)	<.0001
Vitamin B2, mg /day	2(1,2)	2(1,2)	2(1,2)	2(1,2)	2(1,2)	<.0001
Sodium, mg /day	2783(2474,3096)	2895(2562,3217)	2925(2570,3229)	2998(2645,3280)	3098(2701,3501)	<.0001
Potassium, mg /day	2711(2376,3090)	2737(2412,3103)	2767(2438,3172)	2805(2499,3152)	2770(2453,3142)	0.03
Iron, mg /day	10(9,11)	10(9,11)	10(9,11)	10(9,11)	11(9,11)	<.0001
Calcium, mg /day	881(688,1146)	944(740,1231)	1021(763,1276)	971(757,1230)	955(743,1231)	<.0001
Zinc, mg /day	11(10,12)	11(10,12)	11(10,12)	11(10,13)	12(10,13)	<.0001

eTable 2: Panel E. Demographic characteristics according to adherence to data-derived “Vegetarian-like” diet Pattern among 2935 Danish young men (2008-2017).

**Quintiles**	**Q1**	**Q2**	**Q3**	**Q4**	**Q5**	**P-Value**
**Men, N**	587	587	587	587	587	
**Age, years**	19(19,19)	19(19,20)	19(19,20)	19(19,19)	19(19,20)	<.0001
**Body mass index, Kg/m²**	23(21,25)	22(20,24)	22(21,24)	22(20,24)	22(20,24)	<.0001
**Height, m**	1.82(1.77,1.86)	1.81(1.77,1.87)	1.82(1.77,1.87)	1.82(1.78,1.87)	1.82(1.77,1.87)	0.81
**Cigarettes smoking, N (%)**						0.15
**Daily**	159(27.09)	175(29.81)	137(23.34)	163(27.77)	153(26.06)	
**Occasionally**	137(23.34)	140(23.85)	128(21.81)	128(21.81)	150(25.55)	
**Never**	291(49.57)	272(46.34)	322(54.86)	296(50.43)	284(48.38)	

©2020 Nassan FL et al. *JAMA Network Open*.

**Marijuana smoking, N (%)**						0.13
**Daily**	20(3.41)	19(3.24)	24(4.09)	18(3.07)	29(4.94)	
**Occasionally**	190(32.37)	194(33.05)	183(31.18)	198(33.73)	224(38.16)	
**Never**	377(64.22)	374(63.71)	380(64.74)	371(63.2)	334(56.9)	
**Other recreational drug use, N (%)**	72(12.27)	77(13.12)	52(8.86)	70(11.93)	74(12.61)	0.17
**Education of the mother**						0.001
**< 9 years**	14(2.39)	18(3.07)	23(3.92)	17(2.9)	20(3.41)	
**9-10 years**	160(27.26)	181(30.83)	137(23.34)	146(24.87)	101(17.21)	
**> 11 years**	325(55.37)	304(51.79)	328(55.88)	335(57.07)	373(63.54)	
**Other**	34(5.79)	37(6.3)	43(7.33)	38(6.47)	31(5.28)	
**Missing/do Not know**	54(9.2)	47(8.01)	56(9.54)	51(8.69)	62(10.56)	
**Moderate and vigorous physical activities, hr/wk**	9(4,15)	8(4,14)	8(4,13)	8(4,13)	8(4,14)	0.23
**Fever in last 3 months, N (%)**	46(7.84)	47(8.01)	47(8.01)	59(10.05)	40(6.81)	0.36
**Self-reported history of reproductive diseases, N (%)**	127(21.64)	102(17.38)	128(21.81)	130(22.15)	118(20.1)	0.23
**Self-reported history of reproductive surgery, N (%)**	64(10.9)	67(11.41)	78(13.29)	70(11.93)	53(9.03)	0.23
**Self-reported history of STDs, N (%)**	72(12.27)	55(9.37)	61(10.39)	75(12.78)	70(11.93)	0.32
**Use of muscle-enhancing products in the last 3 months, N (%)**	211(35.95)	161(27.62)	141(24.06)	130(22.22)	109(18.79)	<.0001
**Abstinence time, hours**	62(58,84)	61(56,83)	62(58,83)	61(57,83)	62(56,85)	0.12
**Sample collected during warm season, N (%)**	187(31.86)	191(32.54)	185(31.52)	196(33.39)	191(32.54)	0.97
**Time of day of sample collection, hour**	10(9,11)	10(9,11)	10(9,11)	10(9,11)	10(10,11)	0.21
**Time to semen analysis, min**	30(25,45)	30(20,45)	30(20,45)	30(20,45)	30(25,45)	0.31
**Total energy intake, Kcal/day**	2050(1610,262 1)	1881(1450,233 4)	1949(1483,252 1)	1979(1535,252 2)	2118(1629,265 5)	<.0001
**Dietary protein (% of energy)**	18(16,21)	18(16,20)	17(15,19)	17(15,19)	17(15,19)	<.0001
Total protein, g/day	92(83,105)	89(79,99)	86(77,95)	85(76,96)	85(76,94)	<.0001
**Total dietary fat (% of energy)**	35(30,38)	33(29,38)	33(30,37)	33(30,37)	33(29,37)	0.002
Total fat, g/day	77(67,85)	74(65,84)	74(66,83)	74(66,82)	73(64,82)	0.002
**Saturated dietary fat (% of energy)**	13(12,15)	13(11,15)	13(11,15)	13(12,15)	13(11,15)	0.31
Saturated fatty acids, g/day	30(26,34)	29(25,34)	29(25,34)	29(26,34)	29(25,33)	0.32
**Monounsaturated dietary fat (% of energy)**	12(11,14)	12(10,13)	12(10,13)	12(10,13)	11(10,13)	<.0001
Mono-unsaturated fatty acids, g/day	27(24,31)	26(22,30)	26(23,29)	26(22,29)	25(22,29)	<.0001
**Polyunsaturated dietary fat (% of energy)**	5(5,6)	5(4,6)	5(4,6)	5(4,6)	5(4,6)	0.007
Poly-unsaturated fatty acids, g/day	11(10,13)	11(10,13)	11(10,12)	11(10,12)	11(10,12)	0.005
Eicosapentaenoic acid (EPA) and Docosahexaenoic acid (DHA), mg/day	357(211,613)	394(238,647)	406(239,685)	405(250,650)	405(243,722)	0.03
N3 Poly unsaturated fatty acids, g/day	2(2,3)	2(2,3)	2(2,3)	2(2,3)	2(2,3)	0.98
N6 Poly unsaturated fatty acids, g/day	9(8,10)	8(7,9)	8(8,9)	8(7,9)	8(7,9)	<.0001
**Dietary carbohydrate (% of energy)**	45(41,51)	48(42,53)	48(44,53)	49(44,53)	49(44,54)	<.0001
Total carbohydrates, g/day	227(203,252)	239(214,264)	243(221,264)	243(222,267)	246(220,271)	<.0001
Total fiber, g/day	18(13,22)	19(14,24)	18(14,24)	19(15,24)	19(14,24)	0.001

©2020 Nassan FL et al. *JAMA Network Open*.

Total sugar, g/day	74(56,96)	79(60,103)	82(63,103)	84(66,104)	87(67,107)	<.0001
Added sugar, g/day	28(16,46)	32(19,51)	35(22,52)	36(23,55)	40(25,59)	<.0001
Carotenoids, ug /day	1661(890,3028)	1862(1033,3668)	1982(1087,3774)	2189(1226,4430)	2879(1387,5671)	<.0001
Retinol, ug/day	709(535,972)	745(548,1053)	730(565,1030)	746(555,1072)	686(538,1004)	0.14
Vitamin B6, mg /day	1(1,2)	1(1,2)	1(1,2)	1(1,1)	1(1,1)	<.0001
Folic acid, ug /day	232(200,274)	242(206,284)	244(210,291)	251(220,297)	264(225,314)	<.0001
Vitamin B12, ug /day	7(5,8)	6(5,8)	6(5,8)	6(5,8)	6(5,8)	0.001
Vitamin C, mg /day	62(48,81)	64(48,82)	63(49,85)	65(50,83)	68(50,88)	0.02
Vitamin D, ug /day	4(3,6)	5(3,6)	5(3,7)	4(3,6)	4(3,6)	0.95
Vitamin E, α-TE /day	6(5,7)	6(5,7)	6(5,7)	6(5,7)	6(5,7)	0.0005
Vitamin B2, mg /day	2(1,2)	2(1,2)	2(1,2)	2(1,2)	2(1,2)	0.14
Sodium, mg /day	2976(2625,3306)	2951(2514,3341)	2920(2554,3235)	2941(2641,3215)	2882(2549,3234)	0.05
Potassium, mg /day	2775(2438,3119)	2743(2414,3118)	2688(2401,3112)	2738(2423,3167)	2833(2501,3163)	0.003
Iron, mg /day	10(9,11)	10(9,11)	10(9,11)	10(9,11)	10(9,12)	0.48
Calcium, mg /day	954(703,1257)	932(741,1222)	955(735,1181)	949(741,1260)	944(764,1193)	0.57
Zinc, mg /day	12(11,13)	11(10,13)	11(10,12)	11(10,12)	11(10,12)	<.0001

Abbreviations: N; number of men, m; meters, hr; hours, wk; week, STDs; sexually transmitted Diseases, α-TE ; alpha-tocopherol equivalents.

Numbers shown are median and interquartile range unless noted as N(%).

Warm season: April through September.

Reproductive diseases include self-reported history of varicocele, cryptorchidism, testicular mumps, inguinal hernia, testicular injury (hit, kicked or otherwise injured so it caused swelling of the scrotum), hydrocele, testicular torsion, hypospadias, epididymo-orchites, cystitis or prostatitis.

Reproductive surgeries include self-reported history of surgery for inguinal hernia, varicocele, hydrocele, testicular torsion, hypospadias, testicular cancer, phimosis, testicular biopsy, vasectomy, re-fertilization, and other reproductive surgeries.

STDs included self-reported history of gonorrhea, chlamydia, and other venereal diseases.

All nutrients' intakes were adjusted for energy intake using the residual method making nutrient intake independent from energy intake.

P-values were estimated based on Chi-square test for categorical variables and Kruskal-Wallis test for continuous variables to test for differences in demographic and nutritional characteristics across quintiles of adherence within each pattern.

©2020 Nassan FL et al. *JAMA Network Open*.

eTable 3. Food Group Loadings for 2 Dietary Patterns Identified From Food-Frequency Questionnaire

Rotated Factor Pattern	"Western" Pattern		"Prudent" Pattern		"Open-Sandwich" Pattern		"Vegetarian-like" Pattern	
Pizza	64	*	-5		-8		-8	
French Fries	61	*	-3		3		-6	
Warm Processed Meat	58	*	3		3		-8	
Warm Red Meat	55	*	31		12		-32	
Snacks	52	*	2		2		10	
Dairy Based Sauces	49	*	8		20		-2	
Potatoes	48	*	16		41	*	5	
High Energy Drinks	47	*	-27		10		8	
Sweets Desserts	37		-7		5		16	
Refined Grains	31		-1		24		8	
Warm Organ Meat	30		19		3		17	
Energy Drink	29		-18		2		11	
Fruit Juices	18		7		16		16	
Liquor	14		1		-3		-23	
Beer	13		-4		0		-4	
Cocktails	10		-5		3		-15	
Vegetable Salad	-11		65	*	1		7	
Cooked Vegetable Side	-11		65	*	1		14	
Warm Fish	17		53	*	9		14	
Warm Poultry	9		51	*	-6		-28	
Fruit	-22		52	*	21		17	
Nuts	9		49	*	2		17	
Water	-16		41	*	16		-1	
Cold Eggs	10		35		29		7	
Cold Cereal	-14		30		30		9	
Wine	2		11		-9		10	
Cold Processed Meat	10		5		76	*	-12	
Whole Grains	-14		11		68	*	7	
Mayonnaise	7		-8		54	*	-6	
Cold Fish	18		16		53	*	6	
Condiments	8		0		48	*	24	
High Fat Dairy	24		1		40		32	
Low Fat Dairy	-4		20		35		-17	
Low Energy Drinks	6		1		16		-7	
Warm Vegetarian Main	-5		13		-13		64	*
Tea	8		-3		3		41	*
Soymilk	-3		4		0		39	
Warm Eggs	29		24		0		37	
Tomatoes	18		17		10		37	
Coffee	4		3		1		23	

Printed values are multiplied by 100 and rounded to the nearest integer.
Values greater than 0.4 are flagged by an '*'.

©2020 Nassan FL et al. *JAMA Network Open*.

eTable 4. **Summary of the Distribution of Reproductive Parameters**

Reproductive Parameter	N	Median (95% CI)	Mean (SD)	% < WHO lower limits	% Intermediate semen quality	%≥ High semen quality (Damsgaard)
**Semen volume, mL**	2798	3.2 (3.2,3.3)	3.4 (1.5)	6	-	-
**Sperm concentration, million/mL**	2798	44 (42,46)	57(50)	17	28	55
**Total sperm count, million**	2798	140 (133,146)	183(165)	15	-	-
**Motile spermatozoa, %**	2798	69 (68,70)	66 (16)	8	6	87
**Total motile sperm count, million**	2798	14 (13, 14)	127 (119)	-	-	-
**Progressively motile spermatozoa, %**	2798	61 (60,62)	58 (17)	8	-	-
**Total progressively motile sperm count, million**	2798	82 (77, 86)	113(109)	-		
**Normal sperm morphology, %**	2798	6.5 (6.5,7.0)	7.2 (4.6)	25	41	34
**Total normal morphologically sperm count, million**	2798	9 (8,9)	16(20)	-	-	-
**Testicular volume by ultrasound, mean (mL)**	2798	13.5 (13.3,13.7)	13.8 (3.8)	-	-	-
**Total testosterone, nmol/L**	2734	18 (18,18)	19 (6)	-	-	-
**Free testosterone, pmol/L**	2734	445 (441,453)	468(154)	-	-	-
**Estradiol, pmol/l**	2734	82 (81,83)	85(30)	-	-	-
**LH, IU/L**	2734	3.3 (3.3,3.4)	3.6 (1.6)	-	-	-
**FSH, IU/L**	2734	2.6 (2.5,2.7)	3.0(1.9)	-	-	-
**SHBG, Nmol/L**	2734	28 (28,29)	30 (11)	-	-	-
**Inhibin-B, pg/mL**	2734	168 (166,170)	174 (61)	-	-	-
**Inhibin-B/ FSH**	2734	65(63,69)	99 (507)	-	-	-
**Total Testosterone/ LH**	2734	6(5,6)	6(10)	-	-	-
**Free Testosterone/ LH**	2734	135(133,137)	159 (463)	-	-	-
**Estradiol/ Total Testosterone**	2734	4(4,4)	5 (2)	-	-	-
**(Estradiol/ Free Testosterone) *100**	2734	18(18,18)	19(7)	-	-	-

Abbreviations: N; number of men, 95% CI; 95% confidence intervals, SD; standard deviation, WHO; world health organization.
WHO lower reference limits are < 1.5 mL semen volume, <15 million/mL sperm concentration, < 39 million total sperm count, <40 % motile spermatozoa, <32 % progressively motile spermatozoa, and < 4% normal sperm morphology.
Damsgaard reference limits: ≥40 mill/ml sperm concentration, ≥50%motile spermatozoa, and ≥9% normal sperm morphology.
Intermediate semen quality: between the WHO lower reference limits and Damsgaard reference limits.

©2020 Nassan FL et al. *JAMA Network Open*.

eTable 5. Unadjusted Medians in Serum Concentrations of Reproductive Hormones on the Scale for the Individual Hormones as Stated in the Table According to Adherence to Data-Derived Dietary Patterns

	Reproductive Parameters	Total Testosterone, nmol/L	Free Testosterone, pmol/L	Estradiol, pg/mL	LH, IU/L	Inhibin-B, pg/mL	FSH, IU/L	SHBG, nmol/L	Inhibin-B/ FSH	Total Testosterone/ LH	Free Testosterone/ LH	Estradiol/ Total Testosterone	(Estradiol/ Free Testosterone)*100
"Western"	Q1	17 (17,18)	415 (405,426)	80 (78,83)	3.3 (3.2,3.4)	171 (166,175)	2.5 (2.4,2.7)	28 (27,29)	71 (61,77)	6 (5,6)	127 (122,133)	5 (4,5)	19 (18,19)
	Q2	18 (17,18)	437 (426,446)	80 (78,84)	3.4 (3.2,3.6)	165 (161,171)	2.6 (2.5,2.8)	28 (27,29)	65 (59,70)	5 (5,6)	133 (126,137)	4 (4,5)	19 (18,19)
	Q3	18 (18,19)	451 (438,463)	82 (80,86)	3.3 (3.1,3.5)	177 (170,181)	2.6 (2.4,2.7)	27 (27,29)	73 (65,80)	6 (5,6)	135 (130,142)	4 (4,5)	18 (17,19)
	Q4	19 (18,19)	458 (442,470)	82 (80,84)	3.4 (3.3,3.5)	169 (163,174)	2.7 (2.5,2.8)	29 (28,30)	62 (57,68)	6 (5,6)	135 (130,142)	4 (4,4)	18 (17,18)
	Q5	19 (19,20)	473 (462,489)	83 (81,87)	3.4 (3.3,3.5)	160 (154,166)	2.6 (2.4,2.8)	28 (27,29)	62 (57,67)	6 (6,6)	143 (136,148)	4 (4,4)	17 (17,18)
	P trend	<0.0001	<0.0001	0.11	0.25	0.004	0.35	0.92	0.008	0.05	0.003	0.002	<0.0001
"Prudent"	Q1	19 (18,19)	459 (445,473)	85 (83,88)	3.4 (3.3,3.5)	166 (160,171)	2.5 (2.4,2.7)	28 (27,29)	66 (63,72)	6 (5,6)	133 (126,140)	4 (4,4)	18 (17,18)
	Q2	18 (18,19)	451 (438,464)	82 (80,85)	3.4 (3.3,3.6)	167 (163,172)	2.7 (2.5,2.9)	28 (27,29)	63 (57,70)	5 (5,6)	134 (129,139)	4 (4,5)	18 (17,19)
	Q3	18 (18,19)	439 (427,454)	81 (79,83)	3.2 (3.1,3.4)	175 (170,183)	2.4 (2.3,2.6)	28 (27,29)	75 (69,80)	6 (6,6)	140 (134,146)	4 (4,5)	18 (17,19)
	Q4	18 (18,19)	445 (437,457)	80 (78,83)	3.4 (3.2,3.5)	167 (162,173)	2.7 (2.5,2.9)	28 (27,29)	61 (56,69)	6 (5,6)	133 (127,137)	4 (4,5)	18 (18,19)
	Q5	18 (17,18)	435 (420,451)	80 (78,84)	3.3 (3.2,3.5)	166 (161,171)	2.6 (2.6,2.9)	30 (28,31)	60 (56,69)	6 (5,6)	135 (129,141)	4 (4,5)	18 (18,19)
	P trend	0.03	0.03	0.01	0.67	0.90	0.11	0.02	0.05	0.87	0.69	0.37	0.73
"Open-Sandwich"	Q1	18 (18,19)	456 (440,465)	83 (80,85)	3.3 (3.2,3.4)	170 (165,177)	2.6 (2.4,2.7)	27 (26,28)	66 (61,74)	6 (5,6)	141 (137,148)	4 (4,5)	18 (17,19)
	Q2	18 (18,19)	455 (441,471)	82 (80,86)	3.3 (3.2,3.5)	166 (161,172)	2.6 (2.5,2.8)	28 (27,29)	67 (61,71)	6 (5,6)	140 (135,146)	4 (4,5)	18 (17,19)
	Q3	18 (18,19)	445 (435,456)	81 (79,84)	3.3 (3.2,3.5)	173 (167,180)	2.5 (2.4,2.7)	28 (27,29)	68 (61,76)	6 (5,6)	133 (129,137)	4 (4,5)	18 (17,19)
	Q4	18 (18,19)	435 (425,452)	82 (79,84)	3.4 (3.3,3.6)	169 (165,175)	2.6 (2.4,2.7)	29 (28,30)	65 (60,72)	6 (5,6)	132 (125,135)	4 (4,5)	18 (18,19)
	Q5	18 (18,19)	441 (428,453)	82 (79,84)	3.4 (3.3,3.6)	163 (156,168)	2.7 (2.5,2.9)	29 (28,31)	62 (57,66)	5 (5,6)	129 (124,136)	4 (4,4)	18 (17,19)
	P trend	0.10	0.31	0.85	0.05	0.02	0.26	0.01	0.21	0.38	0.0002	0.004	0.74

©2020 Nassan FL et al. *JAMA Network Open*.

"Vegetarian-like"	Q1	18(17,18)	444 (433,455)	82 (79,86)	3.4 (3.3,3.5)	168 (161,174)	2.6 (2.4,2.7)	27 (26,28)	68 (64,73)	5 (5,6)	134 (128,139)	5 (4,5)	19 (18,19)
	Q2	18 (18,19)	453 (438,463)	83 (80,86)	3.4 (3.3,3.5)	169 (165,175)	2.6 (2.4,2.7)	28 (27,29)	66 (60,72)	6 (5,6)	135 (129,146)	4 (4,5)	18 (18,19)
	Q3	18 (17,18)	441 (429,457)	82 (80,85)	3.3 (3.2,3.5)	170 (165,175)	2.6 (2.5,2.8)	28 (27,29)	62 (56,69)	5 (5,6)	132 (126,136)	4 (4,5)	19 (18,19)
	Q4	18 (18,19)	448 (432,459)	82 (79,86)	3.4 (3.3,3.5)	170 (164,177)	2.6 (2.5,2.7)	29 (28,30)	65 (59,76)	6 (5,6)	135 (130,141)	4 (4,4)	18 (17,18)
	Q5	19 (18,20)	449 (435,468)	80 (77,83)	3.3 (3.1,3.4)	165 (162,169)	2.6 (2.4,2.7)	29 (28,31)	65 (60,70)	6 (6,6)	139 (133,148)	4 (4,4)	17 (17,18)
	P trend	0.0002	0.61	0.18	0.49	0.17	0.81	0.002	0.29	0.003	0.26	<0.0001	0.001

Abbreviations: Q; quintile, 95% CI; 95% confidence intervals, LH; Luteinizing Hormone, FSH; Follicle stimulating hormone, SHBG; Sex Hormone Binding Globulin.

©2020 Nassan FL et al. *JAMA Network Open*.

eTable 6. Adjusted Median Differences in Semen Quality Parameters According to Adherence to Data-Derived Dietary Patterns

Reproductive Parameters		Semen volume, mL	Sperm concentration, million/mL	Total sperm count, million	Motile spermatozoa, %	Total motile sperm count, million	Progressively motile spermatozoa, %	Total progressively motile sperm count, million	Normal sperm morphology, %	Total normal morphologically sperm count, million
"Western" Pattern	Q1	Ref	Ref	Ref	Ref	Ref	Ref	Ref	Ref	Ref
	Q2	-0.1 (-0.3,0.0)	-1.8 (-6.9,3.4)	-10 (-26,5)	0.1 (-1.8,1.9)	-6 (-16,5)	-0.1 (-2.2,2.0)	-4 (-15,7)	0.2 (-0.5,0.8)	-1 (-3,0)
	Q3	-0.3 (-0.4,-0.1)	1.1 (-4.9,7.2)	-18 (-33,-3)	1.1 (-0.3,2.5)	-11 (-23,1)	0.2 (-2.0,2.3)	-10 (-21,2)	0.0 (-0.6,0.7)	-2 (-3,0)
	Q4	-0.3 (-0.4,-0.2)	-0.9 (-6.6,4.8)	-12 (-26,2)	-0.5 (-2.5,1.6)	-10 (-21,1)	-0.7 (-3.1,1.7)	-12 (-24,0)	-0.4 (-0.9,0.2)	-2 (-4,0)
	Q5	-0.3 (-0.5,-0.1)	-2.3 (-8.6,4.0)	-26 (-42,-9)	1.4 (-0.2,3.1)	-17 (-28,-5)	-0.3 (-2.6,1.9)	-15 (-27,-3)	-0.3 (-0.9,0.4)	-2 (-4,-1)
	P trend	0.002	0.42	0.005	0.32	0.05	0.44	0.02	0.10	0.02
"Prudent" Pattern	Q1	Ref	Ref	Ref	Ref	Ref	Ref	Ref	Ref	Ref
	Q2	0.0 (-0.1,0.2)	4.2 (-1.3,9.6)	9 (-7,25)	-1.0 (-3.1,1.1)	12 (-1,24)	-1.7 (-4.2,0.8)	9 (-1,20)	-0.3 (-0.9,0.3)	0 (-2,1)
	Q3	0.1 (-0.1,0.2)	3.3 (-1.8,8.4)	14 (-1,30)	-1.3 (-3.4,0.7)	10 (0,20)	-2.1 (-4.4,0.2)	8 (0,17)	0.2 (-0.5,0.8)	1 (-1,2)
	Q4	0.1 (-0.1,0.3)	6.7 (0.7,12.6)	27 (12,43)	-0.9 (-3.0,1.1)	19 (8,30)	-1.0 (-3.2,1.1)	18 (7,28)	0.4 (-0.3,1.1)	2 (1,4)
	Q5	0.2 (0.0,0.4)	6.7 (1.2,12.3)	43 (23,63)	-0.1 (-1.7,1.6)	30 (16,44)	0.1 (-2.3,2.4)	26 (14,37)	0.5 (-0.2,1.2)	2 (0,4)
	P trend	0.003	0.08	<0.0001	0.39	<0.0001	0.88	<0.0001	0.04	0.001
"Open-Sandwich" Pattern	Q1	Ref	Ref	Ref	Ref	Ref	Ref	Ref	Ref	Ref
	Q2	0 (-0.1,0.2)	-2.6 (-8.4,3.2)	-5 (-20,10)	-0.5 (-2.3,1.3)	-9 (-19,1)	0.0 (-2.0,1.9)	-8 (-17,2)	-0.4 (-1.0,0.2)	-1 (-2,1)
	Q3	-0.2 (-0.3,0)	0.1 (-5.8,5.9)	-5 (-21,11)	2.5 (0.6,4.5)	-6 (-18,6)	1.7 (-0.4,3.8)	-6 (-17,6)	0.1 (-0.5,0.7)	0 (-2,2)
	Q4	0 (-0.2,0.1)	-2.2 (-7.8,3.3)	-3 (-19,13)	2.1 (0.5,3.6)	-5 (-17,7)	-0.1 (-2.5,2.4)	-5 (-16,7)	0.1 (-0.5,0.7)	0 (-2,2)
	Q5	0 (-0.1,0.2)	-2.9 (-8.1,2.2)	-6 (-24,11)	2.3 (0.8,3.9)	-5 (-17,8)	1.9 (-0.3,4.2)	-6 (-18,5)	-0.1 (-0.8,0.6)	0 (-2,2)
	P trend	0.26	0.11	0.68	0.008	0.80	0.39	0.74	0.74	0.59
"Vegetarian-like" Pattern	Q1	Ref	Ref	Ref	Ref	Ref	Ref	Ref	Ref	Ref
	Q2	0 (-0.1,0.2)	-0.2 (-5.0,4.7)	4 (-9,17)	2.2 (0.0,4.5)	6 (-6,18)	1.7 (-0.8,4.1)	5 (-5,16)	0.7 (0.1,1.3)	1 (-1,3)
	Q3	0 (-0.1,0.2)	-0.9 (-5.9,4.2)	3 (-13,19)	2.3 (0.3,4.3)	5 (-7,17)	2.5 (0.2,4.8)	6 (-4,16)	0.2 (-0.4,0.7)	0 (-1,2)
	Q4	0 (-0.2,0.2)	5.9 (0.6,11.1)	11 (-4,26)	-0.5 (-2.6,1.6)	4 (-6,14)	0.4 (-1.9,2.6)	4 (-6,15)	0.0 (-0.6,0.6)	1 (-1,2)
	Q5	0.2 (0.0,0.3)	0.4 (-5.1,5.9)	6 (-11,24)	1.1 (-0.9,3.1)	7 (-3,17)	1.5 (-1.0,4.0)	9 (-1,19)	0.8 (0.2,1.4)	2 (0,3)
	P trend	0.18	0.53	0.29	0.95	0.22	0.84	0.30	0.04	0.18

Abbreviations: Q; quintile, 95% CI; 95% confidence intervals.
Models were adjusted for age, body mass index, height, smoking, use of marijuana and other recreational drug, moderate-to-vigorous physical activities (hours/ week), history of reproductive diseases, reproductive surgeries, and sexually transmitted diseases, season and calendar year of the sample, mother's education level, total energy intake, and abstinence time. Sperm motility models were further adjusted for time elapsed between specimen collection and analysis.

©2020 Nassan FL et al. *JAMA Network Open*.

eTable 7. Adjusted Probabilities of Semen Quality Parameters Below the WHO Lower Reference Limits According to Adherence to Data-Derived Dietary Patterns

Adjusted[1] proportions (95% CI) < WHO Lower Reference Limits per diet pattern quintiles							
Semen Quality Parameters	<1.5 mL Semen volume	<15 million/mL Sperm concentration	< 39 million Total sperm count	<40% Motile spermatozoa	<32% Progressively motile spermatozoa	< 4% Normal sperm morphology	Any semen parameter < WHO reference limits
"Western" Pattern							
Q1	4(3,6)	14(12,18)	12(9,15)	6(4,8)	8(6,10)	23(20,27)	37(33,41)
Q2	5(4,7)	16(14,20)	14(12,18)	6(4,8)	7(5,10)	24(20,27)	39(35,43)
Q3	6(4,8)	12(10,15)	11(9,14)	6(5,9)	7(5,10)	23(20,27)	36(32,40)
Q4	4(3,6)	16(13,19)	13(11,16)	5(4,7)	7(5,10)	24(21,28)	37(34,41)
Q5	6(4,9)	19(16,23)	17(14,20)	7(5,9)	8(6,11)	27(24,32)	45(41,49)
P trend	0.28	0.04	0.04	0.77	0.97	0.10	0.006
RR (95%CI) for Q5 compared to Q1	1.5(0.9,2.5)	1.3(1,1.7)	1.4(1.0,1.9)	1.1(0.7,1.8)	1.0(0.7,1.6)	1.2 (0.9,1.5)	1.2 (1.1,1.4)
"Prudent" Pattern							
Q1	7(5,10)	16(14,20)	14(11,17)	6(5,9)	7(5,10)	25(22,29)	41(37,45)
Q2	5(4,8)	18(15,21)	15(12,18)	8(6,11)	9(7,12)	25(22,29)	41(37,45)
Q3	5(3,7)	16(14,19)	14(12,17)	6(4,8)	7(5,10)	25(22,29)	41(38,46)
Q4	6(4,8)	13(10,16)	12(10,15)	5(4,7)	7(5,9)	25(21,29)	36(32,40)
Q5	3(2,5)	14(11,17)	12(9,15)	5(3,7)	7(5,9)	21(18,25)	34(30,38)
P trend	0.02	0.05	0.16	0.11	0.34	0.15	0.004
RR (95%CI) for Q5 compared to Q1	0.5(0.3,0.8)	0.9(0.6,1.1)	0.9(0.6,1.2)	0.8(0.5,1.3)	0.9(0.6,1.4)	0.9(0.7,1.1)	0.8(0.7,1.0)
"Open-Sandwich" Pattern							
Q1	6(4,9)	14(11,17)	12(10,15)	7(5,10)	8(6,11)	27(23,31)	40(36,44)
Q2	5(4,8)	16(14,20)	14(11,16)	6(4,8)	7(5,10)	27(24,31)	41(38,46)
Q3	6(4,8)	14(11,17)	14(12,17)	6(4,8)	8(6,10)	21(18,24)	36(32,40)
Q4	4(2,6)	17(14,21)	14(12,17)	6(5,9)	8(6,11)	23(19,26)	37(33,41)
Q5	5(4,7)	16(13,19)	13(10,16)	5(4,7)	6(5,9)	25(21,29)	39(35,43)
P trend	0.33	0.32	0.91	0.37	0.57	0.43	0.54
RR (95%CI) for Q5 compared to Q1	0.8(0.5,1.4)	1.2(0.9,1.6)	1.0(0.8,1.4)	0.7(0.4,1.2)	0.8 (0.5,1.3)	0.9(0.7,1.2)	1.0 (0.8,1.1)
"Vegetarian-like" Pattern							
Q1	5(4,8)	17(14,20)	15(12,18)	7(5,9)	8(6,11)	27(24,31)	41(37,45)
Q2	5(4,7)	15(12,18)	13(10,15)	4(3,6)	6(4,8)	22(19,25)	36(33,41)
Q3	5(4,7)	15(13,18)	13(11,16)	6(4,8)	7(5,9)	24(21,28)	38(34,42)
Q4	6(4,8)	14(12,17)	12(10,15)	8(6,10)	9(6,11)	28(24,32)	41(37,45)
Q5	5(3,7)	16(13,19)	14(11,17)	6(4,9)	8(6,11)	22(18,25)	36(33,41)
P trend	0.64	0.71	0.77	0.56	0.72	0.22	0.39
RR (95%CI) for Q5 compared to Q1	0.9(0.5,1.4)	1.0(0.7,1.2)	1.0(0.7,1.3)	1.0(0.6,1.5)	0.9(0.6,1.4)	0.8(0.7,1.0)	0.9(0.8,1.0)

©2020 Nassan FL et al. *JAMA Network Open*.

Abbreviations: Q; quintile, 95% CI; 95% confidence intervals, WHO; world health organization, RR; risk ratio. Risk ratio here is comparing between the Q5 and Q1.Models were adjusted for age, body mass index, height, smoking, use of marijuana and other recreational drug, moderate-to-vigorous physical activities (hours/ week), history of reproductive diseases, reproductive surgeries, and sexually transmitted diseases, season and calendar year of the sample, mother's education level, total energy intake, and abstinence time. Sperm motility models were further adjusted for time elapsed between specimen collection and analysis.

For assessing relative risks, we used log link function and binary distribution except for semen volume, motility, progressive motility, we used log link function and Poisson distribution due to non-convergence of the binary distribution.

©2020 Nassan FL et al. *JAMA Network Open*.

eTable 8. Adjusted Probabilities of Semen Quality Parameters Below the Damsgaard High Semen Quality Suggested Reference Limits According to Adherence to Data-Derived Dietary Patterns

Semen Quality Parameters	<40 million/mL Sperm concentration	<50% Motile spermatozoa	< 9% Normal sperm morphology	Any semen parameter < Damsgaard High Semen quality reference limits
"Western" Pattern				
Q1	42(38,46)	15(12,18)	65(61,69)	76(72,79)
Q2	46(42,50)	13(11,16)	63(59,67)	75(72,79)
Q3	42(38,46)	14(11,17)	63(60,67)	73(70,77)
Q4	44(40,48)	15(12,18)	69(65,73)	78(75,82)
Q5	46(42,51)	15(12,18)	68(64,73)	78(75,82)
P trend	0.14	0.79	0.05	0.14
RR (95%CI) for Q5 compared to Q1	1.1(1.0,1.3)	1.0 (0.7,1.3)	1.1(1.0,1.2)	1.0 (1.0,1.1)
"Prudent" Pattern				
Q1	47(43,51)	15(12,19)	67(63,71)	77(73,80)
Q2	45(41,49)	16(13,19)	67(63,71)	76(73,80)
Q3	45(41,49)	13(11,16)	68(64,71)	79(76,82)
Q4	42(38,46)	13(11,16)	65(61,69)	75(72,79)
Q5	41(37,46)	14(11,17)	63(59,67)	74(71,78)
P trend	0.02	0.38	0.11	0.18
RR (95%CI) for Q5 compared to Q1	0.9(0.8,1.0)	0.9(0.7,1.2)	0.9(0.9,1.0)	1.0 (0.9,1.0)
"Open-Sandwich" Pattern				
Q1	42(38,47)	16(13,19)	67(63,71)	76(73,80)
Q2	45(41,49)	16(13,19)	67(63,71)	76(73,80)
Q3	45(41,49)	15(12,18)	67(64,71)	78(74,81)
Q4	44(40,49)	14(12,17)	65(61,69)	77(73,80)
Q5	44(40,48)	12(9,15)	62(58,66)	75(71,78)
P trend	0.94	0.03	0.13	0.53
RR (95%CI) for Q5 compared to Q1	1.0(0.9,1.2)	0.7(0.5,1.0)	0.9(0.8,1.0)	1.0 (0.9,1.1)
"Vegetarian-like" Pattern				
Q1	46(42,50)	16(14,20)	69(66,73)	79(75,82)
Q2	45(41,49)	13(10,16)	63(60,67)	75(71,78)
Q3	45(41,49)	13(11,16)	67(63,71)	77(74,81)
Q4	41(37,45)	15(12,18)	69(65,73)	77(74,81)
Q5	43(39,48)	15(12,18)	61(57,65)	73(70,77)
P trend	0.35	0.77	0.04	0.09

©2020 Nassan FL et al. *JAMA Network Open*.

RR (95%CI) for Q5 compared to Q1	1.0 (0.8,1.1)	0.9(0.7,1.2)	0.9(0.8,1.0)	0.9(0.9,1.0)

Abbreviations: Q; quintile, 95% CI; 95% confidence intervals, RR; risk ratio. Risk ratio here is comparing between the Q5 and Q1.

Damsgaard reference limits: sperm concentration <40 mill/ml, motility <50%, and morphology <9%.

Models were adjusted for age, body mass index, height, smoking, use of marijuana and other recreational drug, moderate-to-vigorous physical activities (hours/ week), history of reproductive diseases, reproductive surgeries, and sexually transmitted diseases, season and calendar year of the sample, mother's education level, total energy intake, and abstinence time. Sperm motility models were further adjusted for time elapsed between specimen collection and analysis.

©2020 Nassan FL et al. *JAMA Network Open*.

eTable 9. Adjusted Median Differences in Semen Quality Parameters and Testicular Volume According to Adherence to Data-Derived Dietary Patterns, Additionally Adjusted for Use of Muscle-Enhancing Supplements

	Reproductive Parameters	Semen volume, mL	Sperm concentration, million/mL	Total sperm count, million	Motile spermatozoa, %	Total motile sperm count, million	Progressively motile spermatozoa, %	Total progressively motile sperm count, million	Normal sperm morphology, %	Total normal morphologically sperm count, million	Testicular volume by ultrasound, mean (mL)
"Western" Pattern	Q1	Ref	Ref	Ref	Ref	Ref	Ref	Ref	Ref	Ref	Ref
	Q2	-0.1 (-0.3,0)	-1.8 (-7.2,3.5)	-9.7 (-24.9,5.5)	-0.1 (-2,1.9)	-9 (-20,2)	-0.2 (-2.1,1.8)	-5 (-16,6)	0.1 (-0.6,0.8)	-1 (-3,0)	-0.7 (-1.2,-0.3)
	Q3	-0.3 (-0.4,-0.1)	0.4 (-5.7,6.6)	-18.3 (-33.9,-2.7)	0.9 (-0.7,2.6)	-11 (-24,1)	0.3 (-1.7,2.2)	-12 (-23,0)	-0.5 (-1,0.1)	-2 (-3,0)	-0.5 (-1,0)
	Q4	-0.3 (-0.5,-0.1)	-0.8 (-6.8,5.2)	-11.4 (-26.5,3.7)	-0.6 (-2.8,1.6)	-11 (-23,1)	-1.1 (-3.4,1.1)	-13 (-25,-1)	-0.4 (-1.1,0.3)	-2 (-3,0)	-0.4 (-1,0.1)
	Q5	-0.3 (-0.5,-0.1)	-3.3 (-9.8,3.2)	-26.1 (-44,-8.2)	1.1 (-0.7,2.9)	-18 (-31,-5)	-0.7 (-2.9,1.5)	-17 (-29,-5)	-0.4 (-1.1,0.2)	-3 (-5,-1)	-0.8 (-1.4,-0.3)
	P trend	0.002	0.33	0.01	0.39	0.04	0.22	0.01	0.06	0.00	0.06
"Prudent" Pattern	Q1	Ref	Ref	Ref	Ref	Ref	Ref	Ref	Ref	Ref	Ref
	Q2	0 (-0.1,0.2)	4.7 (-0.7,10.1)	8.7 (-6.5,23.9)	-0.8 (-2.9,1.4)	10 (-3,22)	-1.6 (-4,0.8)	9 (-2,19)	0.2 (-0.5,0.8)	0 (-1,2)	0.3 (-0.1,0.8)
	Q3	0.1 (-0.1,0.2)	3 (-2.2,8.2)	15.2 (-0.9,31.4)	-1.2 (-3.3,0.9)	11 (1,22)	-2 (-4.1,0.1)	8 (-1,17)	0.4 (-0.3,1.1)	1 (-1,2)	0.6 (0.1,1)
	Q4	0.1 (-0.1,0.3)	6.3 (0.4,12.3)	28.3 (12.9,43.8)	-0.7 (-2.8,1.5)	19 (7,31)	-0.6 (-2.6,1.4)	17 (8,27)	0.5 (-0.2,1.2)	2 (1,4)	0.2 (-0.3,0.6)
	Q5	0.2 (0,0.4)	6.9 (1.3,12.6)	43 (23,62.9)	0.2 (-1.6,2)	31 (17,46)	0.6 (-1.7,2.9)	25 (14,37)	-0.1 (-0.8,0.5)	2 (1,4)	0.3 (-0.2,0.9)
	P trend	0.031	0.06	<0.0001	0.40	<0.0001	0.42	<0.0001	0.04	0.000	0.45
"Open-Sandwich" Pattern	Q1	Ref	Ref	Ref	Ref	Ref	Ref	Ref	Ref	Ref	Ref
	Q2	0.1 (-0.1,0.2)	-2.9 (-8.6,2.8)	-4.5 (-19.7,10.7)	-0.7 (-2.7,1.3)	-8 (-20,3)	-0.3 (-2.2,1.5)	-7 (-16,2)	0.1 (-0.5,0.8)	0 (-2,1)	-0.5 (-0.9,-0.1)
	Q3	-0.1 (-0.2,0.1)	0 (-5.8,5.8)	-6.4 (-21.8,9.1)	2.4 (0.2,4.5)	-8 (-21,4)	1.5 (-0.5,3.5)	-7 (-18,4)	0.1 (-0.5,0.7)	0 (-2,1)	-0.4 (-0.8,0.1)
	Q4	0 (-0.1,0.2)	-2.7 (-8.1,2.8)	-3.4 (-19.6,12.7)	2 (0.2,3.8)	-6 (-19,6)	0 (-2.3,2.2)	-7 (-18,4)	0.1 (-0.6,0.8)	0 (-2,1)	-0.3 (-0.7,0.2)
	Q5	0.1 (-0.1,0.3)	-3.1 (-8.3,2.2)	-6.8 (-24.4,10.9)	2.2 (0.5,4)	-6 (-19,7)	1.7 (-0.5,3.8)	-6 (-17,5)	0.8 (0.2,1.4)	0 (-2,1)	-0.5 (-1,0)
	P trend	0.28	0.23	0.55	0.009	0.91	0.42	0.63	0.87	0.70	0.22
"V [illegible]	Q1	Ref	Ref	Ref	Ref	Ref	Ref	Ref	Ref	Ref	Ref

©2020 Nassan FL et al. *JAMA Network Open*.

	Q2	0.1 (-0.1,0.2)	-0.5 (-5.2,4.3)	4.9 (-9.1,18.9)	2 (-0.3,4.3)	6 (-6,19)	1.8 (-0.7,4.3)	6 (-4,16)	0.2 (-0.4,0.8)	1 (-1,2)	0 (-0.4,0.4)
	Q3	0 (-0.1,0.2)	-1 (-5.9,4)	2.2 (-13.6,18)	2.3 (0.3,4.3)	6 (-6,17)	2.6 (0.3,4.8)	6 (-4,16)	0.1 (-0.5,0.6)	0 (-2,1)	-0.3 (-0.7,0.2)
	Q4	0 (-0.2,0.2)	5.7 (0.4,11.1)	11.2 (-3.5,25.9)	-0.7 (-2.8,1.5)	6 (-6,17)	0.3 (-1.9,2.5)	4 (-6,15)	0.8 (0.2,1.4)	0 (-1,2)	0.1 (-0.4,0.5)
	Q5	0.2 (0,0.4)	-0.3 (-5.9,5.4)	6 (-11.9,24)	0.9 (-1.1,3)	5 (-6,16)	1.0 (-1.5,3.4)	8 (-2,18)	0.8 (0.2,1.4)	1 (0,3)	0.2 (-0.3,0.6)
	P trend	0.10	0.60	0.17	0.60	0.23	0.59	0.27	0.08	0.30	0.25

Abbreviations: Q; quintile, Ref; reference.

12 men were missing data on the muscle enhancing supplements.Models were adjusted for age, body mass index, height, smoking, use of marijuana and other recreational drug, moderate-to-vigorous physical activities (hours/ week), history of reproductive diseases, reproductive surgeries, and sexually transmitted diseases, season and calendar year of the sample, mother's education level, total energy intake, abstinence time, and use of muscle enhancing supplements. Sperm motility models were further adjusted for time elapsed between specimen collection and analysis.

©2020 Nassan FL et al. *JAMA Network Open*.

eTable 10. Adjusted Median Differences in Serum Reproductive Hormone Concentrations on the Scale for the Individual Hormones as Stated in the Table According to Adherence to Data-Derived Dietary Patterns, Additionally Adjusted for Use of Muscle-Enhancing Supplements

Reproductive Parameters			Total Testosterone, nmol/L	Free Testosterone, pmol/L	Estradiol, pg/mL	LH, IU/L	Inhibin-B, pg/mL	FSH, IU/L	SHBG, nmol/L	Inhibin-B/ FSH	Total Testosterone/ LH	Free Testosterone/ LH	Estradiol/ Total Testosterone	(Estradiol/ Free Testosterone)*100
"Western" Pattern		Q1	Ref	Ref	Ref	Ref	Ref	Ref	Ref	Ref	Ref	Ref	Ref	Ref
		Q2	0.1 (-0.5,0.8)	10 (-6,26)	0 (-3,3)	0.1 (-0.1,0.3)	-3 (-10,4)	0.1 (-0.1,0.3)	0.3 (-1.3,1.8)	-7 (-15,1)	-0.1 (-0.4,0.2)	3 (-5,11)	-0.1 (-0.3,0.1)	-0.5 (-1.4,0.3)
		Q3	0.5 (-0.2,1.3)	22 (4,41)	2 (-2,5)	0 (-0.2,0.2)	8 (0,15)	0.1 (-0.1,0.3)	-0.5 (-2.1,1)	-3 (-12,5)	0.1 (-0.3,0.4)	6 (-3,14)	0 (-0.2,0.2)	-0.5 (-1.4,0.4)
		Q4	0.8 (0.1,1.5)	28 (10,46)	1 (-3,4)	0.2 (0,0.4)	-4 (-12,3)	0.2 (0,0.4)	0.1 (-1.6,1.7)	-12 (-19,-4)	0 (-0.3,0.4)	3 (-7,12)	0 (-0.2,0.2)	-0.9 (-1.9,0)
		Q5	0.9 (0,1.7)	47 (27,67)	4 (1,7)	0.2 (0,0.4)	-10 (-17,-3)	0.2 (0,0.5)	-0.7 (-2.3,1)	-14 (-23,-6)	0.2 (-0.2,0.5)	11 (2,19)	0 (-0.2,0.2)	-1 (-1.9,-0.2)
		P trend	0.02	<0.0001	0.08	0.15	0.01	0.05	0.32	0.001	0.66	0.007	0.63	0.010
"Prudent" Pattern		Q1	Ref	Ref	Ref	Ref	Ref	Ref	Ref	Ref	Ref	Ref	Ref	Ref
		Q2	-0.2 (-0.9,0.5)	5 (-14,24)	-2 (-5,1)	0 (-0.2,0.2)	2 (-5,8)	0.1 (-0.1,0.3)	0.4 (-1.1,1.9)	-2 (-9,5)	-0.1 (-0.4,0.3)	1 (-7,9)	0 (-0.2,0.2)	-0.4 (-1.2,0.4)
		Q3	0.1 (-0.6,0.9)	-1 (-20,19)	-3 (-6,-1)	-0.2 (-0.4,0)	11 (3,18)	-0.2 (-0.4,0)	0.4 (-1.1,1.9)	8 (1,15)	0.3 (0,0.6)	7 (-2,15)	-0.1 (-0.3,0)	-0.4 (-1.1,0.3)
		Q4	-0.1 (-0.8,0.7)	1 (-18,20)	-5 (-8,-2)	-0.1 (-0.3,0.1)	1 (-6,8)	0.1 (-0.1,0.3)	0 (-1.5,1.4)	-1 (-8,5)	0.1 (-0.3,0.5)	0 (-8,8)	-0.1 (-0.3,0.1)	-0.4 (-1.2,0.4)
		Q5	-0.1 (-0.8,0.7)	-9 (-28,10)	-5 (-9,-2)	0 (-0.3,0.2)	4 (-3,11)	0.1 (-0.1,0.3)	1.5 (-0.1,3.1)	-3 (-9,3)	0.1 (-0.3,0.5)	5 (-3,13)	-0.1 (-0.3,0)	-0.3 (-1.2,0.5)
		P trend	0.98	0.27	0.001	0.52	0.74	0.59	0.07	0.47	0.66	0.51	0.14	0.58
"Open-Sandwich" Pattern		Q1	Ref	Ref	Ref	Ref	Ref	Ref	Ref	Ref	Ref	Ref	Ref	Ref
		Q2	0.2 (-0.5,1)	10 (-9,28)	2 (-1,6)	0.1 (-0.1,0.3)	-1 (-9,7)	0.1 (-0.1,0.3)	0.2 (-1.2,1.6)	0 (-7,6)	0.1 (-0.3,0.4)	2 (-6,11)	0 (-0.2,0.2)	0.3 (-0.5,1.1)
		Q3	0 (-0.8,0.7)	4 (-13,22)	0 (-3,4)	0.1 (-0.1,0.3)	0 (-9,8)	0 (-0.1,0.2)	0.6 (-0.9,2.1)	1 (-6,9)	-0.2 (-0.6,0.2)	-5 (-13,3)	0.1 (-0.1,0.4)	0.3 (-0.5,1.1)
		Q4	0.5 (-0.3,1.3)	-6 (-26,15)	4 (1,8)	0.3 (0.1,0.5)	1 (-7,8)	0 (-0.2,0.3)	1.3 (-0.2,2.7)	0 (-7,8)	-0.2 (-0.6,0.1)	-9 (-17,-1)	0.1 (-0.1,0.3)	0.7 (-0.2,1.6)
		Q5	0.6 (-0.2,1.5)	1 (-19,21)	4 (0,7)	0.2 (0,0.4)	-8 (-15,0)	0.1 (-0.1,0.3)	1.3 (-0.3,3)	-6 (-13,1)	-0.3 (-0.7,0)	-8 (-16,0)	-0.1 (-0.3,0.2)	0.5 (-0.3,1.4)
		P trend	0.18	0.46	0.05	0.04	0.04	0.30	0.07	0.13	0.03	0.02	0.19	0.57
"V eg		Q1	Ref	Ref	Ref	Ref	Ref	Ref	Ref	Ref	Ref	Ref	Ref	Ref

©2020 Nassan FL et al. *JAMA Network Open*.

		Q2	0.7 (0,1.5)	5 (-11,22)	2 (-1,6)	0 (-0.2,0.3)	-4 (-11,3)	0.1 (-0.1,0.3)	0.5 (-0.9,1.9)	-8 (-14,-1)	0.4 (0.1,0.8)	8 (1,16)	-0.1 (-0.3,0.1)	-0.2 (-1.1,0.6)
		Q3	-0.1 (-0.7,0.6)	4 (-13,21)	1 (-2,4)	0 (-0.2,0.2)	-3 (-11,4)	0.2 (0,0.5)	0.4 (-0.9,1.7)	-9 (-16,-2)	0 (-0.3,0.3)	0 (-7,7)	0 (-0.2,0.2)	0.3 (-0.6,1.2)
		Q4	0.9 (0.2,1.7)	5 (-11,21)	2 (-2,5)	0.1 (-0.2,0.3)	-4 (-11,4)	0.1 (-0.1,0.3)	1.4 (0,2.8)	-6 (-14,1)	0.4 (0,0.7)	4 (-3,12)	-0.2 (-0.4,0)	-0.5 (-1.3,0.4)
		Q5	0.8 (0.1,1.5)	10 (-8,28)	-1 (-4,3)	-0.1 (-0.3,0.1)	-10 (-17,-3)	0.1 (-0.2,0.3)	1.7 (0.2,3.3)	-8 (-15,-2)	0.5 (0.1,0.8)	8 (0,16)	-0.2 (-0.3,0)	-0.8 (-1.6,0)
		P trend	0.10	0.35	0.39	0.35	0.015	0.94	0.02	0.08	0.06	0.36	0.05	0.04

Abbreviations: Q; quintile, LH; Luteinizing Hormone, FSH; Follicle stimulating hormone, SHBG; Sex Hormone Binding Globulin, Ref; reference.
13 men were missing data on the muscle enhancing supplements.

Models were adjusted for age, body mass index, height, smoking, use of marijuana and other recreational drug, moderate-to-vigorous physical activities (hours/ week), history of reproductive diseases, reproductive surgeries, and sexually transmitted diseases, season and calendar year of the sample, mother’s education level, total energy intake, time of the day of the sample collection, and use of muscle enhancing supplements n.

©2020 Nassan FL et al. *JAMA Network Open*.

eTable 11. Adjusted Median Differences in Semen Quality Parameters and Testicular Volume According to Adherence to Data-Derived Dietary Patterns, Without Adjustment for Body Mass Index

	Reproductive Parameters	Semen volume, mL	Sperm concentration, million/mL	Total sperm count, million	Motile spermatozoa, %	Total motile sperm count, million	Progressively motile spermatozoa, %	Total progressively motile sperm count, million	Normal sperm morphology, %	Total normal morphologically sperm count, million	Testicular volume by ultrasound, mean (mL)
"Western" Pattern	Q1	Ref	Ref	Ref	Ref	Ref	Ref	Ref	Ref	Ref	Ref
	Q2	-0.2 (-0.3,0)	-2 (-7.3,3.2)	-11 (-26,4)	-0.1 (-2.0,1.8)	-6 (-18,6)	0.1 (-2.1,2.2)	-4 (-14,6)	0.2 (-0.5,0.8)	-1 (-3,1)	-0.7 (-1.2,-0.2)
	Q3	-0.3 (-0.5,-0.1)	-0.4 (-6.1,5.3)	-19 (-34,-3)	1.0 (-0.6,2.5)	-11 (-24,2)	0.3 (-1.8,2.4)	-10 (-21,1)	0.1 (-0.5,0.8)	-2 (-3,0)	-0.5 (-1,0)
	Q4	-0.3 (-0.4,-0.1)	-1.7 (-7.4,4)	-12 (-26,2)	-0.8 (-2.8,1.3)	-10 (-23,3)	-0.8 (-3.2,1.6)	-10 (-21,2)	-0.3 (-0.9,0.2)	-2 (-4,0)	-0.5 (-1,0.1)
	Q5	-0.3 (-0.5,-0.2)	-2.8 (-9.1,3.4)	-27 (-43,-11)	1.1 (-0.7,2.9)	-16 (-30,-2)	-0.1 (-2.4,2.2)	-14 (-26,-2)	-0.2 (-0.9,0.5)	-3 (-4,-1)	-0.9 (-1.5,-0.3)
	P trend	0.001	0.34	0.009	0.47	0.10	0.38	0.03	0.15	0.01	0.06
"Prudent" Pattern	Q1	Ref	Ref	Ref	Ref	Ref	Ref	Ref	Ref	Ref	Ref
	Q2	0 (-0.2,0.2)	4.1 (-1.3,9.5)	7 (-8,22)	-1.1 (-3.2,1)	10 (-2,23)	-1.6 (-4,0.7)	9 (-2,19)	-0.4 (-1,0.3)	0 (-2,1)	0.2 (-0.3,0.6)
	Q3	0.1 (-0.1,0.2)	3.1 (-1.9,8.2)	13 (-2,27)	-1.7 (-3.7,0.3)	10 (-2,21)	-2.1 (-4.4,0.1)	8 (-1,17)	0.2 (-0.5,0.9)	1 (-1,2)	0.6 (0.1,1.1)
	Q4	0.1 (0,0.3)	6.3 (0.3,12.2)	24 (10,39)	-1.1 (-3.1,1)	20 (8,32)	-0.9 (-3,1.2)	17 (8,27)	0.4 (-0.3,1.1)	2 (1,4)	0.2 (-0.2,0.7)
	Q5	0.2 (0,0.4)	6.5 (1.1,11.9)	39 (20,58)	-0.3 (-1.9,1.4)	28 (13,43)	0 (-2.3,2.2)	25 (12,37)	0.5 (-0.3,1.2)	2 (0,4)	0.3 (-0.2,0.9)
	P trend	0.008	0.05	<0.0001	0.56	<0.0001	0.72	<0.0001	0.02	0.001	0.12
"Open-Sandwich" Pattern	Q1	Ref	Ref	Ref	Ref	Ref	Ref	Ref	Ref	Ref	Ref
	Q2	0 (-0.2,0.2)	-3.1 (-9,2.7)	-5 (-19,9)	-0.5 (-2.4,1.4)	-8 (-19,3)	-0.4 (-2.2,1.5)	-6 (-15,3)	-0.2 (-0.9,0.4)	0 (-2,1)	-0.5 (-0.9,0)
	Q3	-0.2 (-0.3,0)	0.4 (-5.3,6.2)	-6 (-20,9)	2.7 (0.6,4.7)	-3 (-16,10)	1.5 (-0.5,3.6)	-5 (-16,7)	0.1 (-0.5,0.7)	0 (-2,2)	-0.4 (-0.9,0.1)
	Q4	0 (-0.2,0.1)	-2.7 (-8,2.7)	-2 (-18,14)	2.2 (0.5,3.8)	-6 (-19,8)	-0.1 (-2.3,2.2)	-3 (-14,8)	0.2 (-0.5,0.8)	0 (-2,2)	-0.3 (-0.8,0.2)
	Q5	0 (-0.1,0.2)	-3.9 (-8.9,1.1)	-5 (-21,11)	2.3 (0.7,3.9)	-4 (-17,10)	1.7 (-0.6,3.9)	-3 (-14,8)	-0.1 (-0.8,0.7)	0 (-2,2)	-0.4 (-0.9,0.1)
	P trend	0.21	0.17	0.69	0.012	0.93	0.40	0.80	0.49	0.65	0.29
"Vegetarian-like" Pattern	Q1	Ref	Ref	Ref	Ref	Ref	Ref	Ref	Ref	Ref	Ref
	Q2	0.1 (-0.1,0.2)	-0.2 (-4.9,4.5)	5 (-9,19)	1.9 (-0.4,4.2)	4 (-9,17)	2 (-0.4,4.5)	5 (-5,15)	0.5 (-0.1,1.2)	1 (-1,2)	0 (-0.5,0.4)
	Q3	0 (-0.1,0.2)	-0.6 (-5.6,4.3)	6 (-9,21)	2 (0,4)	4 (-9,17)	2.9 (0.8,5.1)	7 (-3,17)	0.1 (-0.5,0.7)	0 (-2,1)	-0.3 (-0.9,0.2)
	Q4	0 (-0.2,0.2)	5.8 (0.6,11)	12 (-2,27)	-0.7 (-2.7,1.3)	1 (-10,12)	0.7 (-1.4,2.9)	3 (-6,13)	0 (-0.6,0.6)	0 (-1,2)	0 (-0.5,0.5)
	Q5	0.2 (0,0.3)	0.8 (-4.6,6.2)	6 (-11,23)	0.8 (-1.1,2.8)	5 (-7,16)	1.7 (-0.6,4)	8 (-2,18)	0.8 (0.2,1.4)	1 (0,3)	0.1 (-0.4,0.5)
	P trend	0.18	0.37	0.12	0.88	0.23	0.70	0.27	0.08	0.18	0.39

Abbreviations: Q; quintile, Ref; reference.

Models were adjusted for age, height, smoking, use of marijuana and other recreational drug, moderate-to-vigorous physical activities (hours/ week), history of reproductive diseases, reproductive surgeries, and sexually transmitted diseases, season and calendar year of the sample, mother's education level, total energy intake, and abstinence time. Sperm motility models were further adjusted for time elapsed between specimen collection and analysis.

©2020 Nassan FL et al. *JAMA Network Open*.

eTable 12. Adjusted Median Differences in Serum Reproductive Hormone Concentrations on the Scale for the Individual Hormones as Stated in the Table According to Adherence to Data-Derived Dietary Patterns, Without Adjustment for Body Mass Index

	Reproductive Parameters	Total Testosterone, nmol/L	Free Testosterone, pmol/L	Estradiol, pg/mL	LH, IU/L	Inhibin-B, pg/mL	FSH, IU/L	SHBG, nmol/L	Inhibin-B/ FSH	Total Testosterone/ LH	Free Testosterone/ LH	Estradiol/ Total Testosterone	(Estradiol/ Free Testosterone) *100
"Western" Pattern	Q1	Ref	Ref	Ref	Ref	Ref	Ref	Ref	Ref	Ref	Ref	Ref	Ref
	Q2	0.3 (-0.3,1)	12 (-4,29)	1 (-3,4)	0.1 (0.0,0.3)	-6 (-14,1)	0.1 (-0.1,0.4)	0.7 (-0.8,2.2)	-6 (-14,3)	-0.1 (-0.5,0.2)	3 (-5,12)	-0.1 (-0.3,0.1)	-0.1 (-1,0.8)
	Q3	0.6 (-0.1,1.4)	23 (5,42)	3 (-1,7)	0 (-0.1,0.2)	4 (-5,12)	0.1 (-0.1,0.4)	-0.7 (-2.1,0.7)	-1 (-9,8)	0 (-0.4,0.3)	6 (-2,15)	-0.1 (-0.3,0.2)	-0.3 (-1.2,0.6)
	Q4	0.9 (0.2,1.6)	30 (12,49)	2 (-2,6)	0.1 (-0.1,0.3)	-3 (-12,5)	0.3 (0.0,0.5)	1 (-0.5,2.5)	-11 (-19,-3)	0 (-0.4,0.3)	5 (-5,14)	-0.1 (-0.3,0.1)	-0.9 (-1.8,0)
	Q5	1 (0.1,1.8)	47 (28,67)	4 (1,8)	0.2 (0,0.4)	-12 (-20,-4)	0.2 (0.0,0.5)	-1.2 (-2.6,0.3)	-12 (-21,-3)	0 (-0.4,0.3)	11 (2,19)	-0.1 (-0.3,0.1)	-0.9 (-1.8,0)
	P trend	0.029	<0.0001	0.08	0.13	0.006	0.10	0.08	0.007	0.54	0.01	0.36	0.018
"Prudent" Pattern	Q1	Ref	Ref	Ref	Ref	Ref	Ref	Ref	Ref	Ref	Ref	Ref	Ref
	Q2	-0.3 (-1.1,0.4)	5 (-14,25)	-3 (-6,1)	0 (-0.2,0.2)	1 (-6,8)	0.1 (-0.1,0.3)	0.3 (-1.1,1.8)	-4 (-11,3)	-0.1 (-0.4,0.2)	1 (-7,9)	0 (-0.2,0.2)	0 (-0.8,0.8)
	Q3	0.2 (-0.6,0.9)	-2 (-22,18)	-3 (-6,0)	-0.2 (-0.4,0)	8 (0,16)	-0.2 (-0.4,0.0)	0.4 (-1,1.7)	7 (0,14)	0.3 (-0.1,0.6)	7 (-2,16)	-0.1 (-0.3,0.1)	-0.1 (-0.8,0.6)
	Q4	-0.2 (-1,0.5)	1 (-18,20)	-4 (-8,-1)	0 (-0.2,0.2)	1 (-7,8)	0.1 (-0.1,0.3)	-0.4 (-1.9,1.1)	-5 (-12,1)	0 (-0.4,0.3)	0 (-9,9)	-0.1 (-0.2,0.1)	-0.2 (-1,0.7)
	Q5	-0.2 (-1,0.5)	-10 (-29,9)	-5 (-9,-1)	-0.1 (-0.3,0.2)	0 (-8,7)	0.1 (-0.1,0.4)	1.4 (-0.1,2.9)	-6 (-13,1)	0.1 (-0.3,0.4)	5 (-3,14)	-0.1 (-0.3,0.1)	0 (-0.9,0.8)
	P trend	0.97	0.20	0.001	0.69	0.89	0.43	0.39	0.22	0.81	0.64	0.52	0.95
"Open-Sandwich" Pattern	Q1	Ref	Ref	Ref	Ref	Ref	Ref	Ref	Ref	Ref	Ref	Ref	Ref
	Q2	0.1 (-0.6,0.9)	7 (-11,26)	3 (-1,7)	0.1 (-0.1,0.3)	-6 (-13,2)	0.1 (-0.1,0.3)	-0.3 (-1.5,0.8)	0 (-7,7)	0 (-0.3,0.4)	2 (-7,11)	0 (-0.2,0.2)	0.3 (-0.5,1.1)
	Q3	-0.1 (-0.8,0.7)	4 (-15,23)	1 (-2,5)	0.1 (-0.1,0.2)	-2 (-10,6)	0.0 (-0.2,0.2)	0 (-1.4,1.5)	0 (-8,8)	-0.2 (-0.5,0.2)	-5 (-14,3)	0 (-0.2,0.2)	0.3 (-0.5,1.2)
	Q4	0.5 (-0.3,1.3)	-8 (-29,13)	5 (1,8)	0.3 (0.1,0.4)	-3 (-11,4)	0.1 (-0.2,0.3)	1.4 (0.1,2.8)	0 (-8,8)	-0.2 (-0.5,0.2)	-9 (-19,0)	0 (-0.2,0.2)	0.7 (-0.2,1.7)
	Q5	0.4 (-0.5,1.3)	-4 (-24,17)	4 (0,8)	0.2 (0.1,0.4)	-11 (-19,-3)	0.1 (-0.1,0.4)	0.5 (-1.1,2.2)	-6 (-14,2)	-0.2 (-0.6,0.1)	-6 (-15,4)	-0.2 (-0.4,0)	0.6 (-0.3,1.5)
	P trend	0.14	0.42	0.08	0.01	0.02	0.41	0.24	0.11	0.11	0.04	0.13	0.37
"Vegetarian-like"	Q1	Ref	Ref	Ref	Ref	Ref	Ref	Ref	Ref	Ref	Ref	Ref	Ref
	Q2	0.8 (0.1,1.4)	8 (-10,25)	1 (-2,5)	0 (-0.2,0.2)	-1 (-9,6)	0.1 (-0.1,0.3)	1.3 (0.0,2.6)	-7 (-13,0)	0.3 (0,0.7)	8 (0,17)	-0.1 (-0.3,0.1)	-0.5 (-1.3,0.4)

©2020 Nassan FL et al. *JAMA Network Open*.

	Q3	0 (-0.7,0.7)	4 (-12,21)	1 (-3,4)	-0.1 (-0.3,0.1)	0 (-8,9)	0.3 (0.0,0.5)	1.3 (-0.1,2.7)	-8 (-15,0)	0.1 (-0.2,0.4)	1 (-6,9)	-0.1 (-0.3,0.1)	0.2 (-0.7,1.1)
	Q4	0.8 (0,1.5)	5 (-12,21)	0 (-4,4)	0 (-0.2,0.3)	-1 (-9,8)	0.1 (-0.1,0.3)	2.3 (1.1,3.6)	-5 (-13,3)	0.4 (0.1,0.7)	5 (-2,12)	-0.3 (-0.5,-0.1)	-0.6 (-1.4,0.2)
	Q5	1 (0.2,1.7)	12 (-7,30)	-2 (-6,2)	-0.1 (-0.4,0.1)	-4 (-11,4)	0.1 (-0.2,0.3)	2.8 (1.3,4.3)	-6 (-13,1)	0.5 (0.2,0.9)	9 (1,18)	-0.3 (-0.5,-0.1)	-1.2 (-2,-0.4)
	P trend	0.03	0.24	0.09	0.18	0.046	0.94	0.0009	0.18	0.001	0.21	0.0004	0.004

Abbreviations: Q; quintile, 95% CI; 95% confidence intervals, LH; Luteinizing Hormone, FSH; Follicle stimulating hormone, SHBG; Sex Hormone Binding Globulin, Ref; reference. Models were adjusted for age, height, smoking, use of marijuana and other recreational drug, moderate-to-vigorous physical activities (hours/ week), history of reproductive diseases, reproductive surgeries, and sexually transmitted diseases, season and calendar year of the sample, mother's education level, total energy intake, and time of the day of the sample collection.

©2020 Nassan FL et al. *JAMA Network Open*.

eTable 13. Adjusted Median Differences in Semen Quality Parameters and Testicular Volume According to Adherence to Data-Derived Dietary Patterns Among 2169 Men Who Did Not Report Muscle-Enhancing Supplements

Adjusted median difference for men who did not report muscle-enhancing supplements among 2169 men

	Reproductive Parameters	Semen volume, mL	Sperm concentration, million/mL	Total sperm count, million	Motile spermatozoa, %	Total motile sperm count, million	Progressively motile spermatozoa, %	Total progressively motile sperm count, million	Normal sperm morphology, %	Total normal morphologically sperm count, million	Testicular volume by ultrasound, mean (mL)
	Q1	Ref	Ref	Ref	Ref	Ref	Ref	Ref	Ref	Ref	Ref
“Western” Pattern	Q2	-0.2 (-0.4,0)	-0.8 (-7.2,5.6)	1 (-18,20)	0.9 (-1.4,3.1)	-2 (-16,11)	0.6 (-1.8,3)	-1 (-13,12)	0.1 (-0.7,0.8)	0 (-2,2)	-0.7 (-1.3,-0.1)
	Q3	-0.2 (-0.4,0)	0.9 (-6.5,8.3)	-12 (-32,9)	0.7 (-1,2.5)	-10 (-25,4)	-0.6 (-3,1.8)	-11 (-22,0)	0.1 (-0.7,0.9)	-1 (-3,1)	-0.6 (-1.2,0)
	Q4	-0.3 (-0.5,-0.1)	-0.5 (-7.5,6.5)	-1 (-21,19)	0.7 (-1.6,3.1)	-7 (-23,8)	-0.5 (-3.3,2.2)	-10 (-22,3)	-0.6 (-1.2,0)	-1 (-3,1)	-0.4 (-1.1,0.2)
	Q5	-0.3 (-0.5,0)	-2.6 (-9.7,4.5)	-17 (-37,4)	1.5 (-0.4,3.5)	-13 (-28,2)	-0.4 (-3,2.2)	-12 (-25,0)	-0.2 (-0.9,0.5)	-2 (-4,0)	-0.9 (-1.6,-0.3)
	P trend	0.03	0.37	0.10	0.27	0.09	0.68	0.11	0.16	0.04	0.09
	Q1	Ref	Ref	Ref	Ref	Ref	Ref	Ref	Ref	Ref	Ref
“Prudent” Pattern	Q2	0.1 (-0.1,0.3)	4.1 (-2.2,10.5)	14 (-4,33)	-0.7 (-3.1,1.8)	11 (-3,25)	-1.1 (-3.8,1.6)	10 (-2,21)	-0.5 (-1.2,0.2)	0 (-1,2)	0.3 (-0.3,0.8)
	Q3	0.2 (0,0.3)	3.7 (-3,10.4)	18 (-2,38)	-0.5 (-2.6,1.6)	13 (1,25)	-1.3 (-3.7,1)	10 (0,19)	0.2 (-0.5,0.9)	2 (0,3)	0.6 (0.0,1.1)
	Q4	0.1 (-0.1,0.2)	7.2 (0.1,14.3)	27 (8,46)	0.6 (-1.7,2.9)	20 (8,32)	0.8 (-1.7,3.4)	17 (5,29)	0.5 (-0.2,1.2)	3 (1,5)	0.1 (-0.5,0.7)
	Q5	0.3 (0.1,0.5)	7.8 (0.5,15.1)	50 (28,72)	1.6 (-0.3,3.6)	36 (19,53)	1.7 (-1,4.4)	31 (16,45)	0.7 (0,1.3)	3 (1,5)	0.5 (-0.1,1.2)
	P trend	0.15	0.03	<0.0001	0.10	<0.0001	0.15	0.0002	0.01	0.0003	0.12
	Q1	Ref	Ref	Ref	Ref	Ref	Ref	Ref	Ref	Ref	Ref
“Open-Sandwich” Pattern	Q2	0 (-0.2,0.2)	-0.9 (-7.1,5.3)	-5 (-23,14)	0.5 (-1.5,2.5)	-5 (-18,8)	0.9 (-1.1,2.9)	-2 (-13,9)	0 (-0.7,0.6)	0 (-2,1)	-0.4 (-0.9,0.1)
	Q3	-0.1 (-0.3,0.1)	1.3 (-5.1,7.7)	-6 (-25,13)	2.4 (0.3,4.5)	-4 (-18,10)	1.6 (-0.7,4)	-2 (-14,9)	0.2 (-0.4,0.7)	0 (-2,2)	-0.3 (-0.8,0.2)
	Q4	0.1 (-0.2,0.3)	-0.5 (-6.5,5.5)	-2 (-22,18)	2.7 (0.7,4.7)	-1 (-15,14)	1 (-1.6,3.6)	-2 (-13,10)	0.3 (-0.2,0.9)	0 (-2,2)	-0.3 (-0.9,0.2)
	Q5	0.1 (-0.1,0.4)	-1.2 (-7.5,5)	-1 (-24,22)	2.9 (0.9,4.9)	-1 (-17,15)	2.3 (0,4.5)	1 (-13,15)	0.1 (-0.7,0.8)	0 (-3,2)	-0.2 (-0.9,0.5)
	P trend	0.11	0.20	0.68	0.006	0.76	0.17	0.99	0.84	0.80	0.61
	Q1	Ref	Ref	Ref	Ref	Ref	Ref	Ref	Ref	Ref	Ref
“V eg eta	Q2	0.2 (0,0.3)	2.9 (-3,8.9)	16 (-3,35)	1.8 (-0.7,4.4)	10 (-4,23)	1.4 (-1.9,4.7)	11 (-2,24)	1 (0.3,1.7)	1 (-1,3)	0.3 (-0.3,0.8)

©2020 Nassan FL et al. *JAMA Network Open*.

	Q3	0 (-0.2,0.2)	2.4 (-4.1,8.8)	8 (-13,30)	2.5 (0,5)	8 (-7,23)	3.2 (0,6.3)	9 (-4,21)	0.4 (-0.2,1)	0 (-1,2)	-0.2 (-0.8,0.4)
	Q4	0.1 (-0.1,0.3)	8.8 (2,15.6)	19 (0,37)	-0.8 (-3.4,1.8)	9 (-2,21)	0.6 (-2.3,3.5)	9 (-1,20)	0.2 (-0.3,0.8)	1 (-1,2)	0.4 (-0.2,1)
	Q5	0.2 (0,0.4)	1.7 (-5.1,8.4)	12 (-9,34)	1.2 (-1.3,3.6)	7 (-5,19)	1.5 (-1.7,4.6)	8 (-1,18)	1.2 (0.6,1.9)	2 (0,4)	0.3 (-0.2,0.9)
	P trend	0.07	0.60	0.47	0.92	0.39	0.90	0.32	0.01	0.04	0.06

Abbreviations: 95% CI; 95% confidence intervals.

Models were adjusted for age, body mass index, height, smoking, use of marijuana and other recreational drug, moderate-to-vigorous physical activities (hours/ week), history of reproductive diseases, reproductive surgeries, and sexually transmitted diseases, season and calendar year of the sample, mother's education level, total energy intake, and abstinence time. Sperm motility models were further adjusted for time elapsed between specimen collection and analysis.

* Tests for trend were conducted across quintiles using the median value in each quintile of the diet patterns as a continuous variable in the regression models.

©2020 Nassan FL et al. *JAMA Network Open*.

eTable 14. Adjusted Median Differences in Serum Reproductive Hormone Concentrations on the Scale for the Individual Hormones as Stated in the Table According to Adherence to Data-Derived Dietary Patterns Among 2169 Men Who Did Not Report Muscle-Enhancing Supplements

Adjusted median difference for men who did not report muscle-enhancing supplements **among 2169 men**

	Reproductive Parameters	Total Testosterone, nmol/L	Free Testosterone, pmol/L	Estradiol, pg/mL	Inhibin-B, pg/mL	LH, IU/L	FSH, IU/L	SHBG, nmol/L	Inhibin-B/ FSH	Total Testosterone/ LH	Free Testosterone/ LH	Estradiol/ Total Testosterone	(Estradiol/ Free Testosterone)*100
	Q1	Ref	Ref	Ref	Ref	Ref	Ref	Ref	Ref	Ref	Ref	Ref	Ref
"Western" Pattern	Q2	0.1 (-0.7,1)	-5 (-25,14)	1 (-3,5)	-2 (-11,7)	0 (-0.2,0.2)	0.1 (-0.2,0.3)	0.6 (-1.2,2.4)	-5 (-16,6)	-0.1 (-0.4,0.3)	1 (-9,11)	0 (-0.2,0.3)	0.4 (-0.6,1.4)
	Q3	0.5 (-0.2,1.3)	8 (-12,28)	4 (1,8)	2 (-7,12)	0 (-0.3,0.2)	0.1 (-0.2,0.4)	-0.1 (-2.1,1.9)	-4 (-15,8)	0.1 (-0.3,0.5)	6 (-4,17)	0.1 (-0.2,0.3)	0.5 (-0.6,1.5)
	Q4	0.5 (-0.4,1.5)	16 (-7,39)	3 (-1,7)	-5 (-15,5)	0 (-0.3,0.3)	0.2 (0,0.4)	0.1 (-1.8,2.1)	-12 (-22,-1)	0 (-0.3,0.4)	3 (-9,15)	0.1 (-0.1,0.3)	-0.2 (-1.4,0.9)
	Q5	-0.4 (-1.1,0.4)	42 (19,65)	5 (1,8)	-13 (-22,-4)	0.1 (-0.1,0.4)	0.1 (-0.1,0.4)	-0.1 (-2.1,2)	-12 (-23,-1)	0.2 (-0.2,0.6)	9 (-1,20)	0.1 (-0.1,0.3)	-0.3 (-1.3,0.8)
	P trend	**0.03**	**<0.0001**	0.02	**0.002**	0.31	0.27	0.79	**0.009**	0.42	**0.009**	0.64	**0.270**
	Q1	Ref	Ref	Ref	Ref	Ref	Ref	Ref	Ref	Ref	Ref	Ref	Ref
"Prudent" Pattern	Q2	0.4 (-0.5,1.2)	-1 (-20,19)	-4 (-7,-1)	1 (-7,9)	0.1 (-0.1,0.4)	0.1 (-0.1,0.4)	0.3 (-1.3,2)	-4 (-12,3)	-0.2 (-0.6,0.2)	-2 (-12,9)	0 (-0.2,0.2)	-0.5 (-1.5,0.4)
	Q3	-0.5 (-1.3,0.3)	-3 (-25,19)	-4 (-7,-1)	11 (2,19)	0 (-0.3,0.2)	-0.1 (-0.3,0.1)	0.7 (-1,2.3)	7 (-1,16)	0.2 (-0.2,0.6)	4 (-7,15)	-0.2 (-0.4,0)	-0.7 (-1.5,0.1)
	Q4	-0.2 (-1.1,0.6)	-4 (-25,17)	-8 (-11,-5)	2 (-7,12)	0 (-0.2,0.2)	0.1 (-0.1,0.3)	0.4 (-1.3,2.1)	-4 (-11,4)	0 (-0.4,0.4)	-1 (-11,9)	-0.2 (-0.4,0)	-0.7 (-1.7,0.2)
	Q5	-0.2 (-1,0.7)	-7 (-29,15)	-6 (-10,-2)	7 (-1,16)	0.1 (-0.2,0.4)	0 (-0.2,0.3)	1 (-0.8,2.8)	-1 (-9,7)	-0.1 (-0.6,0.4)	5 (-6,16)	-0.2 (-0.4,0)	-0.4 (-1.4,0.6)
	P trend	0.90	0.68	**0.001**	0.07	0.56	0.91	**0.41**	0.98	0.90	0.26	0.02	0.18
	Q1	Ref	Ref	Ref	Ref	Ref	Ref	Ref	Ref	Ref	Ref	Ref	Ref
"Open-Sandwich" Pattern	Q2	-0.6 (-1.4,0.2)	-3 (-24,18)	1 (-3,5)	-7 (-17,2)	0.2 (-0.1,0.4)	0.2 (0,0.4)	-0.1 (-1.7,1.4)	-3 (-11,5)	-0.1 (-0.5,0.2)	-5 (-15,5)	0 (-0.3,0.2)	0 (-0.9,0.9)
	Q3	0 (-0.9,0.9)	-5 (-25,15)	0 (-3,4)	-5 (-15,4)	0.2 (0,0.4)	0.2 (0,0.4)	0.6 (-1.1,2.4)	-3 (-12,5)	-0.3 (-0.7,0.1)	-13 (-22,-4)	0.1 (-0.1,0.3)	-0.1 (-1,0.8)
	Q4	-0.2 (-1.2,0.8)	-18 (-41,5)	3 (-1,8)	-3 (-13,7)	0.2 (0,0.5)	0 (-0.2,0.3)	1.1 (-0.4,2.5)	1 (-7,8)	-0.4 (-0.8,0)	-16 (-27,-5)	0 (-0.2,0.2)	0.6 (-0.4,1.6)
	Q5	0.8 (0,1.5)	-14 (-39,10)	2 (-2,7)	-5 (-15,4)	0.3 (0,0.5)	0.1 (-0.1,0.4)	1.8 (0,3.5)	-2 (-10,7)	-0.5 (-0.9,0)	-16 (-26,-6)	-0.1 (-0.3,0.2)	0.6 (-0.3,1.5)
	P trend	0.82	0.38	**0.21**	**0.73**	0.04	0.83	0.06	0.47	**0.02**	**0.01**	0.93	0.10
	Q1	Ref	Ref	Ref	Ref	Ref	Ref	Ref	Ref	Ref	Ref	Ref	Ref

©2020 Nassan FL et al. *JAMA Network Open*.

**“Vegetarian-like” Pattern**	Q2	-0.2 (-0.9,0.6)	5 (-15,26)	4 (0,8)	-6 (-16,4)	0 (-0.3,0.2)	0.1 (-0.2,0.3)	0.5 (-1,2)	-9 (-17,-1)	0.4 (0,0.9)	7 (-2,17)	-0.1 (-0.3,0.1)	-0.3 (-1.3,0.8)
	Q3	1.2 (0.4,2)	3 (-19,24)	4 (0,7)	-5 (-14,4)	-0.1 (-0.3,0.1)	0.2 (-0.1,0.4)	0.2 (-1.2,1.6)	-11 (-19,-3)	-0.1 (-0.5,0.2)	0 (-9,9)	0.1 (-0.2,0.3)	0.4 (-0.4,1.3)
	Q4	1.1 (0.4,1.9)	4 (-15,24)	4 (0,9)	-5 (-15,6)	0.1 (-0.2,0.3)	-0.1 (-0.3,0.2)	1.8 (0.4,3.3)	-2 (-10,7)	0.3 (0,0.7)	2 (-8,11)	-0.1 (-0.3,0.1)	0.1 (-0.9,1.1)
	Q5	1.1 (-0.4,2.5)	13 (-9,35)	3 (0,7)	-15 (-24,-7)	-0.1 (-0.3,0.1)	0 (-0.3,0.3)	1.6 (0.2,2.9)	-9 (-17,-2)	0.5 (0.1,0.9)	7 (-3,18)	-0.1 (-0.3,0.1)	-0.5 (-1.4,0.3)
	P trend	0.003	0.08	0.18	**0.0003**	0.48	0.29	**0.04**	0.53	0.02	0.11	0.31	0.15

Abbreviations: Q; quintile, 95% CI; 95% confidence intervals, LH; Luteinizing Hormone, FSH; Follicle stimulating hormone, SHBG; Sex Hormone Binding Globulin, Ref; reference. Models were adjusted for age, height, smoking, use of marijuana and other recreational drug, moderate-to-vigorous physical activities (hours/ week), history of reproductive diseases, reproductive surgeries, and sexually transmitted diseases, season and calendar year of the sample, mother’s education level, total energy intake, and time of the day of the sample collection.

©2020 Nassan FL et al. *JAMA Network Open*.

eTable 15. Adjusted Median Differences in Semen Quality Parameters and Testicular Volume According to Adherence to Data-Derived Dietary Patterns Among 1175 Men Who Were Never Smokers, Never Marijuana Users, and Nonusers of Other Recreational Drugs

Adjusted median difference among never smokers, never marijuana users and non-users of other recreational drugs among 1175 men

		Semen volume, mL	Sperm concentration, million/mL	Total sperm count, million	Motile spermatozoa, %	Total motile sperm count, million	Progressively motile spermatozoa, %	Total progressively motile sperm count, million	Normal sperm morphology, %	Total normal morphologically sperm count, million	Testicular volume by ultrasound, mean (mL)
	Q1	Ref	Ref	Ref	Ref	Ref	Ref	Ref	Ref	Ref	
"Western" Pattern	Q2	-0.2 (-0.4,0.1)	1 (-6.7,8.6)	-3 (-26,20)	-0.1 (-2,1.9)	-6 (-23,10)	0.6 (-2.3,3.5)	-6 (-21,9)	0.2 (-0.7,1.2)	-0.7 (-1.4,-0.1)	
	Q3	-0.3 (-0.6,-0.1)	-0.1 (-8.5,8.2)	-6 (-30,19)	2.1 (-0.1,4.3)	-14 (-30,3)	2.3 (-0.7,5.3)	-5 (-21,11)	0.3 (-0.9,1.4)	-0.9 (-1.5,-0.2)	
	Q4	0.0 (-0.2,0.2)	-0.1 (-8.2,8)	-4 (-28,21)	1.4 (-1.4,4.1)	-5 (-24,13)	-0.2 (-3.2,2.9)	-3 (-20,15)	-1.3 (-2.2,-0.4)	-0.2 (-1.1,0.6)	
	Q5	-0.3 (-0.4,-0.1)	-6.5 (-16.5,3.4)	-30 (-60,-1)	1.8 (-1.4,5)	-22 (-44,0)	-0.1 (-3.9,3.7)	-15 (-34,4)	-1.2 (-2.4,0.0)	-1 (-1.9,-0.1)	
	P trend	0.18	0.15	0.03	0.40	0.13	0.61	0.21	0.006	0.08	
	Q1	Ref	Ref	Ref	Ref	Ref	Ref	Ref	Ref	Ref	
"Prudent" Pattern	Q2	0.1 (-0.2,0.3)	4.9 (-4.4,14.2)	40 (17,63)	-1.9 (-5.7,2.0)	23 (7,39)	-0.4 (-4.2,3.4)	22 (7,38)	-0.6 (-1.9,0.6)	0.4 (-0.2,1.1)	
	Q3	0.3 (0.1,0.5)	7.7 (-0.7,16.1)	61 (38,83)	-0.2 (-3.6,3.2)	37 (21,52)	0.8 (-2.2,3.9)	36 (21,50)	-0.4 (-1.5,0.8)	0.7 (-0.1,1.5)	
	Q4	0.2 (-0.1,0.5)	8.0 (-3.4,19.4)	69 (45,92)	0.2 (-3.0,3.4)	41 (18,63)	-0.4 (-3.4,2.7)	43 (21,65)	0 (-1.3,1.2)	0.3 (-0.4,1.0)	
	Q5	0.5 (0.2,0.7)	9.7 (0.2,19.2)	83 (51,115)	0.0 (-3.2,3.2)	50 (29,70)	1.4 (-1.4,4.3)	46 (28,64)	0.3 (-0.9,1.4)	0.5 (-0.4,1.3)	
	P trend	0.001	0.11	<0.0001	0.63	<0.0001	0.72	<0.0001	0.51	0.39	
	Q1	Ref	Ref	Ref	Ref	Ref	Ref	Ref	Ref	Ref	
"Open-Sandwich" Pattern	Q2	-0.2 (-0.4,0.0)	-4.9 (-15.2,5.4)	-15 (-44,15)	-1.0 (-3.7,1.6)	-16 (-35,3)	-1.0 (-4.3,2.3)	-17 (-33,0)	-0.4 (-1.3,0.5)	-1.3 (-2.0,-0.6)	
	Q3	-0.2 (-0.4,0.0)	-2.7 (-12.8,7.4)	-4 (-33,24)	2.7 (-0.1,5.4)	-5 (-31,21)	1.8 (-1.2,4.9)	-2 (-24,20)	0.1 (-1,1.2)	-0.7 (-1.4,0.0)	
	Q4	0 (-0.2,0.2)	-3.7 (-13.6,6.3)	-5 (-39,29)	1.4 (-1.3,4.1)	-10 (-35,15)	-0.5 (-3.5,2.5)	-6 (-27,15)	-0.4 (-1.5,0.7)	-0.8 (-1.5,-0.1)	
	Q5	0 (-0.3,0.2)	-7.1 (-16,1.8)	-8 (-38,21)	2.6 (-0.1,5.4)	-12 (-34,11)	0.7 (-2.6,4.1)	-6 (-25,13)	-1.0 (-2.1,0.2)	-1.1 (-1.9,-0.3)	
	P trend	0.19	0.07	0.45	0.07	0.67	0.67	0.91	0.16	0.12	
	Q1	Ref	Ref	Ref	Ref	Ref	Ref	Ref	Ref	Ref	
"Vegetarian-like" Pattern	Q2	0.1 (-0.1,0.3)	-2.7 (-11.1,5.6)	0 (-22,21)	5.5 (2.9,8)	4 (-16,23)	3.6 (0.1,7.1)	3 (-14,21)	1.1 (0.0,2.2)	-0.4 (-1.1,0.3)	
	Q3	0.1 (-0.2,0.3)	0.7 (-7.5,8.9)	13 (-11,37)	2.6 (0.4,4.7)	8 (-11,27)	2.6 (-0.8,5.9)	11 (-4,26)	0.5 (-0.6,1.6)	0 (-0.7,0.7)	

©2020 Nassan FL et al. *JAMA Network Open*.

	Q4	0.1 (-0.2,0.4)	2.6 (-6.4,11.5)	18 (-6,43)	1.2 (-0.8,3.1)	5 (-11,21)	0.4 (-2.9,3.7)	9 (-5,24)	0.1 (-1.0,1.1)	0.1 (-0.6,0.7)
	Q5	0.3 (0.1,0.4)	-1.7 (-11.2,7.7)	8 (-22,38)	2.3 (-0.2,4.7)	10 (-7,27)	0.6 (-3.1,4.2)	10 (-6,27)	1.1 (-0.1,2.4)	0.5 (-0.2,1.2)
	P trend	0.07	0.61	0.90	0.44	0.92	0.54	0.75	0.66	0.18

Count of morphologically normal spermatozoa model did not converge.

Abbreviations: 95% CI; 95% confidence intervals.

Models were adjusted for age, body mass index, height, moderate-to-vigorous physical activities (hours/ week), history of reproductive diseases, reproductive surgeries, and sexually transmitted diseases, season and calendar year of the sample, mother's education level, total energy intake, and abstinence time. Sperm motility models were further adjusted for time elapsed between specimen collection and analysis.

* Tests for trend were conducted across quintiles using the median value in each quintile of the diet patterns as a continuous variable in the regression models.

©2020 Nassan FL et al. *JAMA Network Open*.

eTable 16. Adjusted Median Differences in Serum Reproductive Hormone Concentrations on the Scale for the Individual Hormones as Stated in the Table According to Adherence to Data-Derived Dietary Patterns, Among 1175 Men Who Were Never Smokers, Never Marijuana Users, and Nonusers Of Other Recreational Drugs

	**Reproductive Parameters**	**Total Testosterone, nmol/L**	**Free Testosterone, pmol/L**	**Estradiol, pg/mL**	**Inhibin-B, pg/mL**	**LH, IU/L**	**FSH, IU/L**	**SHBG, nmol/L**	**Inhibin-B/ FSH**	**Total Testosterone/ LH**	**Free Testosterone/ LH**	**Estradiol/ Total Testosterone**	**(Estradiol/ Free Testosterone)*100**
	Q1	Ref	Ref	Ref	Ref	Ref	Ref	Ref	Ref	Ref	Ref	Ref	Ref
**“Western” Pattern**	Q2	0.8 (-0.1,1.7)	27 (4,50)	4 (0,8)	-6 (-15,4)	0.2 (-0.1,0.4)	0.3 (0.1,0.6)	2.4 (0.5,4.3)	-9 (-19,1)	0 (-0.5,0.5)	3 (-7,14)	-0.3 (-0.6,-0.1)	-0.9 (-1.9,0.1)
	Q3	0.4 (-0.6,1.5)	11 (-15,37)	0 (-5,5)	-5 (-15,5)	0.1 (-0.1,0.4)	0.3 (0,0.6)	1.1 (-1,3.2)	-6 (-19,6)	0.1 (-0.5,0.6)	1 (-9,10)	-0.5 (-0.7,-0.2)	-1.2 (-2.4,0.1)
	Q4	0.5 (-0.5,1.4)	19 (-4,42)	-3 (-8,2)	-10 (-23,4)	0.2 (-0.2,0.6)	0.4 (0.1,0.8)	0.4 (-1.7,2.6)	-17 (-28,-6)	-0.1 (-0.8,0.5)	-13 (-27,1)	-0.2 (-0.5,0.1)	-0.1 (-1.3,1.1)
	Q5	0.2 (-0.9,1.2)	31 (0,62)	3 (-2,7)	-20 (-31,-9)	0.2 (-0.1,0.4)	0.2 (-0.1,0.6)	1 (-1.2,3.2)	-18 (-31,-5)	0 (-0.6,0.5)	0 (-12,13)	-0.2 (-0.5,0)	-1.4 (-2.4,-0.3)
	P trend	**0.77**	**0.09**	0.84	**0.02**	0.46	0.17	0.72	**0.003**	0.79	**0.70**	0.13	**0.02**
	Q1	Ref	Ref	Ref	Ref	Ref	Ref	Ref	Ref	Ref	Ref	Ref	Ref
**“Prudent” Pattern**	Q2	1.1 (-0.2,2.4)	11 (-19,41)	2 (-4,8)	5 (-7,17)	0.1 (-0.2,0.4)	0.3 (0,0.6)	2.3 (0,4.6)	-6 (-20,7)	-0.1 (-0.7,0.5)	-4 (-15,8)	-0.2 (-0.5,0.1)	-0.4 (-1.7,0.9)
	Q3	1.2 (-0.1,2.5)	-8 (-39,24)	-2 (-6,3)	15 (5,25)	-0.1 (-0.4,0.1)	-0.3 (-0.6,-0.1)	3.2 (1,5.3)	14 (1,26)	0.2 (-0.4,0.8)	6 (-6,18)	-0.3 (-0.6,0)	-0.7 (-1.7,0.4)
	Q4	0.5 (-0.9,1.9)	10 (-22,41)	-2 (-7,3)	0 (-12,11)	0 (-0.3,0.3)	0.3 (0.1,0.5)	3.1 (1,5.2)	-9 (-21,3)	-0.2 (-0.9,0.5)	-8 (-19,4)	-0.2 (-0.5,0.1)	-0.4 (-1.7,1)
	Q5	0.4 (-0.8,1.7)	-12 (-43,20)	-1 (-7,5)	3 (-10,16)	0.1 (-0.1,0.4)	0.2 (-0.1,0.6)	4.6 (2.1,7.1)	-5 (-16,7)	0.2 (-0.5,0.8)	-1 (-12,10)	-0.2 (-0.6,0.1)	0.1 (-1.2,1.4)
	P trend	0.97	0.23	**0.23**	0.70	0.86	0.35	**0.002**	0.24	0.74	0.90	0.54	0.91
	Q1	Ref	Ref	Ref	Ref	Ref	Ref	Ref	Ref	Ref	Ref	Ref	Ref
**“Open-Sandwich” Pattern**	Q2	-0.3 (-1.3,0.8)	19 (-10,49)	3 (-2,8)	-8 (-19,3)	0.1 (-0.1,0.4)	0 (-0.3,0.4)	-0.7 (-2.7,1.2)	-2 (-14,11)	-0.4 (-1.1,0.2)	-3 (-19,12)	0.1 (-0.2,0.3)	-0.2 (-1.4,1)
	Q3	0.4 (-0.7,1.5)	21 (-10,53)	4 (-1,9)	-4 (-15,8)	0.3 (0,0.5)	-0.2 (-0.5,0.2)	0.4 (-1.9,2.7)	4 (-10,19)	-0.8 (-1.5,-0.1)	-9 (-22,5)	0 (-0.3,0.3)	0.1 (-1.1,1.3)
	Q4	0.4 (-0.7,1.5)	3 (-28,35)	3 (-2,8)	-7 (-17,3)	0.4 (0.1,0.6)	-0.1 (-0.5,0.2)	1.5 (-0.8,3.9)	3 (-10,17)	-0.6 (-1.3,0)	-12 (-27,3)	0 (-0.3,0.2)	0.6 (-0.8,2)
	Q5	0.7 (-0.6,2.1)	11 (-22,43)	1 (-4,6)	-17 (-28,-6)	0.4 (0.1,0.7)	0.1 (-0.2,0.5)	1.9 (-0.6,4.5)	-10 (-23,3)	-1 (-1.7,-0.3)	-16 (-31,-1)	-0.2 (-0.5,0)	-0.3 (-1.4,0.8)
	P trend	0.08	0.63	**0.38**	**0.04**	0.01	0.55	0.12	0.09	**0.02**	**0.02**	0.04	0.37
	Q1	Ref	Ref	Ref	Ref	Ref	Ref	Ref	Ref	Ref	Ref	Ref	Ref
**“Veg[illegible]**	Q2	1.5 (0.6,2.3)	27 (4,49)	4 (-1,8)	-1 (-13,10)	0.4 (0.1,0.7)	0.1 (-0.2,0.3)	0.1 (-1.8,2.1)	-9 (-22,3)	0.1 (-0.4,0.6)	0 (-11,11)	-0.1 (-0.3,0.2)	-1.1 (-2.5,0.3)

©2020 Nassan FL et al. *JAMA Network Open*.

	Q3	0.8 (-0.2,1.7)	22 (-2,47)	2 (-2,7)	7 (-2,16)	0.2 (-0.1,0.6)	0.1 (-0.1,0.4)	2.2 (0.1,4.3)	-6 (-18,6)	0.1 (-0.5,0.6)	-3 (-14,7)	-0.1 (-0.4,0.2)	-0.2 (-1.6,1.1)
	Q4	0.7 (-0.3,1.8)	15 (-15,45)	3 (-2,8)	7 (-4,18)	0.1 (-0.2,0.5)	0.1 (-0.2,0.4)	1.4 (-0.4,3.1)	-3 (-17,11)	0.2 (-0.4,0.8)	1 (-10,13)	0 (-0.3,0.3)	0.2 (-1.1,1.4)
	Q5	0.9 (-0.2,2)	30 (-2,63)	0 (-4,5)	-4 (-13,6)	0 (-0.3,0.2)	0.1 (-0.2,0.3)	0.8 (-1.4,3.1)	-9 (-22,3)	0.6 (0.0,1.2)	13 (1,25)	-0.2 (-0.4,0.1)	-1.3 (-2.5,0)
	P trend	0.308	0.35	0.84	**0.87**	0.29	0.95	**0.17**	0.62	0.08	0.19	0.48	0.24

Abbreviations: Q; quintile, 95% CI; 95% confidence intervals, LH; Luteinizing Hormone, FSH; Follicle stimulating hormone, SHBG; Sex Hormone Binding Globulin, Ref; reference.
Models were adjusted for age, height, moderate-to-vigorous physical activities (hours/ week), history of reproductive diseases, reproductive surgeries, and sexually transmitted diseases, season and calendar year of the sample, mother's education level, total energy intake, and time of the day of the sample collection.

©2020 Nassan FL et al. *JAMA Network Open*.

eTable 17. Demographic Characteristics of Men Who Were and were not included in the Analysis

CHARACTERISTICS	MEDIAN (IQR) OR N (%)	
	Participants not included in the analysis	Participants included in the analysis
AGE, YEARS	19(19,21)	19(19,20)
BODY MASS INDEX, KG/M^2	22(21,25)	22(21,24)
HEIGHT, M	1.81(1.75,1.84)	1.82(1.77,1.87)
CIGARETTES SMOKING, N (%)		
DAILY	25(31)	787(26.81)
OCCASIONALLY	14(17)	683(23.27)
NEVER	42(52)	1465(49.91)
MARIJUANA SMOKING, N (%)		
DAILY	5(6)	110(3.75)
OCCASIONALLY	22(27)	989(33.7)
NEVER	54(67)	1836(62.56)
OTHER RECREATIONAL DRUG USE, N (%)	15(19)	345(11.75)
EDUCATION OF THE MOTHER		
< 9 YEARS	3(4)	92(3.13)
9-10 YEARS	19(23)	725(24.7)
> 11 YEARS	36(44)	1665(56.73)
OTHER	3(4)	183(6.24)
MISSING/DO NOT KNOW	20(25)	270(9.2)
MODERATE AND VIGOROUS PHYSICAL ACTIVITIES, HR/WK	6(2,11)	8(4,14)
FEVER IN LAST 3 MONTHS, N (%)	3(4)	239(8.14)
SELF-REPORTED HISTORY OF REPRODUCTIVE DISEASES, N (%)	11(14)	605(20.61)
SELF-REPORTED HISTORY OF REPRODUCTIVE SURGERY, N (%)	6(7)	332(11.31)
SELF-REPORTED HISTORY OF STDS, N (%)	12(15)	333(11.35)
USE OF MUSCLE-ENHANCING PRODUCTS IN THE LAST 3 MONTHS, N (%)	49(65)	752(25.74)
ABSTINENCE TIME, HOURS	62(53,84)	62(57,84)
SAMPLE COLLECTED DURING WARM SEASON, N (%)	26(32)	950(32.37)
TIME OF DAY OF SAMPLE COLLECTION, HOUR	10(10,11)	10(9,11)
TIME TO MOTILITY ANALYSIS, MIN	30(25,50)	30(20,45)

©2020 Nassan FL et al. *JAMA Network Open*.

**TOTAL ENERGY INTAKE, KCAL/DAY**	2223(1447,6339)	1990(1536,2547)
**TOTAL DIETARY FAT (% OF ENERGY)**	33(27,39)	34(30,38)
**SATURATED DIETARY FAT (% OF ENERGY)**	13(10,15)	13(11,15)
**MONOUNSATURATED DIETARY FAT (% OF ENERGY)**	11(9,13)	12(10,13)
**POLYUNSATURATED DIETARY FAT (% OF ENERGY)**	5(4,6)	5(4,6)
**DIETARY PROTEIN (% OF ENERGY)**	17(15,20)	17(16,20)
**DIETARY CARBOHYDRATE (% OF ENERGY)**	50(42,54)	48(43,53)

Abbreviations: N; number of men, m; meters, hr; hours, wk; week, STDs; sexually transmitted Diseases, α-TE ; alpha-tocopherol equivalents.

Numbers shown are median and interquartile range unless noted as N(%).

Warm season: April through September.

Reproductive diseases include self-reported history of varicocele, cryptorchidism, testicular mumps, inguinal hernia, testicular injury (hit, kicked or otherwise injured so it caused swelling of the scrotum), hydrocele, testicular torsion, hypospadias, epididymo-orchites, cystitis or prostatitis.

Reproductive surgeries include self-reported history of surgery for inguinal hernia, varicocele, hydrocele, testicular torsion, hypospadias, testicular cancer, phimosis, testicular biopsy, vasectomy, re-fertilization, and other reproductive surgeries.

STDs included self-reported history of gonorrhea, chlamydia, and other venereal diseases.

©2020 Nassan FL et al. *JAMA Network Open*.
